# Supplementary material for: Revealing the Response Mechanism of Pediococcus pentosaceus Under Acid and Alcohol Stresses via a Combined Transcriptomic and Metabolomic Analysis
Source: Foods. 2025 Jul 7;14(13):2400. doi: 10.3390/foods14132400 (PMC12249401; doi:10.3390/foods14132400)
Supplement: Supplementary file 1 [file foods-14-02400-s001.zip › foods-3713726-supplementary.pdf]

Supplementary figure captions

Figure S1 Classification statistics of KEGG enrichment results. (a) Gene sets B vs A; (b) gene sets C vs A; (c) gene sets D vs A.

Figure S2 Bubble charts of KEGG pathway enrichment for different gene sets. (a) gene sets B vs A, (b) gene sets C vs A, (c) gene sets D vs A. Bubble size: Number of genes (the number of genes in the gene set that are associated with the pathway). Bubble color: p-value (the statistical significance of the enrichment, with different colors representing different p-value ranges).

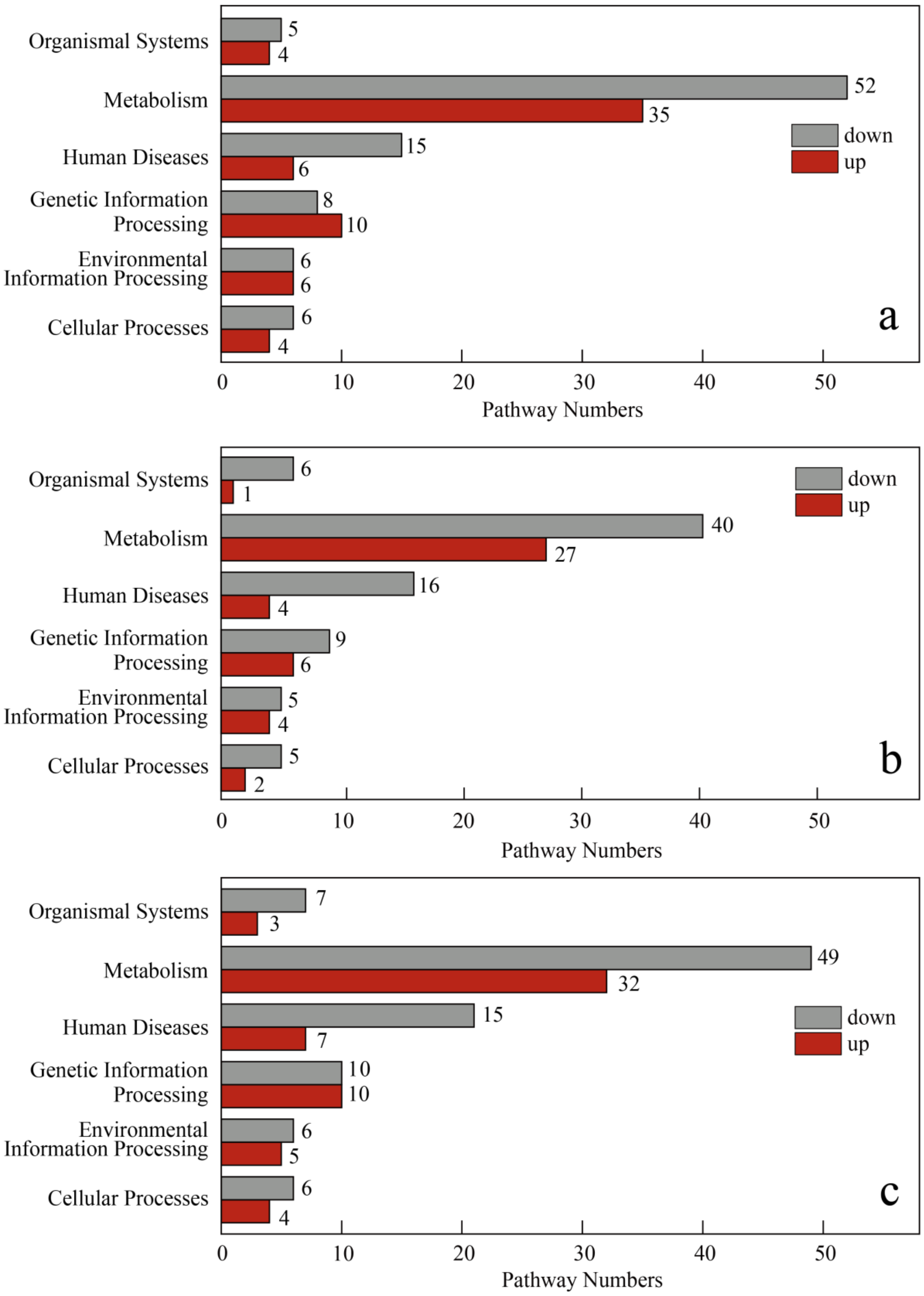
Organismal Systems
Metabolism
Human Diseases
Genetic Information Processing
Environmental Information Processing
Cellular Processes
5
4
52
35
15
6
8
10
6
6
6
4
down
up
a
0
10
20
30
40
50
Pathway Numbers
Organismal Systems
Metabolism
Human Diseases
Genetic Information Processing
Environmental Information Processing
Cellular Processes
6
1
40
27
16
4
9
6
5
4
5
2
down
up
b
0
10
20
30
40
50
Pathway Numbers
Organismal Systems
Metabolism
Human Diseases
Genetic Information Processing
Environmental Information Processing
Cellular Processes
7
3
49
32
15
7
10
10
6
5
6
4
down
up
c
0
10
20
30
40
50
Pathway Numbers

Figure S1

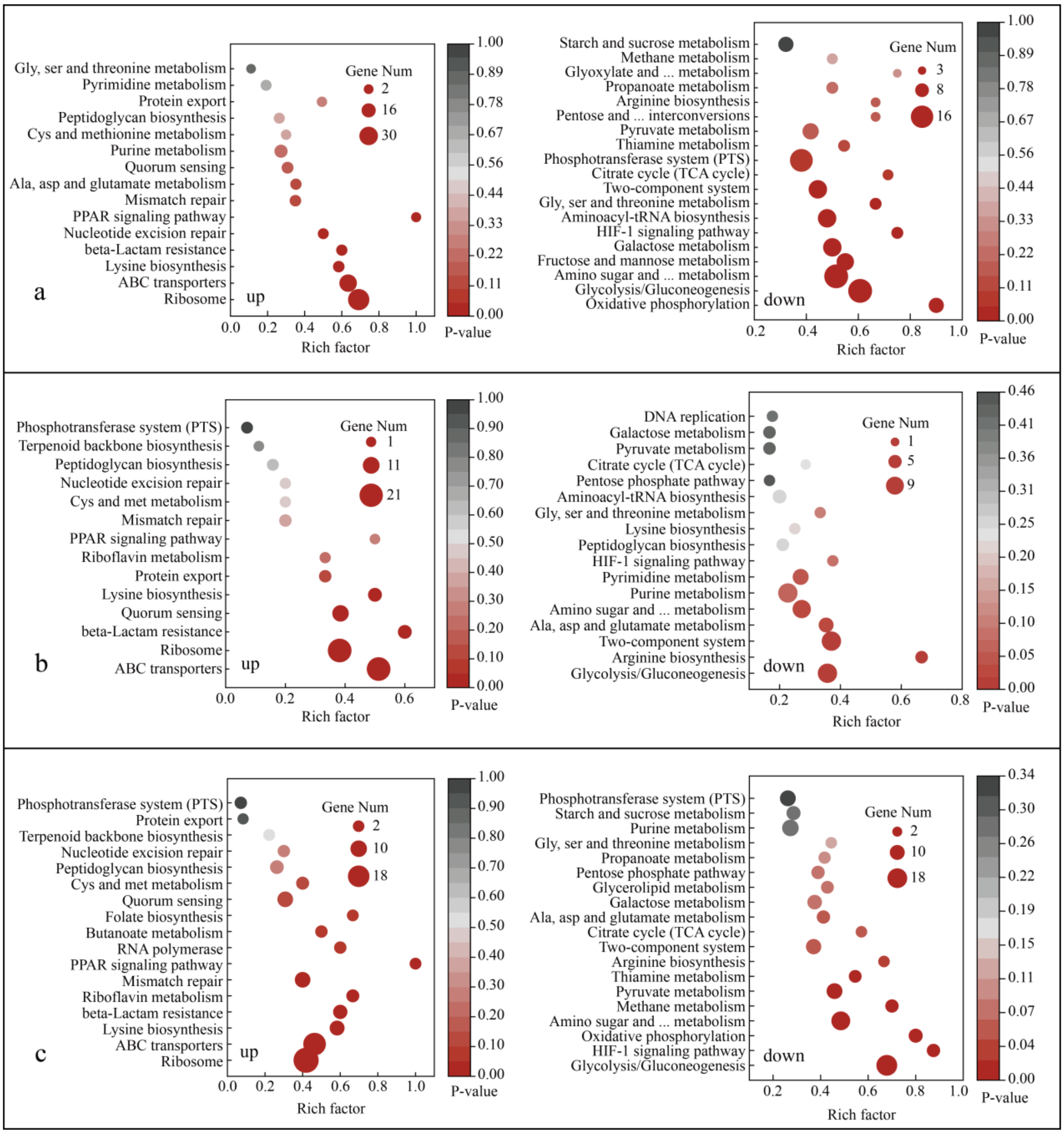
a
Gly, ser and threonine metabolism
Pyrimidine metabolism
Protein export
Peptidoglycan biosynthesis
Cys and methionine metabolism
Purine metabolism
Quorum sensing
Ala, asp and glutamate metabolism
Mismatch repair
PPAR signaling pathway
Nucleotide excision repair
beta-Lactam resistance
Lysine biosynthesis
ABC transporters
Ribosome
up
Gene Num
2
16
30
Rich factor
P-value
Starch and sucrose metabolism
Methane metabolism
Glyoxylate and ... metabolism
Propanoate metabolism
Arginine biosynthesis
Pentose and ... interconversions
Pyruvate metabolism
Thiamine metabolism
Phosphotransferase system (PTS)
Citrate cycle (TCA cycle)
Two-component system
Gly, ser and threonine metabolism
Aminoacyl-tRNA biosynthesis
HIF-1 signaling pathway
Galactose metabolism
Fructose and mannose metabolism
Amino sugar and ... metabolism
Glycolysis/Gluconeogenesis
Oxidative phosphorylation
down
Gene Num
3
8
16
Rich factor
P-value
b
Phosphotransferase system (PTS)
Terpenoid backbone biosynthesis
Peptidoglycan biosynthesis
Nucleotide excision repair
Cys and met metabolism
Mismatch repair
PPAR signaling pathway
Riboflavin metabolism
Protein export
Lysine biosynthesis
Quorum sensing
beta-Lactam resistance
Ribosome
ABC transporters
up
Gene Num
1
11
21
Rich factor
P-value
DNA replication
Galactose metabolism
Pyruvate metabolism
Citrate cycle (TCA cycle)
Pentose phosphate pathway
Aminoacyl-tRNA biosynthesis
Gly, ser and threonine metabolism
Lysine biosynthesis
Peptidoglycan biosynthesis
HIF-1 signaling pathway
Pyrimidine metabolism
Purine metabolism
Amino sugar and ... metabolism
Ala, asp and glutamate metabolism
Two-component system
Arginine biosynthesis
Glycolysis/Gluconeogenesis
down
Gene Num
1
5
9
Rich factor
P-value
c
Phosphotransferase system (PTS)
Protein export
Terpenoid backbone biosynthesis
Nucleotide excision repair
Peptidoglycan biosynthesis
Cys and met metabolism
Quorum sensing
Folate biosynthesis
Butanoate metabolism
RNA polymerase
PPAR signaling pathway
Mismatch repair
Riboflavin metabolism
beta-Lactam resistance
Lysine biosynthesis
ABC transporters
Ribosome
up
Gene Num
2
10
18
Rich factor
P-value
Phosphotransferase system (PTS)
Starch and sucrose metabolism
Purine metabolism
Gly, ser and threonine metabolism
Propanoate metabolism
Pentose phosphate pathway
Glycerolipid metabolism
Galactose metabolism
Ala, asp and glutamate metabolism
Citrate cycle (TCA cycle)
Two-component system
Arginine biosynthesis
Thiamine metabolism
Pyruvate metabolism
Methane metabolism
Amino sugar and ... metabolism
Oxidative phosphorylation
HIF-1 signaling pathway
Glycolysis/Gluconeogenesis
down
Gene Num
2
10
18
Rich factor
P-value

Figure S2

Table S1 The elution gradient for ultra-performance liquid chromatography

Time (min)	A (%)	B (%)
**0**	95	5
**2**	95	5
**4**	70	30
**8**	50	50
**10**	20	80
**14**	0	100
**15**	0	100
**15.1**	95	5
**16**	95	5

Table S2 Mass spectrum parameter for metabolomic analysis

Parameter	Positive ion	Negative ion
**Spray Voltage (V)**	3800	-3000
**Capillary Temperature (°C)**	320	320
**Aux gas heater temperature (℃)**	350	350
**Sheath Gas Flow Rate (Arb)**	35	35
**Aux gas flow rate (Arb)**	8	8
**S-lens RF level**	50	50
**Mass range (m/z)**	100-1200	100-1200
**Full ms resolution**	70000	70000
**MS/MS resolution**	17500	17500
**NCE/stepped NCE**	10, 20, 40	10, 20, 40

Table S3 GO enrichment results of gene sets B vs A

GO ID	Function Description	Gene Num
GO:0005198	structural molecule activity	39
GO:0003735	structural constituent of ribosome	39
GO:0043604	amide biosynthetic process	47
GO:0043603	cellular amide metabolic process	49
GO:0006412	translation	42
GO:0043043	peptide biosynthetic process	42
GO:0006518	peptide metabolic process	42
GO:0005840	ribosome	34
GO:0044267	cellular protein metabolic process	44
GO:1901566	organonitrogen compound biosynthetic process	92
GO:0019843	rRNA binding	31
GO:0046451	diaminopimelate metabolic process	9
GO:0009089	lysine biosynthetic process via diaminopimelate	9
GO:0009085	lysine biosynthetic process	9
GO:0006553	lysine metabolic process	9
GO:1901564	organonitrogen compound metabolic process	127
GO:0005215	transporter activity	86
GO:0022857	transmembrane transporter activity	86
GO:0043232	intracellular non-membrane-bounded organelle	35
GO:0043229	intracellular organelle	35
GO:0043228	non-membrane-bounded organelle	35
GO:0043226	organelle	35
GO:0044271	cellular nitrogen compound biosynthetic process	85
GO:0046933	proton-transporting ATP synthase activity, rotational mechanism	8
GO:0015252	proton channel activity	8
GO:0015399	primary active transmembrane transporter activity	27
GO:0042626	ATPase-coupled transmembrane transporter activity	27
GO:0006090	pyruvate metabolic process	10
GO:0022804	active transmembrane transporter activity	45
GO:0015078	proton transmembrane transporter activity	11
GO:0009132	nucleoside diphosphate metabolic process	9
GO:0046034	ATP metabolic process	9
GO:0043648	dicarboxylic acid metabolic process	9
GO:0016310	phosphorylation	9
GO:0006790	sulfur compound metabolic process	18
GO:0019538	protein metabolic process	44
GO:0006757	ATP generation from ADP	8
GO:0009135	purine nucleoside diphosphate metabolic process	8
GO:0009179	purine ribonucleoside diphosphate metabolic process	8
GO:0046939	nucleotide phosphorylation	8
GO:0046031	ADP metabolic process	8

GO:0009185	ribonucleoside diphosphate metabolic process	8
GO:0006096	glycolytic process	8
GO:0006165	nucleoside diphosphate phosphorylation	8
GO:0009058	biosynthetic process	133
GO:0043436	oxoacid metabolic process	52
GO:0019752	carboxylic acid metabolic process	52
GO:0006094	gluconeogenesis	6
GO:0019319	hexose biosynthetic process	6

Table S4 GO enrichment results of gene sets C vs A

GO ID	Function Description	Gene Num
GO:0046451	diaminopimelate metabolic process	8
GO:0009089	lysine biosynthetic process via diaminopimelate	8
GO:0009085	lysine biosynthetic process	8
GO:0006553	lysine metabolic process	8
GO:0043648	dicarboxylic acid metabolic process	8
GO:0009067	aspartate family amino acid biosynthetic process	10
GO:1901607	alpha-amino acid biosynthetic process	15
GO:0006820	anion transport	5
GO:0015698	inorganic anion transport	5
GO:0016491	oxidoreductase activity	42
GO:1901605	alpha-amino acid metabolic process	22
GO:0044205	'de novo' UMP biosynthetic process	6
GO:0008652	cellular amino acid biosynthetic process	15
GO:0006817	phosphate ion transport	4
GO:0005315	inorganic phosphate transmembrane transporter activity	4
GO:0005840	ribosome	23
GO:0009066	aspartate family amino acid metabolic process	10
GO:0016627	oxidoreductase activity, acting on the CH-CH group of donors	5
GO:0055085	transmembrane transport	14
GO:0043232	intracellular non-membrane-bounded organelle	25
GO:0043229	intracellular organelle	25
GO:0043228	non-membrane-bounded organelle	25
GO:0043226	organelle	25
GO:0006525	arginine metabolic process	7
GO:0009174	pyrimidine ribonucleoside monophosphate biosynthetic process	6
GO:0009173	pyrimidine ribonucleoside monophosphate metabolic process	6
GO:0046049	UMP metabolic process	6
GO:0006222	UMP biosynthetic process	6
GO:0009065	glutamine family amino acid catabolic process	3
GO:0015833	peptide transport	3
GO:0006527	arginine catabolic process	3

GO:0042886	amide transport	3
GO:0019877	diaminopimelate biosynthetic process	4
GO:0006207	'de novo' pyrimidine nucleobase biosynthetic process	4
GO:0043650	dicarboxylic acid biosynthetic process	4
GO:0015291	secondary active transmembrane transporter activity	10
GO:0005215	transporter activity	60
GO:0022857	transmembrane transporter activity	60
GO:0071705	nitrogen compound transport	15

Table S5 GO enrichment results of gene sets D vs A

GO ID	Function Description	Gene Num
GO:0046451	diaminopimelate metabolic process	8
GO:0009089	lysine biosynthetic process via diaminopimelate	8
GO:0009085	lysine biosynthetic process	8
GO:0006553	lysine metabolic process	8
GO:0043648	dicarboxylic acid metabolic process	8
GO:0009067	aspartate family amino acid biosynthetic process	10
GO:1901607	alpha-amino acid biosynthetic process	15
GO:0006820	anion transport	5
GO:0015698	inorganic anion transport	5
GO:0016491	oxidoreductase activity	42
GO:1901605	alpha-amino acid metabolic process	22
GO:0044205	'de novo' UMP biosynthetic process	6
GO:0008652	cellular amino acid biosynthetic process	15
GO:0006817	phosphate ion transport	4
GO:0005315	inorganic phosphate transmembrane transporter activity	4
GO:0005840	ribosome	23
GO:0009066	aspartate family amino acid metabolic process	10
GO:0016627	oxidoreductase activity, acting on the CH-CH group of donors	5
GO:0055085	transmembrane transport	14
GO:0043232	intracellular non-membrane-bounded organelle	25
GO:0043229	intracellular organelle	25
GO:0043228	non-membrane-bounded organelle	25
GO:0043226	organelle	25
GO:0006525	arginine metabolic process	7
GO:0009174	pyrimidine ribonucleoside monophosphate biosynthetic process	6
GO:0009173	pyrimidine ribonucleoside monophosphate metabolic process	6
GO:0046049	UMP metabolic process	6
GO:0006222	UMP biosynthetic process	6
GO:0009065	glutamine family amino acid catabolic process	3
GO:0015833	peptide transport	3
GO:0006527	arginine catabolic process	3

GO:0042886	amide transport	3
GO:0019877	diaminopimelate biosynthetic process	4
GO:0006207	'de novo' pyrimidine nucleobase biosynthetic process	4
GO:0043650	dicarboxylic acid biosynthetic process	4
GO:0015291	secondary active transmembrane transporter activity	10
GO:0005215	transporter activity	60
GO:0022857	transmembrane transporter activity	60
GO:0071705	nitrogen compound transport	15

Table S6 Detailed information on differentially expressed genes

Gene ID	Gene name	Gene description	Expression A	B	C	D
**RS00005**	**dnaA**	**chromosomal replication initiator protein DnaA***	**159.01**	**27.11**	**100.63**	**28.84**
RS00010	dnaN	DNA polymerase III subunit beta	230.71	56.79	183.57	38.62
RS00015	yaaA	S4 domain-containing protein YaaA	346.94	481.68	286.33	141.25
RS00020	recF	DNA replication/repair protein RecF	435.96	221.89	258.5	145.85
RS00025	gyrB	DNA topoisomerase (ATP-hydrolyzing) subunit B	175.22	84.86	171.38	80.72
RS00030	gyrA	DNA gyrase subunit A	168.35	127.58	192.92	87.77
RS00035	rpsF	30S ribosomal protein S6	247.89	613.73	607.46	485.69
**RS00040**	**ssb**	**single-stranded DNA-binding protein**	**1854.97**	**3755.86**	**3990.3**	**4737.39**
RS00045	rpsR	30S ribosomal protein S18	291.29	533.04	602.68	541.38
RS00050	RS00050	DHH family phosphoesterase	144.89	173.15	230.12	195.96
RS00055	rplI	50S ribosomal protein L9	510.7	444.99	805.77	751.53
RS00060	dnaB	replicative DNA helicase	241.03	246.6	414.69	358.75
RS00065	RS00065	MFS transporter	84.84	203.54	341.05	381.61
RS00070	RS00070	DUF1304 domain-containing protein	561.17	390.18	426.08	194.69
RS00075	RS00075	sulfite exporter TauE/SafE family protein	18.76	17.37	42.15	28.34
RS00080	abc-f	ABC-F type ribosomal protection protein	489.19	278.11	416.24	210.29
RS00085	RS00085	TetR/AcrR family transcriptional regulator	70.65	256.75	141.23	166.84
RS00090	RS00090	ABC transporter permease	50.7	149.82	98.71	103.18
RS00095	RS00095	ABC transporter ATP-binding protein	53.16	140.4	91.97	114.47
RS00100	RS00100	helix-turn-helix domain-containing protein	16.87	7.44	30.13	14.5
RS00105	RS00105	GNAT family N-acetyltransferase	38.1	35.38	40.75	37.32
RS00110	RS00110	hypothetical protein	75.01	329.79	195.26	235.34
RS00115	RS00115	PTS transporter subunit EIIC	7.66	6.41	29.92	18.6
RS00120	RS00120	helix-turn-helix transcriptional regulator	107.05	141.86	96.07	40.54
RS00125	RS00125	aldo/keto reductase	41.59	46.92	124.81	78.03
RS00130	RS00130	SDR family oxidoreductase	93.11	68.46	165.72	154.78
RS00135	RS00135	NAD(P)H-dependent oxidoreductase	2991.21	5966.99	3094.57	3578.98
RS00140	RS00140	metalloregulator ArsR/SmtB family transcription factor	7.47	16.85	18.04	7.56
RS00145	RS00145	cadmium resistance transporter	26.53	102.41	38.93	32.04
RS00150	RS00150	LysM peptidoglycan-binding domain-containing protein	52.87	188.18	80.27	49.25
RS00155	RS00155	hypothetical protein	75.33	235.39	111.13	72.54
RS00160	RS00160	multidrug efflux SMR transporter	17.76	10.44	33.94	27.76
RS00165	RS00165	pentapeptide repeat-containing protein	49.84	28.1	79.09	72.64
RS00170	RS00170	FAD-dependent oxidoreductase	10.55	6.6	26.59	23.68
RS00175	RS00175	EAL domain-containing protein	6.24	3.02	10.31	11.53
RS00180	RS00180	DUF1836 domain-containing protein	35.46	48.18	220.34	78.1
RS00185	RS00185	hemolysin III family protein	39	254.66	186.93	270.78
RS00190	RS00190	GNAT family N-acetyltransferase	16.34	72.18	789.74	740.47
RS00195	RS00195	cation:dicarboxylase symporter family transporter	37.6	124.99	145.31	76.9
RS00200	RS00200	glycerophosphodiester phosphodiesterase	54.84	75.24	110.12	100.17
RS00205	RS00205	hypothetical protein	184.28	755.61	1056.64	575.32
RS00210	RS00210	CvpA family protein	12	71.86	99.18	69.28
RS00215	RS00215	VIT family protein	83.97	16.2	73.46	39.82
RS00220	RS00220	VIT family protein	52.11	13.48	39.67	30.93
RS00225	RS00225	Nramp family divalent metal transporter	365.15	587.29	1088.83	348

RS00230	RS00230	universal stress protein	631.12	823.05	1543.62	508.42
RS00235	RS00235	diacylglycerol kinase family lipid kinase	54.2	598.46	351.63	200.16
RS00240	RS00240	LysR family transcriptional regulator	37.68	902.86	469.88	230.45
RS00245	RS00245	acetyltransferase	57.21	36.96	64.33	51.35
RS00250	RS00250	SDR family oxidoreductase	55.71	32.8	51.3	47.61
RS00255	RS00255	aldo/keto reductase	36.46	32.48	38.48	42.95
RS00260	RS00260	hypothetical protein	55.15	14.31	26.38	26.13
RS00265	RS00265	ATP-binding cassette domain-containing protein	109.56	16.53	32.56	31.46
RS00270	RS00270	GntR family transcriptional regulator	256.09	26.42	55.56	36.86
RS00275	RS00275	serine hydrolase	35.43	43.1	70.08	42.22
RS00280	RS00280	zinc ribbon domain-containing protein	34.65	44.09	61.34	38.67
RS00285	RS00285	linear amide C-N hydrolase	279.72	260.15	353.63	199.98
RS00290	pnuC	nicotinamide riboside transporter PnuC	11.45	80.31	298.27	136.89
RS00300	RS00300	MerR family transcriptional regulator	7.43	49.59	114.47	47.82
RS00305	RS00305	aldo/keto reductase	100.1	40.96	61.62	42.38
RS00310	secA2	accessory Sec system translocase SecA2	14.85	11.8	15.28	14.94
RS00315	RS00315	preprotein translocase subunit SecY	12.25	9.11	11.44	12.56
RS00320	RS00320	accessory Sec system protein Asp1	15.52	14.93	15.82	14.28
RS00325	asp2	accessory Sec system protein Asp2	27.45	25.8	24.76	23.49
RS00330	asp3	accessory Sec system protein Asp3	23.61	16.71	16.85	16.52
RS00335	RS00335	glycosyltransferase	27.35	22.74	23.38	20.05
RS00340	RS00340	hypothetical protein	22.27	15.6	20.46	20.58
RS00345	RS00345	hypothetical protein	22.76	16.64	32.12	26.92
RS00350	RS00350	hypothetical protein	27.88	18.03	42.85	35.7
RS00365	RS00365	SDR family oxidoreductase	344.62	307.24	402.98	212.15
RS00370	RS00370	LysR family transcriptional regulator	24.03	39.2	88.29	31.46
RS00375	RS00375	aldo/keto reductase	32.01	38.29	81.31	75.35
RS00380	RS00380	alcohol dehydrogenase catalytic domain-containing protein	115.55	84.5	131.02	144.2
RS00420	RS00420	LytTR family DNA-binding domain-containing protein	29.45	24.33	36.16	34.87
RS00430	RS00430	metal ABC transporter solute-binding protein	16.9	17.08	19.15	13.02
RS00435	RS00435	hypothetical protein	8.08	41.82	33.06	19
RS00440	RS00440	GNAT family N-acetyltransferase	7.14	3.44	6.06	9.29
RS00445	RS00445	heavy metal translocating P-type ATPase	66.81	26.06	45.05	41.02
RS00450	RS00450	PTS transporter subunit EIIC	60.21	28.19	60.85	48.54
RS00455	RS00455	alpha/beta hydrolase	70.69	27.47	75.54	62.09
RS00465	RS00465	MurR/RpiR family transcriptional regulator	52.38	14.73	40.37	30.07
RS00470	RS00470	Gfo/Idh/MocA family oxidoreductase	16.11	6.67	14.63	19.19
RS00475	RS00475	Gfo/Idh/MocA family oxidoreductase	11.77	7	14.43	19.83
RS00480	RS00480	sugar O-acetyltransferase	23.59	16.89	24.16	39.77
RS00490	RS00490	alpha/beta fold hydrolase	110.96	116.48	255.14	158.97
RS00495	RS00495	diacylglycerol kinase family lipid kinase	158.83	132.14	264.31	187.19
RS00500	RS00500	alpha/beta hydrolase	25.87	8.55	25.93	19.9
RS00505	RS00505	thioredoxin family protein	177.09	96.15	178.7	157.73
RS00510	RS00510	EAL domain-containing protein	37.33	58.97	73.27	93.27
RS00515	RS00515	hypothetical protein	91.38	85.6	143.93	269.55
RS00520	RS00520	glycosyltransferase	36.77	25.44	54.74	82.27
RS00525	RS00525	6-phospho-beta-glucosidase	264.8	207.94	294.76	281.7
RS00530	RS00530	PHP domain-containing protein	945.82	1488.05	706.41	776.89
RS00550	RS00550	ArgE/DapE family deacylase	642.11	47.79	141.21	65.91

RS00555	RS00555	aldo/keto reductase	699.88	241.34	309.25	188.28
RS00560	RS00560	galactokinase	764.24	259.81	373.58	170.34
RS00565	RS00565	sigma-70 family RNA polymerase sigma factor	64.82	19.52	51.73	30.64
RS00570	RS00570	KxYKxGKxW signal peptide domain-containing protein	1626.66	2408.65	1462.9	1357.41
RS00585	RS00585	AraC family transcriptional regulator	3733.22	4154.58	2804.51	9498.99
RS00590	RS00590	hypothetical protein	238.04	359.04	427.19	246.81
RS00595	RS00595	hypothetical protein	13.05	12.05	34.12	40.97
RS00600	RS00600	LytTR family transcriptional regulator	6.62	5.16	11.83	10
RS00605	RS00605	MucBP domain-containing protein	13.29	36.39	172.11	105.42
RS00610	RS00610	hypothetical protein	19.68	14.31	26.04	25.7
RS00615	RS00615	serine hydrolase	13.46	5.25	11.97	13.93
RS00620	RS00620	glycosyltransferase family 2 protein	17.98	5.99	15.84	19.71
RS00625	RS00625	Ig-like domain-containing protein	11.56	5.47	9.98	11.9
RS00630	RS00630	MFS transporter	16.2	18.96	60.99	116.35
**RS00635**	**RS00635**	**aspartate-semialdehyde dehydrogenase**	**69.33**	**154.84**	**104.1**	**1481.78**
RS00640	RS00640	aminotransferase class I/II-fold pyridoxal phosphate-dependent enzyme	116.42	378.41	355.79	2632.13
**RS00645**	**dapB**	**4-hydroxy-tetrahydrodipicolinate reductase**	**66.72**	**253.25**	**277.36**	**1278.22**
**RS00650**	**dapA**	**4-hydroxy-tetrahydrodipicolinate synthase**	**101.74**	**538.93**	**674.25**	**2039.01**
RS00655	RS00655	N-acetyldiaminopimelate deacetylase	94.6	695.39	1049.6	2194.28
**RS00660**	**dapD**	**2%2C3%2C4%2C5-tetrahydropyridine-2%2C6-dicarboxylate N-acetyltransferase**	**52.22**	**400.29**	**690.36**	**909.61**
**RS00665**	**lysA**	**diaminopimelate decarboxylase**	**29.13**	**374.36**	**770.14**	**785.22**
**RS00670**	**RS00670**	**aspartate kinase**	**158.16**	**39.05**	**84.82**	**85.89**
RS00675	dapF	diaminopimelate epimerase	468.62	76.46	138.07	275.93
RS00680	RS00680	MFS transporter	210.89	34.32	83.26	112.66
RS00685	RS00685	MurR/RpiR family transcriptional regulator	24.82	115.53	84.78	37.38
RS00690	pfkB	1-phosphofructokinase	14.16	6.73	12.39	29.26
RS00695	RS00695	fructose-specific PTS transporter subunit EIIC	12.17	5.1	10.7	14.9
RS00700	lacD	tagatose-bisphosphate aldolase	26.41	9.24	20.66	19.99
RS00705	RS00705	fructose-1%2C6-bisphosphatase	82.49	239.34	196.57	111.98
RS00710	RS00710	magnesium transporter	43.94	87.83	85.21	86.02
RS00715	RS00715	SDR family oxidoreductase	1157.83	4054.61	1756.55	2537.42
RS00720	RS00720	hypothetical protein	253.8	126.91	187.38	95.08
RS00725	RS00725	hypothetical protein	5.96	3.03	6.32	7.51
RS00730	RS00730	MgtC/SapB family protein	333.18	95.45	149.75	177.53
RS00735	RS00735	MurR/RpiR family transcriptional regulator	54.86	28.5	47.8	56.69
RS00740	RS00740	N-acetylmannosamine-6-phosphate 2-epimerase	713.32	402.18	332.27	355.08
RS00745	RS00745	NAD(P)/FAD-dependent oxidoreductase	2046.05	1283.7	883.85	827.23
RS00765	RS00765	GntR family transcriptional regulator	227.07	53.95	69.51	65.5
RS00790	RS00790	sugar porter family MFS transporter	11.21	4.27	14.78	17.57
RS00795	RS00795	FGGY-family carbohydrate kinase	9.47	3.36	10.47	14.65
RS00800	RS00800	L-ribulose-5-phosphate 4-epimerase	12.25	4.44	12.3	16.17
RS00805	araA	L-arabinose isomerase	13.37	5.76	14.82	20.46
**RS00810**	**ald**	**alanine dehydrogenase**	**92.06**	**118.49**	**48.88**	**40.33**
RS00815	tdcB	bifunctional threonine ammonia-lyase/L-serine ammonia-lyase TdcB	88.11	62.6	58.12	49.27
RS00820	RS00820	OPT/YSL family transporter	73.5	23.3	61.96	43.39
RS00825	RS00825	PqqD family peptide modification chaperone	94.63	27.48	74.32	44.93

RS00830	RS00830	C69 family dipeptidase	144.93	33.46	91.77	46.87
**RS00840**	**agaC**	**PTS galactosamine transporter subunit IIC**	**105.4**	**36.27**	**95.51**	**89.87**
**RS00845**	**agaD**	**PTS galactosamine transporter subunit IID**	**207.62**	**22.59**	**130.09**	**87.69**
**RS00850**	**RS00850**	**PTS sugar transporter**	**794.74**	**81.32**	**545.34**	**310.2**
RS00995	RS00995	LacI family DNA-binding transcriptional regulator	87.67	74.46	208.06	94.94
RS01000	RS01000	galactose mutarotase	310.21	93.03	148.74	34.26
RS01005	RS01005	PTS sugar transporter subunit IIA	78.44	7.28	25.81	16.85
RS01010	RS01010	beta-galactosidase small subunit	34.49	15.64	54.44	22.16
RS01015	RS01015	beta-galactosidase	36.92	10.8	57.08	32.6
RS01020	RS01020	galactokinase	25.05	5.36	20.64	16.12
RS01025	galE	UDP-glucose 4-epimerase GalE	32.77	12.59	56.22	50.13
RS01030	RS01030	UDP-glucose--hexose-1-phosphate uridylyltransferase	34.33	11.9	51.13	55.49
RS01035	RS01035	class II fumarate hydratase	56.47	28.19	54.74	47.87
RS01040	RS01040	anion permease	0.01	0.01	0.03	0.03
RS01045	RS01045	peptidylprolyl isomerase	79.52	115.42	252.97	177.85
RS01050	RS01050	patatin family protein	501.16	1177.81	1697.19	430.64
RS01055	RS01055	MFS transporter	167.55	427.55	657.03	105.91
RS01060	RS01060	transcription repressor NadR	122.53	66.49	67.56	29.32
RS01065	RS01065	glycosyltransferase family 8 protein	2530.46	3591.21	1183.24	792.38
RS01070	RS01070	hypothetical protein	12248.87	21097.22	7191.71	4943.59
RS01075	RS01075	L-lactate dehydrogenase	402.18	390.67	470.88	647.16
RS01080	RS01080	YxeA family protein	27.55	130.34	202.65	199.15
RS01085	RS01085	YdcF family protein	44.96	280.99	427.42	389.73
RS01090	RS01090	galactose mutarotase	393.69	463.1	206.47	150.85
RS01095	rpsN	30S ribosomal protein S14	1569.63	391.5	1098.19	368.52
RS01100	RS01100	DMT family transporter	18.21	3.82	16.77	7.47
RS01105	RS01105	glycosyltransferase	64.95	73.13	90.63	79.35
RS01110	RS01110	glycosyltransferase	104.66	104.27	144.11	142.12
RS01115	RS01115	nitroreductase family protein	297.95	121.37	178.84	72.65
RS01120	RS01120	hypothetical protein	26.65	7.21	16.13	17.73
RS01125	thiD	bifunctional hydroxymethylpyrimidine kinase/phosphomethylpyrimidine kinase	265.88	265.84	544.82	149.39
RS01130	RS01130	DUF2974 domain-containing protein	20.32	14.05	34.64	18.82
RS01135	RS01135	MupG family TIM beta-alpha barrel fold protein	270.59	86.74	140.99	80.69
**RS01140**	**celB**	**PTS cellobiose transporter subunit IIC**	**651.92**	**211.5**	**255.79**	**236.19**
RS01145	RS01145	hypothetical protein	191.29	58.22	113.45	80.33
RS01150	RS01150	SDR family oxidoreductase	497.59	116.18	184.44	117.19
RS01155	pepV	dipeptidase PepV	52.48	256.16	214.8	146.58
RS01160	tyrS	tyrosine--tRNA ligase	24.57	22.49	52.15	53.84
RS01185	RS01185	cytosine permease	30.06	42.11	101.53	36.09
RS01190	codA	cytosine deaminase	103.19	119.16	163.89	136.24
**RS01195**	**RS01195**	**ABC transporter permease/substrate-binding protein**	**80.66**	**216.04**	**76.62**	**66.25**
RS01200	RS01200	ABC transporter ATP-binding protein	68.29	70.9	42.95	36.7
RS01205	RS01205	NAD(P)/FAD-dependent oxidoreductase	157.75	151.46	102.74	149.61
RS01210	RS01210	cation:proton antiporter	216.09	154.31	163.37	147.03
RS01215	RS01215	pyruvate carboxylase	225.26	500.08	392.87	385.62
RS01220	RS01220	pyridoxal phosphate-dependent aminotransferase	385.19	626.84	575.76	707.27
RS01250	RS01250	NADP-dependent oxidoreductase	158.67	348.34	333.38	252.63
RS01255	RS01255	DMT family transporter	43.87	48.7	68.35	67.08

**RS01260**	**RS01260**	**AAA family ATPase**	**34.66**	**370.08**	**485.3**	**688.89**
RS01265	trpS	tryptophan--tRNA ligase	127.44	80.7	176.63	157.01
RS01270	RS01270	Ppx/GppA family phosphatase	42.95	15.75	33.69	20.08
RS01275	RS01275	hydroxymethylglutaryl-CoA reductase%2C degradative	187.53	66.09	146.94	99.01
RS01280	RS01280	IpaB/EvcA family protein	145.54	45.03	91.15	62.73
RS01295	RS01295	PspC domain-containing protein	1389.41	1233.63	705.9	723.64
RS01300	RS01300	hypothetical protein	17.54	21.76	42.91	17.99
RS01305	RS01305	DUF72 domain-containing protein	66.45	103.39	164.62	211.26
RS01310	metG	methionine--tRNA ligase	146.22	178.24	237.03	315.54
RS01315	RS01315	TatD family hydrolase	83.36	93.18	128.26	199.21
RS01320	rnmV	ribonuclease M5	105.86	110.11	146.88	243.06
RS01325	rsmA	16S rRNA (adenine(1518)-N(6)/adenine(1519)-N(6))-dimethyltransferase RsmA	78.49	76.82	110.68	169.93
RS01330	RS01330	Veg family protein	783.71	1595.35	1206.83	594.91
RS01335	ispE	4-(cytidine 5'-diphospho)-2-C-methyl-D-erythritol kinase	76.05	161.79	143.09	93.74
RS01340	RS01340	TetR/AcrR family transcriptional regulator	38.93	39.16	52.8	35.08
RS01345	RS01345	universal stress protein	302.05	344.35	158.67	116.59
RS01350	RS01350	universal stress protein	255.96	383.52	178.59	103.62
RS01355	RS01355	DHA2 family efflux MFS transporter permease subunit	6.47	21.23	86.91	70.8
RS01360	RS01360	iron-containing alcohol dehydrogenase	116.37	25.03	33.77	45.38
RS01365	RS01365	ATP-binding cassette domain-containing protein	28.91	168.64	353.92	471.12
RS01370	RS01370	branched-chain amino acid ABC transporter permease	16.21	56.1	202.27	164.39
RS01375	RS01375	ABC transporter substrate-binding protein	17.08	48.42	304.47	153.02
RS01380	RS01380	GntR family transcriptional regulator	112.01	51.32	172.4	82.32
**RS01385**	**RS01385**	**PTS sugar transporter subunit IIB**	**1367.54**	**397.77**	**828.58**	**414.37**
**RS01390**	**RS01390**	**PTS lactose/cellobiose transporter subunit IIA**	**651.43**	**117.06**	**337.12**	**104.32**
RS01395	RS01395	6-phospho-beta-glucosidase	743.16	128.96	342.75	97.24
RS01400	RS01400	MurR/RpiR family transcriptional regulator	1079.44	198.64	471.83	150.38
RS01405	RS01405	FAD-dependent oxidoreductase	228.21	107.92	116.67	96.97
RS01410	RS01410	(S)-acetoin forming diacetyl reductase	119.52	33.34	48.68	39.84
RS01415	RS01415	uracil-DNA glycosylase family protein	89.34	27.52	76.4	32.92
RS01420	RS01420	GNAT family N-acetyltransferase	123.25	96.96	114.31	55.83
RS01425	RS01425	zinc ABC transporter substrate-binding protein	29.96	55.09	46.67	27.66
RS01430	RS01430	ATP-binding cassette domain-containing protein	27.54	48.7	45.59	29.94
RS01435	RS01435	metal ABC transporter permease	19.85	27	26.59	24.79
RS01440	purR	pur operon repressor	340.31	51.47	93.75	39.56
RS01445	glmU	bifunctional UDP-N-acetylglucosamine diphosphorylase/glucosamine-1-phosphate N-acetyltransferase GlmU	267.69	68.58	74.28	57.67
RS01450	RS01450	TIGR00730 family Rossman fold protein	12.05	15.94	35.79	53.59
RS01455	RS01455	ribose-phosphate diphosphokinase	180.93	127.38	212.17	140.11
RS01460	RS01460	zinc ribbon domain-containing protein	55.33	35.09	53.79	91.34
RS01465	RS01465	hypothetical protein	1267.63	2167.83	831.38	1215.57
RS01470	RS01470	hypothetical protein	1836.72	332.09	414.72	266.95
RS01475	RS01475	aquaporin	170.6	446.55	608.89	815.14
RS01480	RS01480	potassium channel family protein	19.99	24.34	61.62	41.3
**RS01485**	**RS01485**	**asparaginase**	**129.81**	**56.41**	**119.99**	**43.72**
RS01490	RS01490	peptidase M13	639.56	327.1	483.09	285.49
RS01495	RS01495	NCS2 family permease	24.64	60.07	32.65	43.12

RS01500	RS01500	ribonuclease H family protein	36.33	67.19	67.33	113.44
RS01505	RS01505	GNAT family N-acetyltransferase	216.42	300.65	280.4	176.61
RS01510	RS01510	LBP_cg2779 family protein	1803.95	1128.84	1551.24	842.95
RS01515	RS01515	LysR family transcriptional regulator	71.78	42.55	47.47	50.57
RS01520	RS01520	putative sulfate exporter family transporter	18.05	15.55	38.87	41.95
RS01525	RS01525	AEC family transporter	82.12	40.15	67.93	46.42
RS01530	RS01530	hypothetical protein	357.4	263.41	275.29	168.52
RS01535	RS01535	Cof-type HAD-IIB family hydrolase	449.27	158.61	253.58	110.74
RS01545	RS01545	aspartate carbamoyltransferase catalytic subunit	1064.27	184.48	341.41	48.85
RS01550	RS01550	dihydroorotase	1058.44	159.95	313.27	34.09
RS01555	RS01555	carbamoyl phosphate synthase small subunit	2470.7	1030	534.28	180.09
RS01560	carB	carbamoyl-phosphate synthase large subunit	1618.42	673.79	200.64	95.45
RS01565	RS01565	dihydroorotate dehydrogenase	961.82	333.32	67.59	46.74
**RS01570**	**RS01570**	**flavocytochrome c**	**1479.3**	**274.92**	**78.26**	**50.23**
RS01575	RS01575	hypothetical protein	27.16	9.86	47.92	17.17
RS01580	RS01580	hypothetical protein	18.68	5.87	24.84	19.38
RS01585	RS01585	hypothetical protein	23.87	11.38	38.35	29.87
RS01590	RS01590	glycosyltransferase family 2 protein	26.09	14.31	40.49	41.7
RS01595	RS01595	cellulose biosynthesis cyclic di-GMP-binding regulatory protein BcsB	27.96	18.48	46.67	56.8
RS01600	RS01600	GGDEF domain-containing protein	32.5	26.14	47.72	64.45
RS01605	RS01605	iron-sulfur cluster biosynthesis family protein	5147.53	12737.46	6849.28	8513.44
RS01610	RS01610	VOC family protein	117.46	105.05	107.65	134.7
RS01615	RS01615	TetR/AcrR family transcriptional regulator	7.97	59.25	33.81	99.2
RS01620	RS01620	MMPL family transporter	9.64	65.5	28.05	213.61
RS01625	RS01625	NAD(P)-dependent oxidoreductase	154.46	448.92	297.09	373.62
RS01630	RS01630	hypothetical protein	804.13	245.17	376.56	240.36
RS01635	RS01635	ABC transporter ATP-binding protein	955.55	165.05	576.68	352.98
RS01640	RS01640	ABC transporter substrate-binding protein	667.86	118.92	467.96	219.98
RS01645	RS01645	ABC transporter permease	486.12	91.67	367.96	167.11
RS01650	RS01650	thiamine-binding protein	1695.24	442.36	1698.77	913.9
RS01655	nagB	glucosamine-6-phosphate deaminase	52.08	23.73	53.26	27.41
**RS01660**	**RS01660**	**acetate kinase**	**572.27**	**224.55**	**537.15**	**273.55**
RS01665	pta	phosphate acetyltransferase	582.26	255.88	413.42	313.62
RS01670	RS01670	transcriptional repressor	39.22	20.34	43.11	24.01
RS01675	RS01675	MFS transporter	13.89	29.31	41.74	26.23
RS01680	RS01680	YihY/virulence factor BrkB family protein	16.6	30.87	57.41	37.78
RS01685	RS01685	ASCH domain-containing protein	264.04	172.86	205.69	175.34
RS01690	RS01690	tyrosine-protein phosphatase	175.38	42.06	76.68	76.61
RS01695	RS01695	tyrosine-protein phosphatase	163.51	183.55	177.5	259.24
RS01700	nrdI	class Ib ribonucleoside-diphosphate reductase assembly flavoprotein NrdI	205.31	167.05	197.47	119.28
RS01705	RS01705	Cof-type HAD-IIB family hydrolase	147.35	51.67	67.9	57.63
RS01710	RS01710	hemolysin family protein	159.78	96.65	106.75	68.37
RS01715	RS01715	aldo/keto reductase	677.22	471.64	415.33	351.98
RS01720	RS01720	GyrI-like domain-containing protein	58.8	100.62	201.61	59.93
RS01725	RS01725	hypothetical protein	20.84	29.45	77.96	23.03
RS01730	RS01730	DUF1836 domain-containing protein	317.43	217.22	103.61	111.27
RS01735	ybaK	Cys-tRNA(Pro) deacylase	88.26	57.24	132.51	86.25

RS01740	RS01740	GntR family transcriptional regulator	34.73	55.16	97.38	66.44
RS01745	RS01745	phosphoketolase family protein	149.38	282.38	130.5	126.05
RS01750	RS01750	RluA family pseudouridine synthase	55.92	20.44	62.55	53.62
RS01755	RS01755	universal stress protein	3080.35	1509.85	814.33	565.99
RS01760	RS01760	NAD(P)-dependent oxidoreductase	94.9	21.62	56.24	46.41
RS01765	RS01765	hypothetical protein	73.74	92.76	105.53	80.37
RS01770	RS01770	L%2CD-transpeptidase	355.05	163.25	197.67	154.21
RS01780	RS01780	VTT domain-containing protein	144.93	319.89	161.4	194.9
RS01785	RS01785	hypothetical protein	502.72	156.67	179.29	224
RS01790	RS01790	helix-turn-helix domain-containing protein	198.54	51.79	58.76	45.37
RS01795	RS01795	alpha/beta hydrolase	53.56	5.78	32.32	13.87
RS01800	RS01800	cation:proton antiporter	77.68	234.63	162.19	201.2
RS01805	RS01805	nucleoside hydrolase	208.61	67.55	176.53	62.46
RS01810	RS01810	NAD(P)/FAD-dependent oxidoreductase	39.27	27.82	49.89	53.44
RS01815	RS01815	LTA synthase family protein	24.66	105.25	123.58	113.44
RS01820	RS01820	GNAT family N-acetyltransferase	186.1	174.13	198.83	246.22
RS01825	RS01825	YibE/F family protein	98.38	87.8	103	105.37
RS01830	RS01830	YibE/F family protein	26.61	18.15	30.06	29.33
RS01835	RS01835	magnesium transporter CorA family protein	56.17	42.72	55.03	53.04
RS01840	RS01840	TerC family protein	95.54	130.55	101.84	92.43
RS01845	trxA	thioredoxin	115.16	115.4	106.91	75.91
RS01850	RS01850	DegV family protein	137.79	83.98	101.17	79.52
RS01855	map	type I methionyl aminopeptidase	233.47	114.75	147.04	104.41
RS01860	RS01860	VOC family protein	78.47	23.44	81.99	28.9
RS01865	RS01865	ATP-binding cassette domain-containing protein	149.83	215.02	389.23	363.16
RS01870	RS01870	aldo/keto reductase	1978.97	613.68	735.54	352.95
RS01875	RS01875	proline-specific peptidase family protein	637.61	247.97	444.31	293.49
RS01880	RS01880	Xaa-Pro dipeptidyl-peptidase	114.06	76.6	144.98	187.82
RS01885	RS01885	NCS2 family permease	17.08	30.4	15.25	19.23
RS01890	RS01890	amino acid permease	21.66	44.82	123.79	103.45
RS01895	RS01895	bis(5'-nucleosyl)-tetraphosphatase	77.49	40.71	45.6	46.73
RS01900	RS01900	ATP-binding cassette domain-containing protein	21.03	42.11	75.3	95.81
RS01905	RS01905	transcriptional repressor	62.39	202.6	79.23	85.56
RS01910	RS01910	hypothetical protein	543.23	711.06	811.63	687.9
RS01915	RS01915	hypothetical protein	448.17	590.66	700.05	954.31
RS01920	RS01920	amino acid ABC transporter ATP-binding protein	490.55	911.51	504.39	469.83
RS01925	RS01925	transporter substrate-binding domain-containing protein	364.07	751.2	456.62	465.92
RS01930	RS01930	amino acid ABC transporter permease	788.23	902.93	649.56	701.29
RS01935	RS01935	amino acid ABC transporter permease	533.24	606.42	457.06	501.17
RS01940	RS01940	GNAT family N-acetyltransferase	49.55	22.67	46.4	40.65
RS01945	RS01945	MerR family transcriptional regulator	33.2	20.98	38.15	28.97
RS01950	hflX	GTPase HflX	523.67	613.23	345.64	332.88
**RS01955**	**RS01955**	**peptide ABC transporter substrate-binding protein**	**301.05**	**3712.78**	**1873.06**	**1505.63**
**RS01960**	**RS01960**	**ABC transporter permease**	**154.41**	**1137.76**	**829.13**	**839.45**
**RS01965**	**RS01965**	**ABC transporter permease**	**113.49**	**681.33**	**483.85**	**489.03**
**RS01970**	**RS01970**	**ABC transporter ATP-binding protein**	**388.17**	**2412.47**	**1747.11**	**2535.95**
**RS01975**	**RS01975**	**ATP-binding cassette domain-containing protein**	**198.25**	**1033.43**	**752.86**	**994.67**
RS01980	RS01980	histidine phosphatase family protein	7.18	7.89	11.32	15.56
RS01985	RS01985	APC family permease	10.72	11.04	20.78	21.04

RS01990	RS01990	histidine phosphatase family protein	17.72	22.98	55.1	36.73
**RS01995**	**RS01995**	**AAA family ATPase**	**90.01**	**388.33**	**238.85**	**262.54**
RS02000	alsS	acetolactate synthase AlsS	80.84	136.31	151.85	226.99
RS02005	budA	acetolactate decarboxylase	123.76	180.99	190.59	344
RS02010	coaA	type I pantothenate kinase	216.3	306.35	385.8	603.68
RS02015	RS02015	Crp/Fnr family transcriptional regulator	35.73	40.1	100.73	130.69
RS02020	RS02020	heavy-metal-associated domain-containing protein	12.81	7.66	17.11	36.53
RS02025	RS02025	DNA starvation/stationary phase protection protein	8.47	10.12	21.05	60.95
RS02030	RS02030	heavy metal translocating P-type ATPase	16.61	14.38	27.2	83.44
RS02035	guaA	glutamine-hydrolyzing GMP synthase	544.83	853.39	714.3	545.73
RS02040	RS02040	hypothetical protein	67.76	19.26	43.57	27.41
RS02045	RS02045	helix-turn-helix domain-containing protein	14.88	15.46	56.98	29.11
RS02050	RS02050	hypothetical protein	51.42	50.54	71.44	125.42
RS02055	RS02055	MFS transporter	12.72	8.44	31.65	24.99
RS02060	RS02060	M1 family metallopeptidase	120.58	135.38	268.42	193.71
RS02065	RS02065	hypothetical protein	896.39	239.37	228.39	129.97
RS02070	RS02070	CPBP family intramembrane metalloprotease	57.9	74.34	136	86.87
RS02075	groES	co-chaperone GroES	808.47	119.04	459.12	137.59
RS02080	groL	chaperonin GroEL	4330.94	730.99	2094.31	669.76
RS02085	RS02085	APC family permease	133.68	215.09	343.74	254.77
RS02090	RS02090	undecaprenyl/decaprenyl-phosphate alpha-N-acetylglucosaminyl 1-phosphate transferase	24.77	102.84	104.45	57.75
RS02095	RS02095	YigZ family protein	11.26	6.85	8.86	10.77
RS02100	RS02100	helicase-related protein	6.21	3.45	7.84	13.98
RS02110	raiA	ribosome-associated translation inhibitor RaiA	2455.39	1624.25	804.42	701.69
RS02115	secA	preprotein translocase subunit SecA	230.5	334.65	349.95	386.1
RS02120	prfB	peptide chain release factor 2	105.44	36.66	87.54	84.68
**RS02125**	**RS02125**	**response regulator transcription factor**	**102.78**	**27.08**	**60.74**	**53.9**
**RS02130**	**RS02130**	**ATP-binding protein**	**54.92**	**8.3**	**24.82**	**16.56**
**RS02135**	**RS02135**	**phosphate ABC transporter substrate-binding protein PstS family protein**	**8.99**	**573.11**	**87.97**	**269.16**
**RS02140**	**pstC**	**phosphate ABC transporter permease subunit PstC**	**6.58**	**633.78**	**91.79**	**167.27**
**RS02145**	**pstA**	**phosphate ABC transporter permease PstA**	**6.7**	**812.03**	**92.78**	**116.25**
**RS02150**	**pstB**	**phosphate ABC transporter ATP-binding protein PstB**	**12.89**	**1492.16**	**130.78**	**174.82**
**RS02155**	**pstB**	**phosphate ABC transporter ATP-binding protein PstB**	**9.32**	**1282.32**	**91.22**	**142.76**
RS02160	phoU	phosphate signaling complex protein PhoU	17.54	1117.57	75.1	149.88
RS02165	RS02165	PspC domain-containing protein	728.6	1118.42	787.46	866.51
RS02170	RS02170	phage holin family protein	51.81	53.38	49.6	43.46
RS02175	hprK	HPr(Ser) kinase/phosphatase	393.25	259.38	290.14	226.36
RS02180	Lgt	prolipoprotein diacylglyceryl transferase	191.03	127.74	160.47	125.91
RS02185	RS02185	NAD(P)H-dependent glycerol-3-phosphate dehydrogenase	608.58	265.35	430.44	299.27
RS02190	galU	UTP--glucose-1-phosphate uridylyltransferase GalU	902.97	386.76	564.87	382.86
RS02195	trxB	thioredoxin-disulfide reductase	164.52	369.47	340.08	408.74
**RS02200**	**RS02200**	**phospho-sugar mutase**	**382.04**	**107.12**	**146.85**	**73.28**
RS02205	RS02205	HD domain-containing protein	341.93	377.98	237.11	246.8
RS02210	uvrB	excinuclease ABC subunit UvrB	253.64	463.45	288.13	336.69
RS02215	uvrA	excinuclease ABC subunit UvrA	207.86	378.84	158.28	327.15
RS02220	rapZ	RNase adapter RapZ	150.98	223.79	111.5	193.4
RS02225	RS02225	YvcK family protein	245.58	260.53	124.24	221.98

RS02230	whiA	DNA-binding protein WhiA	246.05	289.02	126.35	274.42
RS02235	RS02235	glucosaminidase domain-containing protein	151.72	67.07	140.83	77.09
RS02240	RS02240	hypothetical protein	627.99	1841	1411.49	1538.78
RS02245	clpP	ATP-dependent Clp endopeptidase proteolytic subunit ClpP	1330.89	1418.06	1140.92	380.18
**RS02255**	**rpoN**	**RNA polymerase factor sigma-54**	**174.3**	**75.37**	**85.47**	**76.44**
RS02260	RS02260	SorC family transcriptional regulator	562.4	1002.92	912.17	556.67
**RS02265**	**gap**	**type I glyceraldehyde-3-phosphate dehydrogenase**	**7382.98**	**2748.41**	**4026.47**	**1874.37**
**RS02270**	**RS02270**	**phosphoglycerate kinase**	**1041.55**	**425.37**	**485.99**	**312.61**
RS02275	tpiA	triose-phosphate isomerase	1384.37	205.52	456.95	116.41
**RS02280**	**eno**	**phosphopyruvate hydratase**	**2738.11**	**654.81**	**1651.23**	**483.1**
RS02285	secG	preprotein translocase subunit SecG	142.44	396.03	281.37	94.18
RS02290	rnr	ribonuclease R	88.53	171.66	169.5	132.6
RS02295	smpB	SsrA-binding protein SmpB	112.78	314.05	239.81	288.86
RS02300	RS02300	GNAT family N-acetyltransferase	188.03	139.24	160.04	218.94
RS02305	RS02305	hypothetical protein	220.54	133.02	143.9	234.15
RS02310	RS02310	Cof-type HAD-IIB family hydrolase	248.39	106.87	106.64	131.54
RS02315	RS02315	uracil-DNA glycosylase	171.05	143.06	170.43	85.37
RS02320	tsaE	tRNA (adenosine(37)-N6)-threonylcarbamoyltransferase complex ATPase subunit type 1 TsaE	48.52	26.9	36.15	29.36
RS02325	RS02325	GNAT family N-acetyltransferase	97.39	65.4	90.54	77.1
RS02330	RS02330	3'-5' exonuclease	881.54	299.91	460.01	251.05
RS02335	RS02335	Gfo/Idh/MocA family oxidoreductase	76.49	51.09	66.82	69.28
RS02340	murB	UDP-N-acetylmuramate dehydrogenase	340.19	105.53	136.72	150.98
RS02345	cdaA	diadenylate cyclase CdaA	136.38	71.3	73.39	115.7
RS02350	RS02350	CdaR family protein	296.96	155.14	157.57	236.28
RS02355	glmM	phosphoglucosamine mutase	190.62	96.54	111.67	144.74
RS02360	glmS	glutamine--fructose-6-phosphate transaminase (isomerizing)	99.51	384.07	244.13	952.93
RS02365	RS02365	hypothetical protein	68.16	34.85	57.32	47.03
RS02375	RS02375	hypothetical protein	17.01	82.91	63.22	79.19
RS02380	RS02380	YjjG family noncanonical pyrimidine nucleotidase	22.68	18.67	34.56	35.53
RS02385	RS02385	divalent metal cation transporter	20.88	15.44	28.84	23.16
RS02390	nrdD	anaerobic ribonucleoside-triphosphate reductase	76.77	29.14	65.56	48.23
RS02395	nrdG	anaerobic ribonucleoside-triphosphate reductase activating protein	79.92	28.5	52.87	44.88
RS02400	sufC	Fe-S cluster assembly ATPase SufC	109.71	35.93	68.43	59.31
RS02405	RS02405	SufD family Fe-S cluster assembly protein	73.38	22.31	45.32	35.55
RS02410	RS02410	cysteine desulfurase	95.65	29.85	62.97	39.96
RS02415	RS02415	SUF system NifU family Fe-S cluster assembly protein	160.2	46.35	102.74	68.02
RS02420	sufB	Fe-S cluster assembly protein SufB	92.69	27.69	64.98	42.06
RS02425	RS02425	phenolic acid decarboxylase	7.69	12.61	12.39	14.6
RS02430	RS02430	PadR family transcriptional regulator	68.6	118.68	90.49	85.96
RS02435	RS02435	LapA family protein	20.58	20.6	30.51	19.82
RS02440	RS02440	hypothetical protein	13.85	10.68	15.8	11.46
RS02450	RS02450	5-methyltetrahydropteroyltriglutamate--homocysteine S-methyltransferase	0.17	0.06	0.2	0.5
RS02460	RS02460	IS30 family transposase	9.88	9.46	15.6	21.84
RS02465	RS02465	pyridoxamine 5'-phosphate oxidase family protein	249.85	238.89	199.04	252.48
RS02470	RS02470	hypothetical protein	68.04	14.76	14.96	16.7
RS02475	RS02475	multicopper oxidase domain-containing protein	114.69	15.65	20.4	17.27
RS02480	RS02480	MFS transporter	74.09	11.76	11.97	12.74

RS02490	RS02490	minor capsid protein	12.86	3.85	5.36	7.03
RS02495	RS02495	GlsB/YeaQ/YmgE family stress response membrane protein	733.54	300.85	221.75	195.52
RS02525	RS02525	IS30 family transposase	34.34	50.63	40.67	35.86
RS02565	RS02565	IS3 family transposase	189.43	292.3	87.6	39.87
RS02570	RS02570	IS3 family transposase	193.92	207.11	104.03	32.44
RS02655	RS02655	LicD family protein	56.46	38.17	79.33	42.83
RS02660	RS02660	TMEM175 family protein	50.45	28.16	70.34	36.91
RS02665	RS02665	SDR family oxidoreductase	25.33	47.38	82.81	111.28
RS02670	RS02670	MerR family transcriptional regulator	22.88	49.44	88.73	116.62
RS02675	asnS	asparagine--tRNA ligase	129.51	49.57	87.97	77.26
RS02700	RS02700	MarC family protein	66.34	153.57	73.64	106.43
RS02705	RS02705	hypothetical protein	46.64	33.74	46.75	64.17
RS02710	RS02710	DUF916 and DUF3324 domain-containing protein	17.95	5.04	18.74	27.84
RS02715	RS02715	CsbD family protein	92012.82	124736.56	64853.73	82778.11
RS02720	thiM	hydroxyethylthiazole kinase	49.73	8.44	34.38	14.15
RS02725	thiD	bifunctional hydroxymethylpyrimidine kinase/phosphomethylpyrimidine kinase	108.17	18.37	69.65	31.23
RS02730	thiE	thiamine phosphate synthase	57.41	11.84	33.54	19.41
RS02735	tenA	thiaminase II	88.92	15.68	56.25	30.64
RS02740	recQ	DNA helicase RecQ	33	26.67	49.11	48.56
RS02745	RS02745	matrixin family metalloprotease	77.31	76.76	90.01	143.9
RS02750	RS02750	serine hydrolase	13.03	12.84	32.94	14.21
RS02755	RS02755	Wzz/FepE/Etk N-terminal domain-containing protein	12.95	7.93	22.06	6.82
RS02760	RS02760	CpsD/CapB family tyrosine-protein kinase	21.39	10.31	32.75	16.35
RS02765	RS02765	tyrosine protein phosphatase	98.6	48.65	124.31	88.55
RS02800	Glf	UDP-galactopyranose mutase	2.33	1.33	2.43	1.1
RS02845	RS02845	VanZ family protein	18.07	25.23	36.3	33.16
RS02850	RS02850	amino acid permease	12.97	29.79	34.72	32.21
RS02855	RS02855	WYL domain-containing protein	5.23	4.08	10.94	15.58
RS02860	RS02860	NAD(P)H-dependent oxidoreductase	10.11	4.58	13.06	30.95
RS02865	RS02865	GH25 family lysozyme	159.31	83.75	79.53	123.84
RS02870	RS02870	DUF4828 domain-containing protein	882.3	1407.76	966.02	765.43
RS02875	RS02875	Gfo/Idh/MocA family oxidoreductase	304.75	237.33	126.62	123.64
RS02880	RS02880	DEAD/DEAH box helicase	171.39	122.54	122.16	98.93
RS02885	RS02885	LCP family protein	246.52	228.59	256.12	207.61
RS02890	RS02890	hypothetical protein	42.82	44.48	51.12	35.65
RS02895	RS02895	helix-turn-helix domain-containing protein	3881.14	3803.8	4251.44	2404.26
RS02905	RS02905	GMP reductase	85.54	205.49	30.38	59.71
RS02910	RS02910	aminopeptidase	101.34	405.07	331.96	286.65
RS02915	wecB	UDP-N-acetylglucosamine 2-epimerase (non-hydrolyzing)	273.11	63.23	58.36	51.33
RS02920	RS02920	flavodoxin	188.27	213.67	287.17	143.92
RS02925	recX	recombination regulator RecX	23.54	22.18	33.03	36.09
RS02930	RS02930	DUF402 domain-containing protein	297.11	301.14	328.68	261.09
RS02935	RS02935	AI-2E family transporter	101.8	58.71	91.65	47.74
RS02940	tagD	glycerol-3-phosphate cytidylyltransferase	524.69	194.18	497.16	267.84
RS02945	RS02945	peptide chain release factor 3	128.29	20.95	94.61	25.26
RS02950	RS02950	LTA synthase family protein	147.83	86.66	231.61	124
RS02955	RS02955	sulfite exporter TauE/SafE family protein	65.13	34.62	75.34	69.37
RS02960	RS02960	DUF1634 domain-containing protein	180.76	135.85	262.69	262.25

RS02965	RS02965	ATP-dependent Clp protease ATP-binding subunit	1603.16	1509.12	850.24	995.93
RS02970	RS02970	hypothetical protein	1578.21	1315.31	1750.9	1240.71
RS02975	RS02975	phosphocarrier protein HPr	6914.8	3169.62	5559.77	2410.98
RS02980	ptsP	phosphoenolpyruvate--protein phosphotransferase	3028.13	1200.26	2382.67	1189.61
RS02985	RS02985	alpha/beta hydrolase	87.83	18.9	51.83	40.59
RS02990	RS02990	glycosyltransferase family 4 protein	75.85	47.21	47.64	29.21
RS02995	RS02995	glycosyltransferase family 4 protein	129.92	97.24	150.53	129.33
RS03000	RS03000	flippase-like domain-containing protein	148.02	134.81	203.03	188.19
RS03125	spxB	pyruvate oxidase	22.22	13.55	15.76	20.08
RS03130	RS03130	hypothetical protein	15.71	8.05	25.56	9.45
RS03135	metK	methionine adenosyltransferase	43.03	86.07	105.17	124.7
RS03140	RS03140	MFS transporter	11.04	15.82	39.69	57.39
RS03145	RS03145	class I SAM-dependent methyltransferase	111.41	55.03	74.07	52.79
RS03150	RS03150	phosphatase PAP2 family protein	19.21	6.61	11.97	6.21
RS03155	leuS	leucine--tRNA ligase	43.47	39.23	68.89	105
RS03160	RS03160	polysaccharide biosynthesis protein	34.59	24.92	52.33	25.67
RS03165	RS03165	16S rRNA pseudouridine(516) synthase	67.87	37.98	66.3	66.55
RS03170	RS03170	NAD(P)H-hydrate dehydratase	558.07	199.48	162.42	158
RS03175	RS03175	universal stress protein	501	225.12	135.96	94.06
RS03180	RS03180	deoxynucleoside kinase	44.17	24.3	100.46	60.28
RS03185	RS03185	Cof-type HAD-IIB family hydrolase	67.93	56.79	87.67	57.06
RS03195	RS03195	hypothetical protein	492.13	871.24	1044.4	1743.61
RS03200	RS03200	hypothetical protein	112.26	173.83	217.73	208.91
RS03205	RS03205	hypothetical protein	66.53	36.26	36.29	27.67
RS03215	RS03215	carbonic anhydrase family protein	168.06	23.32	140.99	64.21
RS03220	RS03220	DUF308 domain-containing protein	109.45	526.36	161.19	380.93
RS03225	asnB	asparagine synthase (glutamine-hydrolyzing)	90.7	123.03	212.43	106.25
RS03230	RS03230	UDP-N-acetylmuramoyl-L-alanyl-D-glutamate--2%2C6-diaminopimelate ligase	114.66	184.81	199.06	168.33
RS03235	RS03235	carboxylate--amine ligase	110.05	177.93	182.3	198.53
RS03240	RS03240	amino acid racemase	93.05	184.9	179.78	175.24
RS03245	RS03245	DNA/RNA non-specific endonuclease	86.57	139.36	263.73	428.06
RS03250	RS03250	amino acid permease	24.91	11.33	12.78	12.32
RS03260	RS03260	hypothetical protein	14.06	3.39	3.68	5.43
RS03265	RS03265	Cof-type HAD-IIB family hydrolase	24.09	8.26	4.52	5.74
RS03270	RS03270	aldo/keto reductase	129.52	60.79	87.88	61.69
RS03275	argS	arginine--tRNA ligase	128.65	504.39	92.13	341.34
RS03280	RS03280	Crp/Fnr family transcriptional regulator	5.45	5.71	9.14	12.5
RS03285	RS03285	ArgR family transcriptional regulator	15.11	32.5	48.63	54.89
**RS03290**	**RS03290**	**PBP1A family penicillin-binding protein**	**29.65**	**143.58**	**127.67**	**211.83**
RS03295	RS03295	YlbF family regulator	306.18	328.78	206.53	221.83
RS03300	RS03300	DNA repair exonuclease	87.59	46.33	48.72	55.14
RS03305	RS03305	AAA family ATPase	78.61	33.09	46.85	41.19
RS03310	RS03310	HD domain-containing protein	65.45	38.01	50.69	71.85
RS03315	RS03315	peptidylprolyl isomerase	372.4	287.2	519.07	228.47
RS03320	RS03320	hypothetical protein	334.03	79.95	76.47	57.14
RS03325	RS03325	HIT family protein	415.61	109.17	75.1	50.14
RS03330	RS03330	ABC transporter ATP-binding protein	43.22	58.99	63.62	55.41
RS03335	RS03335	ABC transporter permease	28.6	19.74	29.41	23.87

RS03340	trmB	tRNA (guanosine(46)-N7)-methyltransferase TrmB	47.45	29.19	78.85	62.75
RS03345	RS03345	PepSY domain-containing protein	1227.56	223.04	156.39	103.32
RS03350	RS03350	DUF4479 and tRNA-binding domain-containing protein	34.54	20.41	91.99	98.25
RS03355	RS03355	DNA translocase FtsK	33.67	21.08	69.47	90.21
RS03360	murC	UDP-N-acetylmuramate--L-alanine ligase	73.27	54.87	83.44	76.88
RS03365	RS03365	Bax inhibitor-1/YccA family protein	990.95	659.93	503.1	312.01
RS03370	polA	DNA polymerase I	115.55	120.56	128.92	129.87
RS03375	mutM	bifunctional DNA-formamidopyrimidine glycosylase/DNA-(apurinic or apyrimidinic site) lyase	202.59	189.63	176.03	244.6
RS03380	coaE	dephospho-CoA kinase	68.93	55.7	55.61	72.14
RS03385	nrdR	transcriptional regulator NrdR	143.79	132.23	116.4	204.25
RS03390	RS03390	DnaD domain protein	186.34	156.3	146.5	274.29
RS03395	dnaI	primosomal protein DnaI	245.26	227.7	221.5	479.84
RS03400	thrS	threonine--tRNA ligase	174.11	28.32	68.81	49.49
RS03405	RS03405	histidine kinase	17.08	5.59	7.72	12.23
RS03410	RS03410	LytTR family transcriptional regulator DNA-binding domain-containing protein	5.76	1.05	2.26	3.45
RS03415	RS03415	CidA/LrgA family protein	18.18	26.21	30.13	28.07
RS03420	RS03420	LrgB family protein	18.36	18.37	23.2	25.2
RS03425	infC	translation initiation factor IF-3	191.26	260.8	481.28	156.02
RS03430	rpmI	50S ribosomal protein L35	1302.31	2695.6	3771.74	2959.56
RS03435	rplT	50S ribosomal protein L20	331.68	850.57	1063.62	631.57
RS03440	RS03440	NADP-dependent oxidoreductase	438.68	563.17	403.1	509.06
RS03445	RS03445	YqeG family HAD IIIA-type phosphatase	26.75	90.21	82.68	70.51
RS03450	yqeH	ribosome biogenesis GTPase YqeH	89.13	89.93	107.04	103.57
RS03455	yhbY	ribosome assembly RNA-binding protein YhbY	151.72	121.45	153.9	168.81
RS03460	RS03460	nicotinate-nucleotide adenylyltransferase	192.02	134.75	171.65	218.84
RS03465	yqeK	bis(5'-nucleosyl)-tetraphosphatase (symmetrical) YqeK	244.06	145.94	172.61	327.84
RS03470	rsfS	ribosome silencing factor	182.9	114.17	136.23	208.17
RS03475	RS03475	class I SAM-dependent methyltransferase	100.8	65.82	80.34	174.85
RS03480	RS03480	nucleotidyltransferase family protein	169.68	112.49	136.69	317.31
RS03485	RS03485	DUF177 domain-containing protein	369.23	1315.36	1662.95	1125.98
RS03490	rpmF	50S ribosomal protein L32	213.3	313.48	338.71	268
RS03495	gndA	NADP-dependent phosphogluconate dehydrogenase	271.92	281.34	409.44	266.68
RS03500	RS03500	response regulator transcription factor	126	123.38	139.74	109.76
RS03505	RS03505	HAMP domain-containing histidine kinase	105.71	44.48	47.61	44.88
**RS03510**	**yidC**	**membrane protein insertase YidC**	**73.59**	**142.12**	**188.97**	**237.17**
RS03515	RS03515	acylphosphatase	166.37	275.29	99.51	135.29
RS03520	RS03520	RNA methyltransferase	178.44	126.53	126.45	83.42
RS03525	RS03525	hypothetical protein	348.66	158.51	155.8	179.42
RS03530	RS03530	helix-turn-helix transcriptional regulator	654.46	320.99	358.82	358.41
RS03535	pheS	phenylalanine--tRNA ligase subunit alpha	212.08	54.85	129.85	146.43
RS03540	pheT	phenylalanine--tRNA ligase subunit beta	153.77	46.22	97.38	114.48
RS03545	mltG	endolytic transglycosylase MltG	108.76	89.21	141.8	127.72
RS03550	udk	uridine kinase	290.51	274.42	420.77	478.23
RS03555	greA	transcription elongation factor GreA	125.15	112.03	166.9	225.93
RS03560	RS03560	YfhO family protein	6.01	39.28	35.73	33.63
RS03565	RS03565	penicillin-binding protein 2	108.22	125.17	146.83	136.57
RS03570	RS03570	5-formyltetrahydrofolate cyclo-ligase	63.27	57.07	146.25	173.05

RS03575	RS03575	rhomboid family intramembrane serine protease	41.33	31.82	78.39	80.93
RS03580	RS03580	YqgQ family protein	174.29	368.38	1138.56	581.45
RS03585	RS03585	ROK family glucokinase	119.27	109.23	302.82	216.29
RS03590	RS03590	rhodanese-like domain-containing protein	51.27	42.1	96.34	80.65
RS03595	RS03595	glycerophosphodiester phosphodiesterase	31.95	31.9	62.24	45.71
RS03600	miaA	tRNA (adenosine(37)-N6)-dimethylallyltransferase MiaA	49.61	42.65	78.77	76.08
RS03605	RS03605	methionine gamma-lyase family protein	49.31	35.52	68.9	77.14
RS03610	RS03610	MerR family transcriptional regulator	518.97	69.88	79.79	74.13
**RS03615**	**glnA**	**type I glutamate--ammonia ligase**	**1070.97**	**122.07**	**136.73**	**175.19**
RS03620	RS03620	hypothetical protein	332.82	141.63	153.71	71.93
RS03625	RS03625	hypothetical protein	170.06	54.85	72.28	29.59
RS03630	RS03630	dUTP diphosphatase	83.22	22.81	32.91	14.91
RS03635	RS03635	hypothetical protein	229.97	296.29	148.55	164.85
RS03640	RS03640	hypothetical protein	2.23	6.14	9.49	11.42
RS03675	RS03675	phage regulatory protein	0	0.04	0	0.02
RS03695	RS03695	single-stranded DNA-binding protein	3.46	1.06	2.19	2.2
RS03735	RS03735	hypothetical protein	2.8	1	1.44	1.9
RS03745	RS03745	hypothetical protein	2.52	1.47	2.84	3.97
RS03775	RS03775	DNA-directed RNA polymerase sigma-70 factor	8.35	4.32	7.72	11.24
RS03915	RS03915	GH25 family lysozyme	0	0	0.01	0
RS03925	RS03925	hypothetical protein	382.9	191.38	97.84	162.18
RS03930	RS03930	TetR/AcrR family transcriptional regulator	12.99	92.52	68.83	116.55
RS03935	rplU	50S ribosomal protein L21	528.61	1059.97	1863.98	739.79
RS03940	RS03940	ribosomal-processing cysteine protease Prp	195.12	359.52	577.27	323.83
RS03945	rpmA	50S ribosomal protein L27	720.59	1071.85	1659.99	943.05
RS03950	efp	elongation factor P	89.62	174.22	249.02	197.54
RS03955	RS03955	Asp23/Gls24 family envelope stress response protein	85.82	92.21	145.12	120.16
RS03960	nusB	transcription antitermination factor NusB	111.12	123.95	200.25	159.88
RS03965	folD	bifunctional methylenetetrahydrofolate dehydrogenase/methenyltetrahydrofolate cyclohydrolase FolD	28.91	71.22	123.06	88.91
RS03970	xseA	exodeoxyribonuclease VII large subunit	41.28	92.93	128.78	88.96
**RS03975**	**RS03975**	**exodeoxyribonuclease VII small subunit**	**109.39**	**286.55**	**357.58**	**289.21**
RS03980	RS03980	polyprenyl synthetase family protein	43.59	51.44	79.83	45.08
RS03985	RS03985	TlyA family RNA methyltransferase	87.7	49.1	134.37	99.88
RS03990	RS03990	arginine repressor%2C DNA-binding domain protein	31.05	19.68	45.88	29.19
RS03995	recN	DNA repair protein RecN	100.03	87.26	152.01	187.39
RS04000	RS04000	hypothetical protein	233.87	283.92	299.95	300.56
RS04005	gmk	guanylate kinase	141.27	104.82	129.92	63.62
RS04010	rpoZ	DNA-directed RNA polymerase subunit omega	353.99	207.16	222.16	123.93
RS04015	coaBC	bifunctional phosphopantothenoylcysteine decarboxylase/phosphopantothenate--cysteine ligase CoaBC	70.35	20.43	38.46	19.52
RS04020	priA	primosomal protein N'	79.86	33.43	48.66	44.25
RS04025	fmt	methionyl-tRNA formyltransferase	240.99	100.55	143.19	169.25
RS04030	rsmB	16S rRNA (cytosine(967)-C(5))-methyltransferase RsmB	159.52	86.5	105.48	152.11
RS04035	RS04035	Stp1/IreP family PP2C-type Ser/Thr phosphatase	141.56	50.72	78.54	76.25
RS04040	pknB	Stk1 family PASTA domain-containing Ser/Thr kinase	139.15	54.29	79.63	76.5
RS04045	rsgA	ribosome small subunit-dependent GTPase A	271.47	75.58	141.88	122.32
RS04050	rpe	ribulose-phosphate 3-epimerase	196.73	36.74	84.92	56.55
RS04055	RS04055	thiamine diphosphokinase	64.27	11.99	27.41	15.95

RS04060	rpmB	50S ribosomal protein L28	1026.08	3081.39	2828.39	2507.06
RS04065	RS04065	Asp23/Gls24 family envelope stress response protein	370.66	158.64	202.4	183.79
RS04070	RS04070	DAK2 domain-containing protein	291.51	101.91	159.43	142.48
RS04075	recG	ATP-dependent DNA helicase RecG	61.91	30.07	49.91	56.79
RS04080	plsX	phosphate acyltransferase PlsX	160.23	105.02	135.18	154.03
RS04085	acpP	acyl carrier protein	12.04	14.72	23.28	13.3
RS04090	rnc	ribonuclease III	37.43	33.85	46.92	38.1
RS04095	smc	chromosome segregation protein SMC	68.79	71.44	84.83	75.14
RS04100	ftsY	signal recognition particle-docking protein FtsY	132.17	117.57	128.59	186.94
RS04105	RS04105	putative DNA-binding protein	167.26	154.91	160.05	290.2
RS04110	Ffh	signal recognition particle protein	85.78	61.13	75.02	99.75
RS04115	rpsP	30S ribosomal protein S16	354.46	1320.81	1326.86	574.67
RS04120	rimM	ribosome maturation factor RimM	12.33	28.99	46.8	19.84
RS04125	trmD	tRNA (guanosine(37)-N1)-methyltransferase TrmD	25.66	58.07	86.91	42.13
RS04130	rplS	50S ribosomal protein L19	747.98	1327.05	1954.75	923.41
RS04140	RS04140	DsrE family protein	15.97	83.96	52.52	70.33
RS04145	fabZ	3-hydroxyacyl-ACP dehydratase FabZ	62.6	31.04	123.58	34.29
RS04150	RS04150	ketoacyl-ACP synthase III	44.28	21.37	75.11	28.37
RS04155	acpP	acyl carrier protein	495	242.38	588.67	316.63
RS04160	RS04160	ACP S-malonyltransferase	108.73	55.41	152.72	67.09
RS04165	fabG	3-oxoacyl-ACP reductase FabG	84.52	45	119.43	68.61
RS04170	fabF	beta-ketoacyl-ACP synthase II	43.84	30.8	66.56	38.29
RS04175	RS04175	acetyl-CoA carboxylase biotin carboxyl carrier protein subunit	82.83	46.1	116.06	65.36
RS04180	RS04180	beta-hydroxyacyl-ACP dehydratase	87.32	42.45	118.34	63.38
RS04185	RS04185	acetyl-CoA carboxylase biotin carboxylase subunit	69.48	32.01	103.47	51.61
RS04190	RS04190	acetyl-CoA carboxylase carboxyltransferase subunit beta	139.26	50.2	180.99	90.75
RS04195	RS04195	hypothetical protein	136.22	31.38	150.15	69.87
RS04200	fabI	enoyl-ACP reductase FabI	138.45	27.09	147.56	58.35
RS04205	RS04205	amino acid permease	14.26	22.31	57.03	91.1
RS04210	RS04210	hydroxymethylglutaryl-CoA synthase	102.1	263.95	285.9	388.14
RS04215	RS04215	hypothetical protein	141.51	53.97	38.7	15.43
RS04220	lexA	transcriptional repressor LexA	68.46	223.59	86.13	49.39
RS04225	RS04225	DUF896 domain-containing protein	242.4	371.79	172.08	182.81
RS04230	RS04230	YneF family protein	99.51	103.2	121.54	92.68
RS04235	RS04235	1-acyl-sn-glycerol-3-phosphate acyltransferase	73.95	32.25	57.35	31.51
RS04240	RS04240	tRNA1(Val) (adenine(37)-N6)-methyltransferase	9.27	2.6	10.25	7.55
RS04245	RS04245	GIY-YIG nuclease family protein	5.95	3.79	7.78	10.88
RS04250	RS04250	D-2-hydroxyacid dehydrogenase	166	207.81	232.83	189.83
RS04255	rpsB	30S ribosomal protein S2	1133.06	1510.85	1870.36	1675.98
RS04260	Tsf	translation elongation factor Ts	268.7	532.31	509.86	604.79
RS04265	pyrH	UMP kinase	445.25	322.42	312.13	334.01
RS04270	Frr	ribosome recycling factor	538.97	316.1	311.6	314.26
RS04275	RS04275	isoprenyl transferase	47	97.21	105.13	135.89
RS04280	RS04280	phosphatidate cytidylyltransferase	31.6	49	59.84	75.81
RS04285	rseP	RIP metalloprotease RseP	57.84	90.38	109.61	194.16
RS04290	RS04290	proline--tRNA ligase	93.5	145.98	137.35	287.48
**RS04295**	**RS04295**	**PolC-type DNA polymerase III**	**42.01**	**60.64**	**80.61**	**216.28**
RS04300	rimP	ribosome maturation factor RimP	137.32	275.93	372.3	336.13
RS04305	nusA	transcription termination factor NusA	122	250.22	306.04	364.51

RS04310	RS04310	YlxR family protein	71.51	194.36	203.87	277.01
RS04315	RS04315	ribosomal L7Ae/L30e/S12e/Gadd45 family protein	132.1	394.58	362.7	684.69
RS04320	infB	translation initiation factor IF-2	244.12	723.95	573.04	1395.45
RS04325	rbfA	30S ribosome-binding factor RbfA	211.23	516.7	357.82	1430.62
RS04330	truB	tRNA pseudouridine(55) synthase TruB	152.28	40.09	76.54	77.73
RS04335	ribF	riboflavin biosynthesis protein RibF	77.64	40.72	60.24	71.62
RS04340	hrcA	heat-inducible transcriptional repressor HrcA	277.2	214.22	261.96	140.37
RS04345	grpE	nucleotide exchange factor GrpE	665.33	421.58	547.77	304.93
RS04350	dnaK	molecular chaperone DnaK	979.33	843.14	890.08	580.73
RS04355	dnaJ	molecular chaperone DnaJ	217.05	360.02	308.14	325.98
RS04360	RS04360	DUF378 domain-containing protein	325.7	735.3	359.8	510.11
RS04365	RS04365	tryptophan-rich sensory protein	110.92	313.48	122.57	186.37
RS04370	RS04370	QueT transporter family protein	5.2	2.17	5.52	3.99
RS04375	RS04375	DUF2829 domain-containing protein	7.2	2.2	5	9.74
RS04380	RS04380	3-oxoacyl-ACP reductase	27.17	11.45	27.54	19.9
RS04385	RS04385	DUF4373 domain-containing protein	7.54	3.25	7.26	10.18
RS04390	RS04390	GlsB/YeaQ/YmgE family stress response membrane protein	187.06	712.14	616.59	660.9
RS04395	RS04395	manganese catalase family protein	8.07	20.52	25.8	68.75
RS04400	lepA	translation elongation factor 4	144.07	56.54	73.19	53.43
RS04405	RS04405	hypothetical protein	11.18	20.64	26.88	25.67
RS04410	RS04410	SLC13 family permease	6.45	8.2	15.69	24.06
RS04415	RS04415	C69 family dipeptidase	401.2	396.75	456.66	394.13
RS04420	RS04420	4-oxalocrotonate tautomerase	6.81	10.78	14.96	16.46
RS04425	RS04425	Cof-type HAD-IIB family hydrolase	25.32	31.6	67.14	32.02
RS04430	RS04430	RsmF rRNA methyltransferase first C-terminal domain-containing protein	20.49	38.45	43.71	45.57
RS04435	RS04435	hypothetical protein	4.04	3.54	6.32	11.11
RS04440	RS04440	L%2CD-transpeptidase/peptidoglycan binding protein	7.46	11.91	14.26	24.71
RS04445	RS04445	LLM class flavin-dependent oxidoreductase	8.05	5.4	7.9	13.49
RS04450	RS04450	NAD(P)-binding domain-containing protein	15.89	10.54	18.58	29.54
RS04455	RS04455	alpha/beta hydrolase	590.22	215.4	229.66	289.38
RS04460	RS04460	lactate oxidase	73.44	30.02	29.72	23.48
RS04465	RS04465	TMEM175 family protein	8.62	6.67	6.64	6.02
RS04470	fni	type 2 isopentenyl-diphosphate Delta-isomerase	49.75	30.98	59.42	56.15
RS04475	RS04475	phosphomevalonate kinase	44.27	35.76	63.29	65.4
RS04480	mvaD	diphosphomevalonate decarboxylase	48.34	47.88	84.97	71.57
RS04485	mvk	mevalonate kinase	47.82	39.36	92.41	84.38
RS04490	RS04490	exonuclease domain-containing protein	37.18	74.38	65.66	62.03
RS04495	RS04495	DUF5590 domain-containing protein	179.91	230.37	289.73	379.99
RS04500	RS04500	DnaD domain protein	26.1	53.42	83.85	121.45
RS04505	RS04505	penicillin-binding protein	285.75	414.86	308.03	470.81
RS04510	recU	Holliday junction resolvase RecU	74.62	110.59	91.98	107.34
RS04515	RS04515	DUF1273 domain-containing protein	29.66	42.58	50.32	70.41
RS04520	gpsB	cell division regulator GpsB	103234.06	84708.88	96248.33	95311.61
RS04525	RS04525	class I SAM-dependent RNA methyltransferase	44.36	31.8	53.43	33.55
RS04530	RS04530	FAD-binding oxidoreductase	54.7	41.24	52.5	50.37
RS04535	RS04535	hypothetical protein	51.04	28.84	42.32	49.19
RS04540	RS04540	formate--tetrahydrofolate ligase	213.84	106.38	110.26	102.73
RS04545	lspA	signal peptidase II	180.24	95.5	91.13	103.57

RS04550	RS04550	RluA family pseudouridine synthase	322.09	172.39	196.71	155.98
RS04555	pyrR	bifunctional pyr operon transcriptional regulator/uracil phosphoribosyltransferase PyrR	99.8	103.33	102.78	113.02
RS04560	RS04560	carbamoyl phosphate synthase small subunit	219.08	210.15	177.68	231.01
RS04565	RS04565	ATP-grasp domain-containing protein	245.34	215.69	190.69	241.14
RS04570	RS04570	fibronectin-binding domain-containing protein	13.95	70.37	58.89	69.75
RS04575	RS04575	MarR family transcriptional regulator	61.01	47.25	34.5	38.54
RS04580	RS04580	DegV family protein	40.12	49.53	64.14	47.7
RS04585	RS04585	CrcB family protein	3.78	3.61	6.58	7.46
RS04590	RS04590	CrcB family protein	5.24	5.24	10.29	9.32
RS04595	RS04595	DUF1516 family protein	5.59	3.23	15.86	5.5
RS04600	RS04600	multidrug efflux SMR transporter	11.04	11.23	15.07	16.13
RS04605	yjeM	glutamate/gamma-aminobutyrate family transporter YjeM	15.87	30.21	30.05	29.83
RS04610	msrA	peptide-methionine (S)-S-oxide reductase MsrA	1401.94	1253.66	699.65	756.47
RS04615	msrB	peptide-methionine (R)-S-oxide reductase MsrB	876.61	700.39	370.13	419.73
RS04620	RS04620	glycerate kinase	22.9	6.55	17.67	25.4
RS04625	RS04625	manganese-dependent inorganic pyrophosphatase	131.35	145.45	196.13	182.27
RS04630	RS04630	LysR family transcriptional regulator	108.68	116.67	180.27	157.03
RS04635	parC	DNA topoisomerase IV subunit A	52.03	29.28	47.72	34.74
RS04640	parE	DNA topoisomerase IV subunit B	44.79	45.43	77.26	61.72
RS04645	plsY	glycerol-3-phosphate 1-O-acyltransferase PlsY	7.24	3.69	14.27	8.66
RS04650	RS04650	aldose 1-epimerase family protein	147.99	160.29	223.56	396
RS04655	hslU	ATP-dependent protease ATPase subunit HslU	89.55	107.66	155.18	237.67
RS04660	hslV	ATP-dependent protease subunit HslV	93.41	110.35	191.86	229.81
RS04665	xerC	tyrosine recombinase XerC	37.42	59.89	92.23	100.25
RS04670	topA	type I DNA topoisomerase	26.95	181.68	267.97	362.55
RS04675	pgmB	beta-phosphoglucomutase	9.56	4.7	8.65	21.41
RS04680	RS04680	glycoside hydrolase family 65 protein	5.34	3.42	4.91	9.31
RS04685	RS04685	SLC45 family MFS transporter	3.18	2.86	2.56	3.82
RS04690	RS04690	LacI family DNA-binding transcriptional regulator	23.25	63.01	59.69	78.05
RS04695	dprA	DNA-processing protein DprA	7.01	6.04	23.59	53.11
RS04700	RS04700	ribonuclease HII	314.47	202.7	291.98	306.84
RS04705	ylqF	ribosome biogenesis GTPase YlqF	149.17	76.35	111.09	95.26
RS04725	RS04725	ATP-dependent Clp protease ATP-binding subunit	2421.86	927.53	916.16	1480.63
RS04730	RS04730	hypothetical protein	7.48	5.18	9.34	13.73
RS04735	RS04735	hypothetical protein	10.56	10.12	20.32	31.84
RS04740	RS04740	DUF916 and DUF3324 domain-containing protein	3.69	2.67	5.87	7.43
RS04745	RS04745	WxL domain-containing protein	4.26	3.38	5.75	8.6
RS04750	RS04750	hypothetical protein	2.67	1.16	2.59	4.09
RS04905	RS04905	hypothetical protein	0.46	0.22	0.3	0.07
RS04910	RS04910	hypothetical protein	7.18	2.1	2.6	6.67
RS04920	RS04920	ATP-binding protein	0.73	0.15	0.45	0.64
RS04930	RS04930	putative HNHc nuclease	3.2	1.72	3	6.32
RS04935	RS04935	single-stranded DNA-binding protein	0.1	0.14	0.25	0.15
RS04960	RS04960	hypothetical protein	3.29	0.42	2.16	1.84
RS04965	RS04965	hypothetical protein	39.11	65.67	64.91	44.43
RS04970	RS04970	DUF2829 domain-containing protein	9.51	3.48	4.86	7.31
RS04975	RS04975	phage antirepressor	2.33	1.08	1.36	3.15
RS05005	RS05005	site-specific integrase	3.32	2.92	6.22	5.33

RS05010	RS05010	helix-turn-helix domain-containing protein	15.84	14.25	24.08	23.94
RS05015	RS05015	TIGR02328 family protein	46.67	233.11	98.92	102.18
RS05035	RS05035	CocE/NonD family hydrolase	7.96	4.76	8.43	16.52
RS05040	RS05040	LysR family transcriptional regulator	5.88	24.72	60.34	43.77
RS05045	RS05045	MarR family transcriptional regulator	14.53	9.68	12.76	35.17
RS05050	RS05050	NAD(P)H-dependent oxidoreductase	2.61	1.93	4.72	10.3
RS05055	RS05055	hypothetical protein	7.48	21.83	28.04	102.71
RS05060	RS05060	hypothetical protein	4.96	11.93	18.51	47.89
RS05065	RS05065	Rgg/GadR/MutR family transcriptional regulator	4.79	52.32	65.53	155.98
RS05070	RS05070	nucleoside-diphosphate kinase	35.88	92.38	59.89	62.11
RS05075	RS05075	GNAT family N-acetyltransferase	4.09	10.79	25.97	24.48
RS05080	RS05080	APC family permease	9.95	49.12	99.47	65.69
RS05110	RS05110	arsenate reductase family protein	51.11	47.8	72.51	84.23
RS05115	RS05115	sodium:proton antiporter	10.92	24.29	56.88	51.9
RS05120	RS05120	S41 family peptidase	75.8	35.71	62.68	125.37
RS05125	RS05125	YozE family protein	26.55	14.34	25.23	52.94
RS05130	RS05130	YpmS family protein	20.82	15.73	29.03	47.68
RS05135	RS05135	SGNH/GDSL hydrolase family protein	21.77	28.69	46.06	68.84
RS05140	RS05140	DegV family protein	146.18	116.55	101.78	106.26
RS05145	RS05145	amino acid ABC transporter ATP-binding protein	97.09	343.24	92.56	197.25
RS05150	RS05150	amino acid ABC transporter substrate-binding protein/permease	57.27	127.68	54.07	94.7
RS05155	RS05155	dihydrofolate reductase	96.2	97.55	68.77	99.92
RS05160	RS05160	thymidylate synthase	115.61	133.92	96.22	164.4
RS05165	RS05165	ABC-F family ATP-binding cassette domain-containing protein	88.99	111.29	117.89	127.14
RS05170	RS05170	CCA tRNA nucleotidyltransferase	28.81	27.7	38.7	28.01
RS05175	RS05175	YitT family protein	34.06	62.42	52.43	32.92
RS05180	RS05180	nucleoside 2-deoxyribosyltransferase	47.36	251.45	25.99	17.76
RS05185	RS05185	hypothetical protein	9.06	4.79	6.82	13.45
RS05190	RS05190	tetratricopeptide repeat protein	69.81	105.92	117.11	159.73
RS05195	RS05195	HU family DNA-binding protein	2507.41	2607.14	3633.19	2213.62
RS05205	rpsA	30S ribosomal protein S1	421.5	392.11	485.14	465.61
RS05210	cmk	(d)CMP kinase	81.22	110.39	186.87	172.99
RS05215	RS05215	LysM peptidoglycan-binding domain-containing protein	117.16	222.95	293.55	222.9
RS05220	RS05220	RecQ family ATP-dependent DNA helicase	50.26	10.28	25.95	29.82
RS05225	RS05225	helix-turn-helix domain-containing protein	32.74	13.48	26.64	16.24
RS05230	RS05230	rRNA pseudouridine synthase	84.76	30.53	63.31	45.27
RS05235	scpB	SMC-Scp complex subunit ScpB	61.72	30.04	59.07	36.97
RS05240	RS05240	segregation/condensation protein A	84.86	44.95	87.65	57.06
RS05245	RS05245	hypothetical protein	105.45	62.95	115.54	99.21
RS05250	xerD	site-specific tyrosine recombinase XerD	73.15	44.45	81.17	63.44
RS05255	RS05255	S1-like domain-containing RNA-binding protein	57.12	27.12	59.8	42.87
RS05260	RS05260	DUF441 domain-containing protein	33.15	33.87	61.27	33.57
RS05265	RS05265	DUF805 domain-containing protein	268.12	593.92	377.88	387.66
**RS05270**	**pyk**	**pyruvate kinase**	**625.75**	**322.33**	**420.24**	**275.09**
**RS05275**	**pfkA**	**6-phosphofructokinase**	**288.28**	**136.76**	**234.32**	**136.94**
RS05280	dnaE	DNA polymerase III subunit alpha	112.14	111.41	53.63	81.33
RS05290	clpB	ATP-dependent chaperone ClpB	157.59	276.96	150.75	595.43
RS05295	RS05295	Nif3-like dinuclear metal center hexameric protein	52.31	81.73	53.4	114.28
RS05300	RS05300	class I SAM-dependent methyltransferase	122.54	243.52	157.35	330.17

RS05305	rpoD	RNA polymerase sigma factor RpoD	115.47	220.11	158.3	214.98
RS05310	dnaG	DNA primase	82.59	210.05	166.65	156.98
RS05315	glyS	glycine--tRNA ligase subunit beta	93.51	23.7	56.62	63.78
RS05320	glyQ	glycine--tRNA ligase subunit alpha	54.32	10.29	33.38	28.2
RS05325	recO	DNA repair protein RecO	230.75	162.88	138.36	98.99
RS05330	era	GTPase Era	360.59	204.89	219.77	152.65
RS05335	RS05335	diacylglycerol kinase family protein	146.04	65.06	82.19	55.42
RS05340	ybeY	rRNA maturation RNase YbeY	266.27	106.04	131.01	81.83
RS05345	RS05345	PhoH family protein	441.97	167.77	197.65	134.55
RS05350	RS05350	GatB/YqeY domain-containing protein	668.02	236.64	274.72	192.52
RS05355	rpsU	30S ribosomal protein S21	4327.03	5255.51	3548.79	2201.7
RS05360	RS05360	kinase/pyrophosphorylase	146.78	53.69	76.77	74.24
RS05365	RS05365	deoxyribonuclease IV	81.07	88.96	108.24	160.84
RS05370	RS05370	CDP-glycerol glycerophosphotransferase family protein	33.2	42.55	52.74	60.16
RS05375	RS05375	CDP-glycerol glycerophosphotransferase family protein	20.11	28.17	37.43	28.6
RS05380	RS05380	YitT family protein	32.75	49.79	66.78	46.87
RS05385	aspS	aspartate--tRNA ligase	131.28	24.57	54.55	55.48
RS05390	hisS	histidine--tRNA ligase	63.89	8.32	25.9	23.08
RS05395	RS05395	N-acetylmuramoyl-L-alanine amidase	47.94	14.67	15.75	17.4
RS05400	RS05400	HAD-IA family hydrolase	32.92	26.1	30.17	74
RS05405	dtd	D-aminoacyl-tRNA deacylase	260.56	324.69	197.45	373.14
RS05410	RS05410	bifunctional (p)ppGpp synthetase/guanosine-3'%2C5'-bis(diphosphate) 3'-pyrophosphohydrolase	161.54	210.5	156.84	249.66
RS05420	RS05420	16S rRNA (uracil(1498)-N(3))-methyltransferase	137.59	100.17	146.57	113.13
RS05425	prmA	50S ribosomal protein L11 methyltransferase	42.79	32.37	64.01	37.77
RS05430	RS05430	NCS2 family nucleobase:cation symporter	326.6	149.22	134.34	52.27
RS05435	RS05435	hypothetical protein	132.86	198.68	191.75	160.32
RS05440	RS05440	DMT family transporter	4.56	21.3	30.66	65.9
RS05445	nfsA	oxygen-insensitive NADPH nitroreductase	16.5	31.99	112.6	79.01
RS05455	RS05455	adenine phosphoribosyltransferase	166.64	177.41	202.21	322.41
**RS05460**	**recJ**	**single-stranded-DNA-specific exonuclease RecJ**	**75.86**	**148.35**	**139.1**	**250.16**
RS05465	RS05465	SDR family NAD(P)-dependent oxidoreductase	44.15	126.36	118.93	165.52
RS05470	rnz	ribonuclease Z	36.5	77.45	95.37	119.61
RS05475	RS05475	hypothetical protein	5.35	18.38	18.63	14.26
RS05480	obgE	GTPase ObgE	195.67	265.62	194.26	244.37
RS05485	uvrC	excinuclease ABC subunit UvrC	39.62	158.91	83.45	130.82
**RS05490**	**RS05490**	**amino acid ABC transporter ATP-binding protein**	**45.75**	**175.68**	**148.21**	**647.44**
**RS05495**	**RS05495**	**ABC transporter substrate-binding protein/permease**	**13.19**	**73.39**	**74.89**	**236.35**
RS05500	RS05500	nucleoside triphosphate pyrophosphohydrolase family protein	275.86	115.21	188.63	244.11
RS05505	yihA	ribosome biogenesis GTP-binding protein YihA/YsxC	74.65	24.03	48.08	50.98
RS05510	clpX	ATP-dependent Clp protease ATP-binding subunit ClpX	318.06	283.35	279.15	230.48
RS05515	Tig	trigger factor	1152.41	670.09	896.75	712.28
RS05520	tuf	elongation factor Tu	6888.83	4677.06	6419	4987.43
RS05525	RS05525	hypothetical protein	24.49	17.96	31.73	39.56
RS05530	RS05530	ribonuclease J	234.45	149.88	171.57	142.04
RS05535	rpsO	30S ribosomal protein S15	596.11	1115.52	1575.03	1147.75
RS05540	rpsT	30S ribosomal protein S20	3098.53	5597.06	8434.78	7840.48
RS05545	holA	DNA polymerase III subunit delta	22.84	20.26	43.7	41.6
RS05550	RS05550	DNA internalization-related competence protein ComEC/Rec2	11.63	15.4	16.62	35.04

RS05555	RS05555	ComE operon protein 2	28.36	21.42	30.79	31.57
RS05560	RS05560	helix-hairpin-helix domain-containing protein	14.33	15.98	19.22	23.45
RS05565	coaD	pantetheine-phosphate adenylyltransferase	68.24	40.28	71.92	91.36
RS05570	rsmD	16S rRNA (guanine(966)-N(2))-methyltransferase RsmD	71.92	40.53	76.13	111.83
RS05575	RS05575	DUF2129 domain-containing protein	42.32	29.32	55.45	65.54
RS05580	RS05580	FtsW/RodA/SpoVE family cell cycle protein	45.57	49.26	69.78	93.25
RS05585	typA	translational GTPase TypA	260.94	265.08	314.89	280.06
RS05590	RS05590	inositol monophosphatase family protein	170.91	70.47	112.36	130.85
RS05595	RS05595	UPF0223 family protein	26.91	9.85	14.6	9.77
RS05600	def	peptide deformylase	221.76	182.13	281.64	135.52
RS05605	RS05605	hypothetical protein	61.5	67.93	84.19	59
RS05610	RS05610	DNA-directed RNA polymerase subunit epsilon	34.7	23.42	30.7	16.55
RS05615	RS05615	ribonuclease J	854.16	324.25	363.41	296.32
RS05620	RS05620	peptidoglycan endopeptidase	255.93	202.5	598.16	92.38
RS05625	RS05625	fructose-specific PTS transporter subunit EIIC	46.7	7.6	46.02	115.1
RS05630	pfkB	1-phosphofructokinase	46.84	7.27	112.47	244.86
RS05635	RS05635	DeoR/GlpR family DNA-binding transcription regulator	41.12	12.99	277.61	548.99
RS05640	RS05640	AI-2E family transporter	29.42	79.64	94.92	103.93
RS05645	RS05645	ribose-phosphate diphosphokinase	296.4	112.11	151.62	144.42
RS05650	RS05650	ATP-dependent RecD-like DNA helicase	30.68	12.26	32.58	28.4
RS05655	RS05655	tetratricopeptide repeat protein	22.13	11.49	23.71	14.43
RS05660	RS05660	histidine phosphatase family protein	42.86	22.46	48.17	24.89
RS05665	mnmA	tRNA 2-thiouridine(34) synthase MnmA	455.34	415.58	403.55	646.6
RS05670	RS05670	DUF1831 domain-containing protein	59.31	58.84	62.18	87.47
RS05675	RS05675	cysteine desulfurase	244.34	244.85	277.13	394.45
RS05680	RS05680	5'-methylthioadenosine/adenosylhomocysteine nucleosidase	285.23	276.29	325.72	445.46
RS05685	RS05685	hypothetical protein	499.49	523.72	588.91	848.68
RS05690	RS05690	NUDIX hydrolase	150.56	98.31	136.47	139.98
RS05695	RS05695	cold shock domain-containing protein	260.39	227.29	271.13	321.17
RS05700	ileS	isoleucine--tRNA ligase	96.31	40.36	65.8	88.73
RS05705	RS05705	DivIVA domain-containing protein	246.62	285.19	401.6	436.67
RS05710	RS05710	YlmH/Sll1252 family protein	61.81	64.47	80.08	82.69
RS05715	RS05715	YggT family protein	78.55	86.99	103.28	99.75
RS05720	ftsZ	cell division protein FtsZ	462.04	469.57	566.13	550.48
RS05725	ftsA	cell division protein FtsA	351.62	379.21	472.72	366.83
RS05730	RS05730	FtsQ-type POTRA domain-containing protein	53.71	11.81	36.19	22.52
RS05735	murG	undecaprenyldiphospho-muramoylpentapeptide beta-N-acetylglucosaminyltransferase	67.38	10.86	41.19	24.48
RS05740	murD	UDP-N-acetylmuramoyl-L-alanine--D-glutamate ligase	92.84	8.07	34.08	16.43
RS05745	mraY	phospho-N-acetylmuramoyl-pentapeptide-transferase	52.97	18.68	41.45	44.57
RS05750	RS05750	penicillin-binding protein	40.16	10.16	23.22	16.19
RS05755	ftsL	cell division protein FtsL	35.23	22.53	17.37	26.66
RS05760	rsmH	16S rRNA (cytosine(1402)-N(4))-methyltransferase RsmH	94.6	90.92	69.13	115.9
RS05765	mraZ	division/cell wall cluster transcriptional repressor MraZ	32.97	43.6	41.11	58.96
RS05770	RS05770	DNA translocase FtsK	39.87	14.08	42.06	25.61
RS05775	RS05775	tRNA (cytidine(34)-2'-O)-methyltransferase	44.77	18.57	58.37	32.12
RS05780	RS05780	AI-2E family transporter	87.9	94.15	61.69	48.47
RS05785	RS05785	PTS glucitol/sorbitol transporter subunit IIA	140.33	251.52	115.37	90.83
RS05790	RS05790	lactonase family protein	84.08	55.62	84.76	42.32

RS05795	RS05795	copper homeostasis protein CutC	221.71	93.53	130.88	70.46
RS05800	mgtE	magnesium transporter	65.9	13.29	25.58	15.67
RS05805	RS05805	RluA family pseudouridine synthase	105.5	43.42	62.72	24.15
RS05810	RS05810	NAD kinase	53.67	34.47	53.12	32.49
RS05815	RS05815	GTP pyrophosphokinase family protein	49.5	31.11	56.8	23.3
RS05820	RS05820	DsbA family protein	1131.19	272.86	518.5	269.27
RS05825	RS05825	competence protein CoiA family protein	3.71	1.1	2.32	3.93
RS05830	RS05830	adaptor protein MecA	256.47	323.78	188.84	182.37
RS05835	spxA	transcriptional regulator SpxA	3661.42	2714.84	1643	1429.36
RS05840	RS05840	MBL fold metallo-hydrolase	119.5	26.78	66.75	26.43
RS05845	RS05845	hypothetical protein	91.24	76.43	82.19	66.09
RS05920	RS05920	S1 RNA-binding domain-containing protein	59.94	39.36	70.24	52.04
RS05925	RS05925	VTT domain-containing protein	7.72	10.49	13.12	11.41
RS05930	RS05930	TIGR01906 family membrane protein	13.98	9.59	27.39	27.02
RS05935	RS05935	TIGR01457 family HAD-type hydrolase	45.58	26.74	101.9	59.76
RS05940	RS05940	YutD family protein	57.73	45.63	162.55	85.09
RS05945	RS05945	metallophosphoesterase	23.32	57.89	67.72	53.57
RS05950	RS05950	amino acid permease	38.13	136.48	155.5	120.14
RS05955	RS05955	class I SAM-dependent methyltransferase	33.75	73.64	170.77	253.35
RS05960	RS05960	hypothetical protein	3.33	0.74	2.88	3.67
RS05965	RS05965	ComGF family competence protein	3.34	1.23	3.27	4.07
RS05975	RS05975	type II secretion system protein	8.58	3.43	7.45	11.46
RS05980	comGC	competence type IV pilus major pilin ComGC	3.24	1.87	3.02	3.28
RS05985	comGB	competence type IV pilus assembly protein ComGB	5.53	2.04	4.48	7.36
RS05990	comGA	competence type IV pilus ATPase ComGA	10.34	4.52	8.13	17.72
RS05995	rbsK	ribokinase	31.64	24.86	39.14	19.41
RS06000	RS06000	YebC/PmpR family DNA-binding transcriptional regulator	277.93	300.09	514.26	408.56
RS06005	RS06005	hypothetical protein	54.97	9.21	22.3	23.32
RS06010	RS06010	hypothetical protein	62.76	12.15	29.05	34.04
RS06015	RS06015	Y-family DNA polymerase	63.32	12.86	24.54	22.86
RS06020	efp	elongation factor P	156.31	102.9	169.09	116.15
RS06025	RS06025	cysteine hydrolase	405.38	683.23	331.56	271.55
**RS06030**	**purB**	**adenylosuccinate lyase**	**81.95**	**115.22**	**33.3**	**27.73**
RS06035	RS06035	adenylosuccinate synthase	72.12	131.71	50.98	60.52
RS06040	RS06040	hypothetical protein	68.1	34.56	71.57	56.88
RS06045	RS06045	aquaporin family protein	4.28	5.1	16.85	8.66
RS06050	ccpA	catabolite control protein A	110.26	55.65	72.72	54.79
RS06055	RS06055	Xaa-Pro peptidase family protein	147.76	87.39	122.69	87.46
**RS06060**	**spxB**	**pyruvate oxidase**	**1035.48**	**432.01**	**207.36**	**164.88**
RS06065	RS06065	hypothetical protein	434.3	219.29	196.52	175.8
RS06070	RS06070	DUF948 domain-containing protein	2358.5	1926.92	1560.68	1736.73
RS06075	RS06075	mechanosensitive ion channel	25.28	62.4	81.13	50.9
RS06080	RS06080	XTP/dITP diphosphatase	192.55	256.38	189.28	225.72
RS06085	murI	glutamate racemase	99.36	145.37	111.94	120.48
RS06090	trxA	thioredoxin	1876.68	1421.58	851.33	886.87
**RS06095**	**RS06095**	**endonuclease MutS2**	**110.82**	**208.79**	**164.49**	**325.96**
RS06100	RS06100	DUF1292 domain-containing protein	218.15	182.08	160.76	130.25
RS06105	ruvX	Holliday junction resolvase RuvX	1638.32	1291.79	1089.21	1321.96
RS06110	RS06110	IreB family regulatory phosphoprotein	273.23	216.2	183.87	151.17

RS06115	alaS	alanine--tRNA ligase	68.97	35.29	49.53	78.64
RS06120	RS06120	DEAD/DEAH box helicase	260.24	235.85	280.66	298.93
RS06125	RS06125	bifunctional oligoribonuclease/PAP phosphatase NrnA	162.61	123.52	168.97	161.19
RS06130	dinB	DNA polymerase IV	110.12	115.31	121.99	270.56
RS06135	zwf	glucose-6-phosphate dehydrogenase	68.66	92.95	99.25	135.88
RS06140	yajC	preprotein translocase subunit YajC	316.71	460.59	843.66	321.18
RS06145	Tgt	tRNA guanosine(34) transglycosylase Tgt	188.57	169.94	207.57	263.93
RS06150	queA	tRNA preQ1(34) S-adenosylmethionine ribosyltransferase-isomerase QueA	175.45	123.37	161.57	202.77
RS06155	ruvB	Holliday junction branch migration DNA helicase RuvB	221.91	146.08	192.31	258.79
RS06160	ruvA	Holliday junction branch migration protein RuvA	29.28	32.64	39.39	39.21
RS06165	mutL	DNA mismatch repair endonuclease MutL	78.78	81.95	106.08	157.93
**RS06170**	**mutS**	**DNA mismatch repair protein MutS**	**40.35**	**51.41**	**82.41**	**92.7**
RS06175	rny	ribonuclease Y	221.31	193.66	261.52	205.04
RS06180	recA	recombinase RecA	928.51	1199.19	838.96	718.32
RS06185	RS06185	competence/damage-inducible protein A	260.11	70.58	121.8	105.87
RS06190	pgsA	CDP-diacylglycerol--glycerol-3-phosphate 3-phosphatidyltransferase	74.23	48.75	83.2	58.52
RS06195	RS06195	helix-turn-helix domain-containing protein	95.32	54.91	76.05	42.02
RS06200	RS06200	SDR family oxidoreductase	54	33.03	81.88	40.62
RS06205	RS06205	insulinase family protein	18.51	18.95	34.66	14.52
RS06210	RS06210	insulinase family protein	45.91	97.1	130.43	67.87
RS06215	Cls	cardiolipin synthase	19.84	56.45	36.15	26.74
RS06220	RS06220	amino acid ABC transporter substrate-binding protein	18.43	9.98	21.91	44.73
RS06225	RS06225	ATP-binding cassette domain-containing protein	16.29	6.67	16.68	33.13
RS06230	RS06230	amino acid ABC transporter permease	11.1	7.61	14.08	18.29
RS06235	mreD	rod shape-determining protein MreD	101.73	83.09	85	143.89
RS06240	mreC	rod shape-determining protein MreC	117.71	197.04	209.72	270.55
RS06245	radC	DNA repair protein RadC	15.9	17.05	24.22	48.31
RS06250	RS06250	bifunctional folylpolyglutamate synthase/dihydrofolate synthase	27.18	21.94	39.02	90.3
RS06255	RS06255	valine--tRNA ligase	154.6	72.61	138.37	163.63
RS06260	tpx	thiol peroxidase	823.04	603.67	663.4	441.46
RS06265	thiI	tRNA 4-thiouridine(8) synthase ThiI	128.99	62.57	100.83	72.66
RS06270	RS06270	cysteine desulfurase	107.54	50.57	108.34	63.2
RS06275	ezrA	septation ring formation regulator EzrA	254.09	215.16	133.56	141.94
RS06280	RS06280	GAF domain-containing protein	43.78	95.26	104.59	110.19
RS06285	rpsD	30S ribosomal protein S4	1669.1	2640.68	2911.18	3558.89
RS06290	RS06290	replication-associated recombination protein A	202.79	61.85	137.88	139.64
RS06300	RS06300	universal stress protein	1205.34	597.1	327.08	282.55
RS06305	RS06305	DNA-3-methyladenine glycosylase I	3.86	4.22	9.56	7.04
RS06310	RS06310	D-alanine--D-alanine ligase	130.38	271.25	266.27	188.59
RS06315	RS06315	DUF2785 domain-containing protein	52.39	188.94	89.67	79.9
RS06320	RS06320	amino acid permease	19.15	163.09	151.18	120.27
RS06325	RS06325	DUF975 family protein	127.57	190.81	190.22	251.51
RS06330	RS06330	hypothetical protein	100.08	70.96	62.98	156.26
RS06335	RS06335	ABC transporter ATP-binding protein	72.9	68.44	52.06	109.46
RS06340	RS06340	GntR family transcriptional regulator	53.83	62.3	44.67	106.6
RS06345	RS06345	MetQ/NlpA family ABC transporter substrate-binding protein	26.09	59.05	61.07	170.36
RS06350	RS06350	ABC transporter permease	12.44	20.9	22.79	53.5

RS06355	RS06355	methionine ABC transporter ATP-binding protein	11.58	31.41	43.88	56.98
RS06360	RS06360	rod shape-determining protein RodA	319.6	238.08	259.29	213.67
RS06365	RS06365	DUF2969 domain-containing protein	35.19	65.91	33.49	23.63
RS06370	yidD	membrane protein insertion efficiency factor YidD	71.71	157.77	170.15	86.33
RS06375	RS06375	DUF1146 family protein	27.66	142.08	133.15	77.5
**RS06380**	**RS06380**	**F0F1 ATP synthase subunit epsilon**	**1181.58**	**463.54**	**856.08**	**512.9**
**RS06385**	**atpD**	**F0F1 ATP synthase subunit beta**	**748.87**	**236.78**	**504.91**	**314.75**
**RS06390**	**RS06390**	**F0F1 ATP synthase subunit gamma**	**885**	**267.81**	**598.48**	**381.63**
**RS06395**	**atpA**	**F0F1 ATP synthase subunit alpha**	**678.34**	**174.74**	**422.55**	**230.37**
**RS06400**	**atpH**	**ATP synthase F1 subunit delta**	**761.8**	**119.06**	**423.61**	**207.32**
**RS06405**	**atpF**	**F0F1 ATP synthase subunit B**	**153.69**	**33.19**	**94.3**	**48.89**
**RS06410**	**atpE**	**F0F1 ATP synthase subunit C**	**520.75**	**151.17**	**381.94**	**231.18**
**RS06415**	**atpB**	**F0F1 ATP synthase subunit A**	**201.28**	**81.23**	**182.03**	**110.96**
RS06420	RS06420	ABC transporter permease	16.92	14.32	15.94	19.46
RS06425	RS06425	ABC transporter ATP-binding protein	13.15	16	14.45	14.6
RS06430	upp	uracil phosphoribosyltransferase	964.25	859.02	910.87	1098.81
RS06435	RS06435	serine hydroxymethyltransferase	267.72	343.15	288.89	341.81
RS06440	RS06440	L-threonylcarbamoyladenylate synthase	152.42	137.05	185.33	96.23
RS06445	prmC	peptide chain release factor N(5)-glutamine methyltransferase	140.71	50.93	110.58	57.97
RS06450	prfA	peptide chain release factor 1	144.93	37.88	118.78	42.15
RS06455	RS06455	thymidine kinase	54.92	29.56	77.64	28.85
**RS06460**	**RS06460**	**Mur ligase family protein**	**292.84**	**636.31**	**401.24**	**688.93**
RS06465	RS06465	glutamine amidotransferase	60.63	94.07	57.04	95.92
RS06470	manA	mannose-6-phosphate isomerase%2C class I	222.25	46.96	82.83	43.74
RS06475	RS06475	serine hydrolase	43.89	11.07	29.69	27.67
RS06480	RS06480	ABC transporter ATP-binding protein/permease	38.51	162.32	180.1	445.04
RS06485	RS06485	ABC transporter ATP-binding protein/permease	13.76	89.16	144.78	184.45
RS06490	RS06490	TetR/AcrR family transcriptional regulator	11.3	74.88	169.41	88.85
RS06495	RS06495	hypothetical protein	32.91	154.8	126.66	74.03
RS06500	greA	transcription elongation factor GreA	24.37	110.48	80.11	41.25
RS06515	RS06515	rhodanese-like domain-containing protein	46.42	38.83	114.68	114.41
RS06520	RS06520	SPFH/Band 7/PHB domain protein	13.76	2.96	8.83	10.01
RS06525	RS06525	toxin-antitoxin system HicB family antitoxin	41.6	5.8	24.18	24.51
RS06530	RS06530	PAS domain-containing protein	73.05	14.56	38.54	26.62
RS06535	RS06535	NAD(P)H-dependent oxidoreductase	33.43	13.04	18.21	12.51
RS06540	RS06540	FAD:protein FMN transferase	25.94	32.19	24.98	29.49
**RS06545**	**RS06545**	**glucose-6-phosphate isomerase**	**2267.97**	**916.29**	**1639.37**	**1046.02**
RS06550	RS06550	GRP family sugar transporter	14.12	24.16	164.28	65.29
**RS06555**	**fba**	**class II fructose-1%2C6-bisphosphate aldolase**	**2413.84**	**701.47**	**2110.38**	**450.71**
RS06560	RS06560	HAMP domain-containing histidine kinase	27.95	48.01	96.86	217.39
RS06565	RS06565	response regulator transcription factor	35.34	34.59	103.77	155.94
RS06570	RS06570	LysM peptidoglycan-binding domain-containing protein	49.18	371.96	2623.55	894.15
RS06575	RS06575	LysM peptidoglycan-binding domain-containing protein	45.47	921.25	3776.42	1331.03
RS06580	RS06580	GNAT family N-acetyltransferase	13.46	9.6	19.66	19.01
RS06585	RS06585	hypothetical protein	651.91	1498.59	515.7	467.98
RS06590	RS06590	YxeA family protein	190.38	2119.65	505.2	850.5
RS06595	RS06595	gluconate:H+ symporter	19.18	6.36	15.34	29.28
RS06600	gntK	gluconokinase	39.63	15.46	35.54	53.51
RS06605	gnd	decarboxylating 6-phosphogluconate dehydrogenase	28.74	21.25	35.88	32.61

RS06610	RS06610	MurR/RpiR family transcriptional regulator	15.54	8.66	11.04	10.24
RS06620	rlmD	23S rRNA (uracil(1939)-C(5))-methyltransferase RlmD	84.61	75.7	176.64	153.14
RS06625	RS06625	diacylglycerol kinase	400.51	236.45	446.3	417.63
RS06630	gatB	Asp-tRNA(Asn)/Glu-tRNA(Gln) amidotransferase subunit GatB	404.25	203.09	390.79	369.48
RS06635	gatA	Asp-tRNA(Asn)/Glu-tRNA(Gln) amidotransferase subunit GatA	233.31	79.71	200.85	115.87
RS06640	gatC	Asp-tRNA(Asn)/Glu-tRNA(Gln) amidotransferase subunit GatC	147.87	76.24	154.46	94.75
RS06645	RS06645	CamS family sex pheromone protein	142.87	99.14	165.49	115.88
RS06650	pcrA	DNA helicase PcrA	62.87	98.63	149.63	111.79
RS06655	RS06655	ATP-grasp domain-containing protein	338.25	141.59	415.37	204.07
RS06660	RS06660	xanthine phosphoribosyltransferase	89.06	42.44	102.47	36.81
RS06665	RS06665	glycoside hydrolase family 73 protein	105.73	197.32	299.32	115.95
RS06670	RS06670	ECF transporter S component	18.72	15.81	38.62	18.06
RS06675	RS06675	phosphoglycerate dehydrogenase	705	255.96	259.25	224.65
RS06680	RS06680	ABC transporter permease	10.24	4.31	8.02	13.06
RS06685	RS06685	DEAD/DEAH box helicase	38.34	47.43	60.82	69.58
RS06695	RS06695	DNA starvation/stationary phase protection protein	1856.46	1155.66	754.32	751.43
RS06700	RS06700	helix-turn-helix transcriptional regulator	5.24	3.13	6.74	6.46
RS06705	RS06705	helix-turn-helix domain-containing protein	18.78	3.46	8.15	8.7
RS06710	rpsI	30S ribosomal protein S9	469.4	360.23	585.41	327.71
RS06715	rplM	50S ribosomal protein L13	2040.37	1603.74	2704.01	1573.59
RS06720	truA	tRNA pseudouridine(38-40) synthase TruA	81.93	75.24	116.77	139.21
RS06725	RS06725	energy-coupling factor transporter transmembrane protein EcfT	53.81	45.31	75.08	68.83
RS06730	RS06730	energy-coupling factor ABC transporter ATP-binding protein	73.76	48.44	88.64	74.76
RS06735	RS06735	energy-coupling factor transporter ATPase	64	23.45	55.24	32.03
RS06740	rplQ	50S ribosomal protein L17	576.3	1222.85	2019.64	2680.81
RS06745	RS06745	DNA-directed RNA polymerase subunit alpha	831.03	1031.67	1742.47	2666.26
RS06750	rpsK	30S ribosomal protein S11	324.86	247.73	442.02	561.42
RS06755	rpsM	30S ribosomal protein S13	1130.32	1119.74	2092.89	2939.96
RS06760	rpmJ	50S ribosomal protein L36	2338.53	2383.85	4085.19	5642.36
RS06765	infA	translation initiation factor IF-1	60.06	86.7	135.45	123.21
RS06770	RS06770	adenylate kinase	168.91	240.46	260.65	318.52
RS06775	secY	preprotein translocase subunit SecY	328.59	515.14	486.25	694.56
RS06780	rplO	50S ribosomal protein L15	417.74	848.04	771.05	891.4
RS06785	rpmD	50S ribosomal protein L30	356.36	609.1	502.61	747.34
RS06790	rpsE	30S ribosomal protein S5	404.25	742.85	630.86	731.97
RS06795	rplR	50S ribosomal protein L18	1220.8	2687.28	2045.55	3292.26
RS06800	rplF	50S ribosomal protein L6	886.84	1979.59	1630.66	2145.18
RS06805	rpsH	30S ribosomal protein S8	400.19	811.03	716.8	865.67
RS06810	RS06810	type Z 30S ribosomal protein S14	1158.34	2553.39	2187.34	2803.62
RS06815	rplE	50S ribosomal protein L5	780.62	1570.64	1405.73	2119.53
RS06820	rplX	50S ribosomal protein L24	535	1088.55	1051.63	1395.78
RS06825	rplN	50S ribosomal protein L14	145.31	253.87	256.35	285.74
RS06830	rpsQ	30S ribosomal protein S17	1284.84	2376.25	2287.65	2922.29
RS06835	rpmC	50S ribosomal protein L29	3373.4	6237.59	5531.45	8187.51
RS06840	rplP	50S ribosomal protein L16	1060.18	2058.54	2045.86	2343.21
RS06845	rpsC	30S ribosomal protein S3	798.49	1527.12	1536.87	1630.6
RS06850	rplV	50S ribosomal protein L22	1183.57	2179.25	2215.51	2974.13
RS06855	rpsS	30S ribosomal protein S19	823.31	1585.86	1548.31	1661.92
RS06860	rplB	50S ribosomal protein L2	202.83	421.55	405.81	404.52

RS06865	rplW	50S ribosomal protein L23	2249.22	5485.9	5369.98	8762.13
RS06870	rplD	50S ribosomal protein L4	191.15	492.16	466.48	473.63
RS06875	rplC	50S ribosomal protein L3	1128.68	3405.02	3300.74	3982.27
RS06880	rpsJ	30S ribosomal protein S10	2968.6	11401.54	10657.81	18288.84
RS06885	fusA	elongation factor G	425.19	1167.53	711.72	1126.28
RS06890	rpsG	30S ribosomal protein S7	875.96	2246.46	1895.31	2323.07
RS06895	rpsL	30S ribosomal protein S12	370.93	1022.72	1066.53	1126.5
RS06905	RS06905	nucleoside hydrolase	41.61	98.19	10.73	13.97
RS06910	rpoC	DNA-directed RNA polymerase subunit beta'	222.4	322.09	307.53	739.32
RS06915	RS06915	DNA-directed RNA polymerase subunit beta	269	590.56	632.34	857.67
RS06920	RS06920	ATP-dependent Clp protease ATP-binding subunit	366.96	724.88	380.34	543.96
RS06925	RS06925	CtsR family transcriptional regulator	130.96	260.27	135.05	147.32
RS06960	serS	serine--tRNA ligase	151.78	52.7	88.13	123.88
RS06965	RS06965	deoxynucleoside kinase	158.22	34.04	93.72	72.98
RS06970	RS06970	amino acid permease	136.28	42.49	80.1	84.04
RS06975	RS06975	GNAT family N-acetyltransferase	56.48	18.97	33.43	38.52
RS06980	RS06980	alpha/beta hydrolase	404.79	150.67	143.31	150.7
RS06985	RS06985	LCP family protein	757.65	374.68	479.77	388.68
RS06990	RS06990	ROK family protein	75.44	24.47	46.42	38.54
RS06995	RS06995	threonine/serine exporter family protein	31.21	31.55	65.27	56.87
RS07000	RS07000	threonine/serine exporter family protein	18.22	20.87	35.13	29.92
RS07005	RS07005	cold-shock protein	1995.27	4148.01	5044.82	3130.52
RS07010	RS07010	phosphatase PAP2 family protein	241.33	167.61	209.74	248.71
RS07015	RS07015	HD domain-containing protein	93.25	71.34	85.17	100.01
RS07020	rny	ribonuclease Y	123.55	148.38	165.53	168.77
RS07025	RS07025	hypothetical protein	179.23	92.77	117.21	104.25
RS07030	RS07030	RluA family pseudouridine synthase	98.23	55.26	64.99	34.82
RS07035	RS07035	folate family ECF transporter S component	3445.51	1895.21	2358.74	1991.54
RS07040	RS07040	hypothetical protein	43.2	24	31.77	41.75
RS07045	RS07045	purine permease	14.03	88.58	56.12	43.48
RS07050	RS07050	C69 family dipeptidase	307.59	198.26	369.52	141.93
RS07055	RS07055	hypothetical protein	18.49	6	9.68	11.6
RS07060	purD	phosphoribosylamine--glycine ligase	20.4	18.23	20.85	32.27
RS07065	purH	bifunctional phosphoribosylaminoimidazolecarboxamide formyltransferase/IMP cyclohydrolase	14.42	11.68	11.57	16.5
RS07070	purN	phosphoribosylglycinamide formyltransferase	20.27	15.2	11.84	16.05
RS07075	purM	phosphoribosylformylglycinamidine cyclo-ligase	16.1	17.91	10.22	15.92
RS07080	purF	amidophosphoribosyltransferase	12.5	22.57	9.16	16.36
RS07085	purL	phosphoribosylformylglycinamidine synthase subunit PurL	13.09	27.41	10.75	17.73
RS07090	purQ	phosphoribosylformylglycinamidine synthase subunit PurQ	6.66	10.47	4.17	6.85
RS07095	purS	phosphoribosylformylglycinamidine synthase subunit PurS	10.7	23.34	7.21	12.91
RS07100	RS07100	phosphoribosylaminoimidazolesuccinocarboxamide synthase	15.1	21.92	9.39	14.63
RS07105	purK	5-(carboxyamino)imidazole ribonucleotide synthase	37.7	70.15	21.25	28.39
RS07110	purE	5-(carboxyamino)imidazole ribonucleotide mutase	38.45	51.43	17.48	13.13
**RS07120**	**asnA**	**aspartate--ammonia ligase**	**43.94**	**221.4**	**390.29**	**127.64**
RS07125	RS07125	redox-sensing transcriptional repressor Rex	202.32	194.72	164.14	351.59
RS07130	RS07130	serine hydrolase	123.14	136.26	122.58	238.22
RS07135	tsaD	tRNA (adenosine(37)-N6)-threonylcarbamoyltransferase complex transferase subunit TsaD	91.58	85.97	95.81	171.69

RS07140	rimI	ribosomal protein S18-alanine N-acetyltransferase	111.28	82.2	106.03	207.13
RS07145	tsaB	tRNA (adenosine(37)-N6)-threonylcarbamoyltransferase complex dimerization subunit type 1 TsaB	60.1	43.34	60.82	82.2
RS07150	galE	UDP-glucose 4-epimerase GalE	526.31	258.66	320.17	363.48
RS07155	RS07155	thioesterase	217.73	131.42	189.37	201.98
RS07160	rsmI	16S rRNA (cytidine(1402)-2'-O)-methyltransferase	304.17	215.12	294.67	283.58
RS07165	RS07165	DNA replication initiation control protein YabA	292.19	240.63	337.75	292.89
RS07170	holB	DNA polymerase III subunit delta'	249.02	241.98	318.61	300.95
RS07175	RS07175	cyclic-di-AMP receptor	145.43	158.38	161.81	107.65
RS07180	tmk	dTMP kinase	268.74	744.37	594.51	466.14
RS07185	recR	recombination mediator RecR	569.47	635.27	788.22	800.38
RS07190	RS07190	YbaB/EbfC family nucleoid-associated protein	749.77	727.64	1098.11	889.42
RS07195	dnaX	DNA polymerase III subunit gamma/tau	208.37	306.97	472.45	446.39
RS07200	RS07200	nucleoside deaminase	23.06	30.68	40.05	22.13
RS07205	RS07205	class I SAM-dependent methyltransferase	48.43	98.54	110.83	64.97
RS07210	nrdH	glutaredoxin-like protein NrdH	875.04	3000.16	1303.06	1591.55
RS07215	nrdE	class 1b ribonucleoside-diphosphate reductase subunit alpha	206.5	586.85	365.66	646.58
RS07220	nrdF	class 1b ribonucleoside-diphosphate reductase subunit beta	94.58	165.7	139.14	214.09
RS07225	rplL	50S ribosomal protein L7/L12	776.12	750.86	1957.61	1486.79
RS07230	rplJ	50S ribosomal protein L10	1304.95	1302.05	3577.18	4217.37
RS07235	rplA	50S ribosomal protein L1	699.84	613.87	1337.43	871.11
RS07240	rplK	50S ribosomal protein L11	2130.03	1310.48	3698.69	2450.94
RS07245	nusG	transcription termination/antitermination protein NusG	59.22	11.86	76.4	29.98
RS07255	rpmG	50S ribosomal protein L33	3590.12	6013.19	5765.05	3626.76
RS07260	RS07260	competence protein ComX	8.25	3.32	8.61	11.47
RS07265	rlmB	23S rRNA (guanosine(2251)-2'-O)-methyltransferase RlmB	169.83	148.96	267.55	313.75
RS07270	RS07270	Mini-ribonuclease 3	110.6	102.95	177.04	190.12
RS07275	cysS	cysteine--tRNA ligase	76.86	77.74	167.06	158.85
RS07280	gltX	glutamate--tRNA ligase	218.79	255.46	399.28	254.92
RS07285	RS07285	PIN/TRAM domain-containing protein	164.21	173.95	160.78	133.73
RS07290	radA	DNA repair protein RadA	143.59	198.43	172.31	134.47
RS07295	RS07295	dUTP diphosphatase	126.42	98.49	126.27	88.31
RS07300	RS07300	N-acetyltransferase	190.77	206.93	301.62	176.1
RS07305	rpiA	ribose-5-phosphate isomerase RpiA	45.79	25.62	44.95	27.34
RS07310	RS07310	C1 family peptidase	402.61	676.86	716	342.39
RS07315	RS07315	helix-turn-helix transcriptional regulator	239.2	259.61	309.97	405.9
RS07320	RS07320	MFS transporter	14.12	37.15	31.55	40.62
**RS07325**	**RS07325**	**2%2C3-bisphosphoglycerate-dependent phosphoglycerate mutase**	**62.63**	**11.13**	**19.28**	**20.81**
RS07330	RS07330	ketoacyl-ACP synthase III	36.95	116.37	80.16	110.09
RS07335	RS07335	hypothetical protein	137.59	185.69	211.78	124.07
**RS07340**	**RS07340**	**PTS sugar transporter subunit IIB**	**97.44**	**40.38**	**89.31**	**54.01**
RS07345	RS07345	sigma 54-interacting transcriptional regulator	76.36	51.34	52.69	49.84
RS07350	RS07350	DUF956 family protein	156.18	73.58	106.98	74.62
**RS07355**	**RS07355**	**PTS system mannose/fructose/sorbose family transporter subunit IID**	**1221.43**	**485.46**	**761.22**	**247.73**
RS07360	RS07360	PTS mannose/fructose/sorbose transporter subunit IIC	631.19	276.47	407.14	132.94
RS07365	RS07365	mannose/fructose/sorbose PTS transporter subunit IIA	1877.41	980.05	1388.78	537.1
RS07375	RS07375	SprT family protein	44.31	95.34	92.78	317.96

RS07380	RS07380	RNA-binding transcriptional accessory protein	46.44	114.66	151.82	351.15
RS07385	RS07385	hypothetical protein	64.95	358.38	223.57	258
RS07390	RS07390	cation-translocating P-type ATPase	59.73	375.82	286.82	345.05
RS07395	nadE	ammonia-dependent NAD(+) synthetase	386.5	232.71	244.79	258.09
RS07400	RS07400	nicotinate phosphoribosyltransferase	198.72	134.18	144.29	105.94
RS07405	RS07405	WecB/TagA/CpsF family glycosyltransferase	21.23	55.4	91.44	112.43
RS07410	RS07410	GntR family transcriptional regulator	241.49	166.54	293.48	319.41
RS07415	nagA	N-acetylglucosamine-6-phosphate deacetylase	369.48	228.7	428.99	478.34
RS07420	proC	pyrroline-5-carboxylate reductase	137.97	96.88	249.45	176.71
RS07435	RS07435	metallophosphoesterase	11.79	20.18	51.86	13.07
RS07440	RS07440	dihydrofolate reductase family protein	11.54	8.3	37.2	15.54
RS07445	RS07445	peptide MFS transporter	59.34	208.56	231.4	128.38
RS07485	lysS	lysine--tRNA ligase	285.96	281.7	573.58	387.03
RS07490	dusB	tRNA dihydrouridine synthase DusB	113.6	149.5	297.97	184.6
RS07495	hslO	Hsp33 family molecular chaperone HslO	78.72	73.14	88.86	80.87
RS07500	ftsH	ATP-dependent zinc metalloprotease FtsH	977.26	523.18	356.32	225.92
RS07505	hpt	hypoxanthine phosphoribosyltransferase	237.8	138.46	198.08	148.52
RS07510	tilS	tRNA lysidine(34) synthetase TilS	45.73	50.1	38.7	26.3
RS07515	RS07515	S1 domain-containing RNA-binding protein	71.04	91.64	83.39	51.29
RS07520	RS07520	septum formation initiator family protein	600.66	566.1	376.54	335.65
RS07525	RS07525	RNA-binding S4 domain-containing protein	122.17	44.12	81.99	96.52
RS07530	RS07530	polysaccharide biosynthesis protein	100.74	80.25	106.14	127.91
RS07535	mfd	transcription-repair coupling factor	110.55	61.49	93.22	114.45
RS07540	pth	aminoacyl-tRNA hydrolase	70.5	24.4	63.58	54.42
**RS07545**	**RS07545**	**L-lactate dehydrogenase**	**2594.33**	**1054.82**	**2594.22**	**906.92**
RS07550	RS07550	L%2CD-transpeptidase/peptidoglycan binding protein	49.9	61.56	84.79	32.57
RS07555	cbpA	cyclic di-AMP binding protein CbpA	490.86	342.32	345.96	143.31
RS07560	RS07560	type II toxin-antitoxin system PemK/MazF family toxin	179.61	157.16	172.31	116.42
RS07565	RS07565	hypothetical protein	326.44	509.53	462.69	273.12
RS07570	alr	alanine racemase	130.53	117.78	165.64	136.8
RS07575	acpS	holo-ACP synthase	478.43	406.4	572.63	612
RS07580	RS07580	DEAD/DEAH box helicase	42.54	261.64	672.6	444.71
RS07585	RS07585	UDP-N-acetylmuramoyl-tripeptide--D-alanyl-D-alanine ligase	99.98	118.66	179.47	182.81
RS07590	RS07590	hypothetical protein	468.94	459.13	649.37	752.29
RS07595	htpX	zinc metalloprotease HtpX	373.21	388.58	583.88	476.77
RS07600	RS07600	LemA family protein	202.23	243.29	295.26	283.32
RS07605	RS07605	class A sortase	19.76	56.55	92.63	61.1
RS07610	RS07610	multidrug efflux MFS transporter	39.67	93.72	201.81	188.85
RS07615	RS07615	type B 50S ribosomal protein L31	2812.46	4788.05	7421.11	3809.24
RS07620	rho	transcription termination factor Rho	80.97	109.11	231.28	286.34
**RS07625**	**RS07625**	**UDP-N-acetylglucosamine 1-carboxyvinyltransferase**	**90.16**	**163.11**	**434.36**	**285.13**
RS07630	RS07630	CTP synthase	346.44	91.37	372.06	439.3
RS07635	rpoE	DNA-directed RNA polymerase subunit delta	292.02	263.46	403.84	243.4
RS07640	RS07640	DUF1934 domain-containing protein	156.55	251.04	217.55	199.41
RS07645	RS07645	HD domain-containing protein	71.37	138.86	131.61	79.35
RS07650	yidA	sugar-phosphatase	124.31	197.08	211.31	167.5
RS07655	RS07655	DUF3899 domain-containing protein	16.16	20.32	40.9	55.06
RS07660	RS07660	ROK family protein	187.14	86.21	78.35	45.68
RS07665	RS07665	hypothetical protein	179.26	163.69	553.25	746.75

RS07670	RS07670	hypothetical protein	80.71	106.68	78.54	270.18
RS07675	RS07675	MATE family efflux transporter	21.32	52.08	69.11	158.59
RS07680	RS07680	CPBP family intramembrane metalloprotease	8.91	11.67	21.35	29.91
RS07685	RS07685	hypothetical protein	949.37	165.95	565.51	195.8
RS07690	RS07690	HAD family hydrolase	127.93	166.62	187.59	217.4
RS07695	RS07695	peptide ABC transporter substrate-binding protein	874.63	1404.76	526.76	529.49
RS07700	RS07700	FAD/NAD(P)-binding domain-containing protein	191.06	127.76	142.47	77.35
RS07705	RS07705	YitT family protein	138.04	247.19	265.72	102.3
RS07710	dltD	D-alanyl-lipoteichoic acid biosynthesis protein DltD	810.34	1359.33	1749.96	830.63
RS07715	dltC	D-alanine--poly(phosphoribitol) ligase subunit DltC	1159.64	1814.88	2043.63	878.97
RS07720	dltB	D-alanyl-lipoteichoic acid biosynthesis protein DltB	393.38	626.77	704.71	306.28
RS07725	dltA	D-alanine--poly(phosphoribitol) ligase subunit DltA	807.81	1272.32	1655.18	616.46
RS07730	RS07730	teichoic acid D-Ala incorporation-associated protein DltX	4291.51	9074.99	8880.95	6266.69
RS07735	RS07735	biotin transporter BioY	48.78	65.61	102.36	99.99
RS07740	RS07740	ECF transporter S component	41.57	157.39	127.76	81.95
RS07745	RS07745	MerR family transcriptional regulator	146.87	23.06	54.91	65.47
RS07750	RS07750	multidrug efflux MFS transporter	47.96	34.31	69.53	40.31
RS07755	RS07755	DUF4811 domain-containing protein	29.93	23.45	41.19	25.65
RS07790	RS07790	CDP-glycerol glycerophosphotransferase family protein	16.21	11.97	32.58	24
RS07795	RS07795	serine hydrolase	48.58	48.23	157.97	101.55
RS07800	RS07800	hypothetical protein	26.23	21.66	86.95	37.32
**RS07805**	**RS07805**	**ABC transporter ATP-binding protein**	**89.16**	**201.82**	**413.51**	**322.32**
**RS07810**	**RS07810**	**ABC transporter permease**	**27.28**	**46.73**	**170.19**	**64.76**
RS07820	RS07820	hypothetical protein	22.19	27.39	53.82	37.15
RS07825	RS07825	glycosyltransferase family 2 protein	124.04	109.28	224.17	140.41
RS07830	RS07830	GtrA family protein	4.62	3.3	20.08	20.28
RS07835	ligA	NAD-dependent DNA ligase LigA	211.06	33.36	70.83	46.9
RS07840	RS07840	exodeoxyribonuclease III	35.63	16.37	21.42	12.5
RS07845	RS07845	YbhB/YbcL family Raf kinase inhibitor-like protein	145.09	32.94	70.48	54.57
RS07850	RS07850	hypothetical protein	7.78	5.54	8.48	9.69
RS07855	RS07855	AEC family transporter	70.27	45.76	86.42	61.43
RS07860	RS07860	NAD-dependent malic enzyme	43.52	21.12	44.91	28.17
RS07865	RS07865	LysR family transcriptional regulator	24.84	21.96	35.74	30.84
RS07870	RS07870	FAD-dependent oxidoreductase	9202.93	2121.79	2712.05	1116.02
RS07875	RS07875	aldo/keto reductase	155.1	432.3	362.04	446.62
RS07880	RS07880	HAD-IA family hydrolase	29.55	12.63	32.54	27.2
RS07885	RS07885	ClC family H(+)/Cl(-) exchange transporter	15	11.43	39.96	23.96
RS07890	glpK	glycerol kinase GlpK	221.08	539.43	177.46	565.05
RS07895	glpO	type 1 glycerol-3-phosphate oxidase	142.23	115.48	77.35	148.75
RS07900	RS07900	aquaporin family protein	208.3	134.77	142.53	212.67
RS07905	RS07905	YfcC family protein	99.93	19.71	47.67	29.94
RS07910	arcD	arginine-ornithine antiporter	988.86	86.6	190.8	66.39
RS07915	RS07915	basic amino acid/polyamine antiporter	1608.27	64.99	256.43	37.66
**RS07920**	**arcA**	**arginine deiminase**	**5988.79**	**418.72**	**915.6**	**119.6**
**RS07925**	**arcC**	**carbamate kinase**	**11650.31**	**452.55**	**1433.25**	**139.41**
**RS07930**	**argF**	**ornithine carbamoyltransferase**	**6681.71**	**192.52**	**782.11**	**61.69**
RS07935	RS07935	DNA-binding domain-containing protein	47.94	466.27	293.96	122.45
RS07940	pepF	oligoendopeptidase F	244.85	127.94	177.61	150.2
RS07945	RS07945	hypothetical protein	62.98	29.55	55.26	62.26

RS07950	RS07950	MFS transporter	358.64	288.83	442.48	376.89
RS07955	RS07955	biotin--[acetyl-CoA-carboxylase] ligase	58.87	29.42	69.95	54.61
RS07960	RS07960	biotin transporter BioY	23.07	10.7	34.02	28
RS07965	RS07965	S-ribosylhomocysteine lyase	2053.33	162.47	534.14	155.68
RS07970	RS07970	BCCT family transporter	249.94	13.32	45.69	18.2
RS07975	RS07975	sugar O-acetyltransferase	191.28	81.91	113.52	81.44
RS07980	RS07980	FAD-dependent oxidoreductase	63.58	42.92	53.84	38.97
RS07985	RS07985	EAL domain-containing protein	52.6	124.47	302.92	152.34
RS07990	RS07990	Hsp20/alpha crystallin family protein	1887.93	839.88	384.55	637.01
RS07995	ndk	nucleoside-diphosphate kinase	319.25	1025.58	456.18	365.41
RS08000	RS08000	copper-translocating P-type ATPase	60.72	446.24	131.39	80.36
RS08005	RS08005	CopY/TcrY family copper transport repressor	85.02	522.41	224.38	104.42
RS08010	RS08010	DMT family transporter	32.93	44.2	159.03	148.52
RS08015	RS08015	winged helix DNA-binding protein	126.21	83.31	375.5	327.14
RS08020	RS08020	ATP-binding cassette domain-containing protein	24.33	174.59	466.29	400.44
RS08025	RS08025	ABC transporter ATP-binding protein/permease	142.83	213.87	260.18	183.1
RS08030	RS08030	HAD-IC family P-type ATPase	91.71	110.48	124.72	80.61
**RS08035**	**RS08035**	**ABC transporter permease**	**83.55**	**166.37**	**231.42**	**183.07**
**RS08040**	**RS08040**	**osmoprotectant ABC transporter substrate-binding protein**	**62.78**	**147.81**	**197.96**	**135.8**
**RS08045**	**RS08045**	**ABC transporter permease**	**41.08**	**96.14**	**117.98**	**75.54**
**RS08050**	**RS08050**	**ABC transporter ATP-binding protein**	**80.17**	**193.93**	**306.62**	**189.38**
RS08055	pepT	peptidase T	770.16	525.51	877.61	356.73
RS08060	RS08060	oxidoreductase	43.09	33.7	41.73	28.46
RS08065	RS08065	ABC transporter ATP-binding protein/permease	92.38	18.76	42.44	29.11
RS08070	RS08070	MarR family transcriptional regulator	133.61	12.89	44.79	23.67
RS08075	dhaM	dihydroxyacetone kinase phosphoryl donor subunit DhaM	1365.51	689.81	914.86	614.45
RS08080	dhaL	dihydroxyacetone kinase subunit DhaL	873.22	426.37	547.18	446.04
RS08085	dhaK	dihydroxyacetone kinase subunit DhaK	475.29	310.52	347.05	232.02
RS08090	RS08090	GTP pyrophosphokinase	36.83	39.66	66.83	27.99
RS08095	RS08095	hypothetical protein	52.73	41.57	65.4	22.91
RS08100	RS08100	NAD(P)H-dependent oxidoreductase	77.87	37.49	87.06	40.96
RS08105	RS08105	Asp23/Gls24 family envelope stress response protein	5001.19	7476.37	4043.58	3663.26
RS08110	RS08110	Asp23/Gls24 family envelope stress response protein	1518.44	2042.13	1091.85	943.65
RS08115	RS08115	hypothetical protein	8226.53	11806.24	5124.23	7239.81
RS08120	amaP	alkaline shock response membrane anchor protein AmaP	5465.17	8118.96	4196.47	3765.33
RS08125	RS08125	GlsB/YeaQ/YmgE family stress response membrane protein	14578.39	18776.93	9891.2	9532.18
RS08130	RS08130	HAD-IIB family hydrolase	214.27	121.15	280.23	119.29
RS08135	pyrF	orotidine-5'-phosphate decarboxylase	1427.08	581.91	734.7	239.02
RS08140	RS08140	peptide deformylase	172.63	47.89	88.32	35.14
RS08145	RS08145	GNAT family N-acetyltransferase	21.95	10.37	22.38	18.42
RS08150	RS08150	MarR family transcriptional regulator	69.27	45.79	65.68	56.09
RS08155	RS08155	TetR/AcrR family transcriptional regulator	14.57	10.97	28.79	19.8
RS08160	RS08160	ABC transporter permease	9.14	7.69	15.16	12.96
RS08165	RS08165	ABC transporter ATP-binding protein	13.2	4.96	12.51	12.09
RS08170	RS08170	carboxymuconolactone decarboxylase family protein	150.99	42.64	62.33	46.16
RS08175	RS08175	cupin domain-containing protein	102.18	22.11	37.26	30.5
RS08180	RS08180	flavodoxin	47.45	33.61	50.86	27.82
RS08185	RS08185	LysR family transcriptional regulator	95.97	38.02	81.09	22.18
RS08190	RS08190	GNAT family N-acetyltransferase	287.93	144.85	274.53	149.07

RS08195	RS08195	multidrug effflux MFS transporter	46.06	29.57	87.26	44.96
RS08200	RS08200	hypothetical protein	31.07	46.24	16.47	26.72
RS08205	larE	ATP-dependent sacrificial sulfur transferase LarE	23.99	19.98	12.19	13.77
RS08210	RS08210	aquaporin family protein	14.24	5.99	8.65	9.62
RS08215	larC	nickel pincer cofactor biosynthesis protein LarC	12.41	7.3	6.84	7.36
RS08220	larB	nickel pincer cofactor biosynthesis protein LarB	19.24	12.61	11.12	10.16
RS08225	larA	nickel-dependent lactate racemase	12.94	8.21	6.16	6.58
RS08230	RS08230	Crp/Fnr family transcriptional regulator	4.34	4.24	7.62	5.13
RS08235	RS08235	HoxN/HupN/NixA family nickel/cobalt transporter	9.78	19.56	18.94	23.31
RS08240	RS08240	GRP family sugar transporter	29.33	37.85	69.99	72.58
RS08245	rbsD	D-ribose pyranase	15.6	14.29	30.46	29.3
RS08250	rbsK	ribokinase	12.58	11.16	35.09	20.02
RS08255	RS08255	substrate-binding domain-containing protein	51.38	29.76	45.03	35.47
RS08260	RS08260	Nramp family divalent metal transporter	46.9	80.68	218.58	64.38
RS08265	RS08265	ABC transporter permease	12.76	14.08	22.7	32.1
RS08270	RS08270	glycosyltransferase	13.81	9.2	17.64	17.68
RS08275	RS08275	bifunctional glycosyltransferase family 2/GtrA family protein	11.45	6.49	15.12	18.93
RS08280	RS08280	histidine phosphatase family protein	194.49	110.63	183.35	75.28
RS08285	RS08285	AP2 domain-containing protein	142.63	64.73	94.54	96.12
RS08290	RS08290	recombinase family protein	5.89	6.96	10.06	94.36
RS08295	RS08295	SDR family oxidoreductase	673.77	469.17	537.5	308.35
RS08300	RS08300	phenylalanine--tRNA ligase beta subunit-related protein	118.18	89.91	132.1	220.71
RS08305	RS08305	2%2C3-diphosphoglycerate-dependent phosphoglycerate mutase	1430.81	582.62	1761.66	585.95
RS08310	RS08310	hypothetical protein	344.93	441.26	180.68	144.81
RS08315	RS08315	cyclopropane-fatty-acyl-phospholipid synthase family protein	122.17	536.26	1011.71	759.42
RS08320	RS08320	AzlD domain-containing protein	82.35	188.96	351.96	291.68
RS08325	RS08325	AzlC family ABC transporter permease	40.95	87.67	194.98	120.51
RS08330	RS08330	D-alanyl-D-alanine carboxypeptidase	63.58	93.24	170.38	228.1
RS08335	RS08335	HAMP domain-containing histidine kinase	146.74	131.36	151.42	162.14
RS08340	RS08340	response regulator transcription factor	173.62	111.47	148.73	165.53
RS08345	RS08345	methylated-DNA--[protein]-cysteine S-methyltransferase	92.86	51.28	141.89	220.52
RS08360	ychF	redox-regulated ATPase YchF	197.93	98.22	169.4	82.92
RS08365	RS08365	DUF951 domain-containing protein	48.9	63.49	87.57	67.99
RS08370	RS08370	ParB/RepB/Spo0J family partition protein	43.81	75.54	95.57	69.84
RS08375	rsmG	16S rRNA (guanine(527)-N(7))-methyltransferase RsmG	28.36	53.09	67.25	35.28
RS08380	RS08380	GNAT family N-acetyltransferase	5.09	29.15	127.97	85.61
RS08385	rihC	ribonucleoside hydrolase RihC	478.91	180.07	456.59	160.25
RS08390	RS08390	nucleoside transporter	275.01	127.28	368.19	92.47
RS08395	RS08395	ATP-dependent DNA helicase	23.96	15.1	43.67	27.78
RS08400	RS08400	NlpC/P60 family protein	4.47	2.83	7.79	26.2
RS08405	RS08405	MerR family transcriptional regulator	111.72	67.19	114.15	57.28
RS08410	RS08410	multidrug effflux MFS transporter	30.24	27.3	88.44	62
RS08415	rlmD	23S rRNA (uracil(1939)-C(5))-methyltransferase RlmD	42.74	99.62	199.51	90.76
RS08420	RS08420	response regulator transcription factor	12.67	6.09	15.81	12.49
RS08425	RS08425	histidine kinase	34.81	20.63	48.39	36.27
RS08430	RS08430	ABC transporter permease	18.45	12.06	29.05	32.09
RS08435	RS08435	ABC transporter ATP-binding protein	8.37	4.26	10.64	11.39
RS08440	RS08440	DedA family protein	39.92	799.53	439.87	422.55
RS08445	RS08445	hypothetical protein	70.99	322.09	178.14	173.71

RS08450	RS08450	hypothetical protein	159.9	257.43	164.81	214.68
RS08455	RS08455	acetyltransferase	31.86	38.37	150.34	55.66
RS08460	RS08460	TIGR00730 family Rossman fold protein	52.35	49.19	184.73	164.58
RS08465	RS08465	hypothetical protein	10.87	9.9	22.88	13.61
RS08470	RS08470	ABC transporter ATP-binding protein	38.53	42.4	177.67	121.92
RS08475	RS08475	FtsX-like permease family protein	33.84	32.34	94.31	99.73
**RS08480**	**RS08480**	**D-serine ammonia-lyase**	**243.87**	**39.45**	**75.08**	**31.11**
RS08485	RS08485	DNA-3-methyladenine glycosylase	245.95	42.69	122.23	59.18
RS08490	RS08490	C69 family dipeptidase	1288.93	295.26	971.42	263.22
RS08500	RS08500	hypothetical protein	30078.88	2193.39	9281.98	2721.06
RS08505	RS08505	DUF2922 domain-containing protein	22.15	16.62	30.46	29.48
RS08510	RS08510	hypothetical protein	12.25	6.71	11.66	11.24
RS08515	RS08515	hypothetical protein	116.7	287.93	805.2	410.06
RS08520	RS08520	MarR family winged helix-turn-helix transcriptional regulator	93.45	112.6	197.51	131.69
RS08525	nagA	N-acetylglucosamine-6-phosphate deacetylase	68.46	22.3	49.21	28.67
RS08530	RS08530	SIS domain-containing protein	96.02	29.1	73.29	39.6
RS08535	RS08535	GntR family transcriptional regulator	37.56	13.84	33.76	19.91
RS08540	RS08540	PTS system mannose/fructose/sorbose family transporter subunit IID	14.53	5.36	12.53	10.91
**RS08545**	**RS08545**	**PTS sugar transporter subunit IIC**	**16.87**	**6.04**	**12.76**	**15.96**
RS08550	RS08550	PTS sugar transporter subunit IIB	18.87	5.77	15.92	16.02
RS08555	RS08555	hypothetical protein	14.05	5.06	10.1	11.96
**RS08560**	**RS08560**	**PTS sugar transporter subunit IIA**	**34.59**	**8.01**	**16.28**	**16.75**
RS08565	RS08565	sialate O-acetylesterase	157.43	46.23	104.86	65.96
RS08570	RS08570	aldose 1-epimerase family protein	275.01	55.95	115.27	49.84
RS08575	RS08575	QueT transporter family protein	40.37	153.6	495.53	205.52
RS08580	RS08580	hypothetical protein	160.64	1223.3	534.73	567.69
RS08585	RS08585	zinc-binding alcohol dehydrogenase family protein	103.41	82.97	146.13	131.76
RS08590	RS08590	Rrf2 family transcriptional regulator	23.16	29.36	94.25	48.78
RS08595	RS08595	SDR family oxidoreductase	24.91	37.56	97.98	57.26
RS08600	RS08600	DUF3923 family protein	71.46	21.94	48.37	69.69
**RS08605**	**lpdA**	**dihydrolipoyl dehydrogenase**	**40.98**	**14.83**	**27.97**	**23.33**
**RS08610**	**RS08610**	**2-oxo acid dehydrogenase subunit E2**	**46.15**	**10.92**	**31.87**	**23.31**
**RS08615**	**RS08615**	**alpha-ketoacid dehydrogenase subunit beta**	**71.65**	**14.61**	**48.89**	**37.32**
**RS08620**	**RS08620**	**thiamine pyrophosphate-dependent dehydrogenase E1 component subunit alpha**	**113.78**	**20.41**	**59.2**	**53.03**
RS08625	RS08625	lipoate--protein ligase	70.69	42.46	43.24	60.55
**RS08630**	**RS08630**	**PTS transporter subunit EIIC**	**35.11**	**10.02**	**21.52**	**24.37**
RS08635	RS08635	glycoside hydrolase family 1 protein	44.68	12.24	21.55	20.27
RS08640	RS08640	GntR family transcriptional regulator	33.76	43.11	49.28	24.71
RS08645	RS08645	nitroreductase	50.08	47.26	126.85	107.28
RS08650	RS08650	DUF4767 domain-containing protein	209.12	249.22	341.76	137.43
RS08655	RS08655	YhgE/Pip family protein	37.54	30.26	77.44	74.61
RS08660	RS08660	metalloregulator ArsR/SmtB family transcription factor	36.51	39.92	99.77	104.99
RS08665	RS08665	DUF1836 domain-containing protein	57.58	52.87	109.1	55.15
RS08670	RS08670	DegV family protein	58.8	52.71	94.46	40.69
RS08675	RS08675	methyltransferase domain-containing protein	35.7	66.59	100.69	43.88
RS08680	RS08680	DUF2207 domain-containing protein	42.46	58.75	92.38	107.59
RS08690	RS08690	manganese catalase family protein	106.63	1186.14	998.02	1343.85

RS08695	RS08695	EAL domain-containing protein	138.48	147.62	288.33	206.77
RS08700	RS08700	GGDEF domain-containing protein	55.03	58.46	138.32	64.41
RS08705	RS08705	MFS transporter	12.63	37.31	118.16	58.44
RS08710	RS08710	trypsin-like peptidase domain-containing protein	321.47	219	302.46	377.97
RS08715	RS08715	MBL fold metallo-hydrolase	107.08	56.52	98.37	91.17
RS08720	RS08720	two-component system regulatory protein YycI	98.96	64.76	119.12	66.53
RS08725	yycH	two-component system activity regulator YycH	91.05	48.49	88.97	46.28
**RS08730**	**walK**	**cell wall metabolism sensor histidine kinase WalK**	**48.21**	**13.95**	**35.38**	**20.58**
**RS08735**	**yycF**	**response regulator YycF**	**204.61**	**78.24**	**90.96**	**46.37**
RS08745	RS08745	nuclear transport factor 2 family protein	38.61	23.56	57.05	35.55
RS08750	RS08750	LysR family transcriptional regulator	52.74	25.71	61.95	39.54
RS08755	RS08755	NAD(P)H-binding protein	22.25	53.41	73.06	75.96
RS08760	RS08760	MFS transporter	16.82	21.43	32.27	28.31
RS08765	RS08765	Cof-type HAD-IIB family hydrolase	17.58	13.76	37.54	27.96
RS08770	RS08770	PTS glucose transporter subunit IIABC	12.86	10.35	24.31	18.8
RS08775	treR	trehalose operon repressor	8.87	6.1	19.88	15.57
RS08780	treC	alpha%2Calpha-phosphotrehalase	10	7.51	15.76	14.96
RS08785	RS08785	hypothetical protein	8.25	8.27	14.27	15.14
RS08795	RS08795	sodium:proton antiporter	40.2	35.52	79.7	46.47
RS08800	RS08800	zinc ribbon domain-containing protein	36.02	182.61	57.62	58.35
RS08805	mscL	large-conductance mechanosensitive channel protein MscL	38.08	25.1	29.55	23.28
RS08810	RS08810	ABC transporter ATP-binding protein/permease	9.34	8.08	14.73	15.33
RS08815	RS08815	MarR family transcriptional regulator	17.43	24.16	33.48	42.46
RS08820	RS08820	hypothetical protein	28.46	10.44	24.23	26.03
RS08825	nagE	N-acetylglucosamine-specific PTS transporter subunit IIBC	11.12	17.29	53.29	59.19
RS08830	ribH	6%2C7-dimethyl-8-ribityllumazine synthase	21.09	14.34	38.86	103.98
RS08835	RS08835	bifunctional 3%2C4-dihydroxy-2-butanone-4-phosphate synthase/GTP cyclohydrolase II	17.01	9.64	22.37	44
RS08840	RS08840	riboflavin synthase	13.29	7.03	22.73	34.67
RS08845	ribD	bifunctional diaminohydroxyphosphoribosylaminopyrimidine deaminase/5-amino-6-(5-phosphoribosylamino)uracil reductase RibD	22.96	22.75	56.61	75.88
RS08850	RS08850	NAD-dependent succinate-semialdehyde dehydrogenase	131.84	506.4	221.34	182.45
RS08855	RS08855	acetate kinase	83.35	163.67	87.09	79.7
RS08860	RS08860	PadR family transcriptional regulator	12.74	74.24	60.33	41.11
RS08865	RS08865	DUF1700 domain-containing protein	17.92	46.47	39.9	31.61
RS08870	RS08870	DUF4097 domain-containing protein	8.92	27	24.31	26.42
RS08875	RS08875	LD-carboxypeptidase	52.26	61.76	96.54	68.35
RS08880	RS08880	DUF454 family protein	301.49	629.24	1042	1216.3
RS08885	RS08885	SAP domain-containing protein	28.5	13.47	21.49	29.27
RS08890	RS08890	helix-turn-helix transcriptional regulator	57.77	64.54	170.81	141.32
RS08895	RS08895	hypothetical protein	8.12	10.75	32.46	29.61
RS08900	rlmH	23S rRNA (pseudouridine(1915)-N(3))-methyltransferase RlmH	91.65	69.49	110.51	124.32
RS08905	addA	helicase-exonuclease AddAB subunit AddA	63.46	64.73	58.47	67.87
RS08910	RS08910	exodeoxyribonuclease V subunit gamma	35.05	35.13	32.61	35.8
RS08915	RS08915	NAD(P)H-binding protein	21.3	32.57	21.24	16.91
RS08920	RS08920	purine permease	7.75	157.65	209.93	63.22
RS08925	RS08925	DNA-3-methyladenine glycosylase I	110.82	48.3	140.57	104.01
RS08930	RS08930	SGNH/GDSL hydrolase family protein	221.36	278.37	339.07	304.14

RS08935	nhaC	Na+/H+ antiporter NhaC	22.75	119.52	94.42	128.87
RS08940	cls	cardiolipin synthase	32.03	28.29	46.47	54.04
RS08945	RS08945	HIT family protein	322.45	127.86	206.23	119.58
RS08950	lepB	signal peptidase I	231.82	451.11	862.31	374.66
RS08955	yaaA	peroxide stress protein YaaA	20.79	22.24	32.81	31.33
RS08960	mnmG	tRNA uridine-5-carboxymethylaminomethyl(34) synthesis enzyme MnmG	40.22	349.85	441.35	633.7
RS08965	mnmE	tRNA uridine-5-carboxymethylaminomethyl(34) synthesis GTPase MnmE	32.64	148.1	315.87	276.45
RS08970	yidC	membrane protein insertase YidC	51.36	58.08	234.67	90.92
RS08975	rnpA	ribonuclease P protein component	90.43	104.95	409.65	166.61
RS08980	rpmH	50S ribosomal protein L34	1161.53	12745.22	21239.48	14291.94
RS09025	RS09025	bifunctional glycosyltransferase family 2 protein/CDP-glycerol:glycerophosphate glycerophosphotransferase	110.25	122.26	171.8	129.71
RS09030	RS09030	hydrolase	22.71	12.77	35.53	19.19
RS09075	agaB	PTS galactosamine transporter subunit IIB	151.18	120.88	355.82	249.75
RS09105	rpmG	50S ribosomal protein L33	1119.68	2535.47	2520.03	1535.79
RS09110	RS09110	DUF3042 family protein	1995.44	1196.63	1124.95	591.82
RS09130	RS09130	YjzD family protein	2	2.31	4.63	6.02
RS09135	RS09135	DUF4044 domain-containing protein	7.81	14.01	46.69	86.45
RS09140	RS09140	A24 family peptidase	3.04	2.44	5.22	12.3
RS09155	RS09155	hypothetical protein	364.4	1121.42	826.75	790.3
RS09160	RS09160	hypothetical protein	3153.62	1800.2	732.15	469.01
RS09175	RS09175	hypothetical protein	1254.14	678.73	1072.89	548.59
RS09180	RS09180	IMP dehydrogenase	571.12	498.9	866.07	860.88
RS09185	RS09185	DUF1129 domain-containing protein	367.52	210.87	376.94	207.6
RS09225	RS09225	putative metal homeostasis protein	12.51	22.41	8.33	9.51
RS09230	RS09230	hypothetical protein	1463.42	4795.6	2141.29	3246.21
RS09235	RS09235	hypothetical protein	63.84	84.49	102.47	103.95
RS09240	RS09240	hypothetical protein	9.61	2.14	3.12	2.71
RS09250	RS09250	hypothetical protein	38540.67	36551.99	49556.72	52355.73
RS09260	RS09260	hypothetical protein	22.35	1.08	10.07	7.8
RS09285	RS09285	SPJ_0845 family protein	300.7	245.76	96.91	257.16
RS09290	RS09290	hypothetical protein	209.62	87.05	204.87	186.06
RS09305	RS09305	LPXTG cell wall anchor domain-containing protein	5.42	3.37	6.57	7.31
RS09310	RS09310	phosphoribosyltransferase family protein	39.19	100.39	137.76	337.55
RS09330	RS09330	DUF4767 domain-containing protein	34.08	32.22	36.67	45.77
RS09335	RS09335	hypothetical protein	48.46	37.54	44.16	67.8
RS09345	RS09345	hypothetical protein	15.72	6.23	2.25	9.29
RS09350	RS09350	hypothetical protein	3685.28	4365.12	2225.25	2238.64

***The genes in bold represent major differentially expressed genes**

Table S7 Detailed information on differential metabolites

Metabolites	KEGG	$\log_2$(FC)
Etoposide phosphate		36.77121081
Catechin-(4alpha->8)-gallocatechin-(4alpha->8)-gallocatechin		33.27119872
Ikarisoside D		33.11007632
Hydroxycelecoxib		32.65204248
Leucodelphinidin 3-[galactosyl-(1->4)-glucoside]		32.46317137
dTDP-alpha-D-glucose		31.9920101
Phaseol	C05229	-31.83551595
(2S,3S,4S,5R)-6-[4-(5,7-Dihydroxy-4-oxochromen-3-yl)phenoxy]-3,4,5-trihydroxyoxane-2-carboxylic acid		31.83010452
Pyridoxamine 5'-phosphate	C00647	31.6767877
2-Amino-4-oxo-6-(1',2',3'-trihydroxypropyl)-diquinoid-7,8-dihydroxypterin	C05253	31.30506063
Eruberin B		-31.13746358
Erythronic acid		31.10374878
1,1-Diphenyl-2-picrylhydrazine		23.77267498
8-Hydroxytricetin 7-glucuronide		23.00978184
2-((4-Methoxy-3-methyl-2-pyridylmethyl)sulfo)-5-trifluoromethyl-1H-benzimidazole	C15664	-19.53913135
Caylin-2		16.84696077
Viridin		14.74818001
D-Glucose, 6-O-alpha-D-glucopyranosyl-		14.10460646
3-Hydroxyphloretin 2'-O-xylosyl-glucoside		13.99738511
Negletein 6-[rhamnosyl-(1->2)-fucoside]	C10023	13.86116679
Angoroside C		12.93924092
Lykurim		11.85891477
Isopongaflavone		-11.71305254
Glycerol lactate pyruvate		11.2383362
Cer(d18:1(4E)/20:0(3OH))		10.7789828
Deserpidine	C06541	10.16709773
L-Glutamic acid 5-phosphate	C03287	-10.14853801
silvestrol		10.12228254
Cefovecin		-9.773035925
3-Hydroxy-3-carboxymethyl-adipic acid		9.586560474
Sarmentosin	C08340	-9.058690921
Valrubicin		8.936715653
Starch acetate		8.469026102
(2R,3S)-Piscidic acid		8.257966315
Ampiroxicam		-7.937780759
Cer(d18:1(4E)/19:0(3OH))		7.310223472
Plumbagin	C10387	7.002817
modafinil acid		6.994321638
2-Amino-6-(2-hydroxypropanoyl)-2,3-dihydro-1H-pteridin-4-one		6.855327648

Hypoxanthine	C00262	6.820782862
N-(4-Methylphenyl)diphenimide		6.694166755
Apigeninidin 5-(5''-caffeylarabinoside)		6.680531731
Inosine 2',3'-cyclic phosphate	C05768	6.540738085
Salacinol		6.536265286
Cefoselis		-6.38674398
4'-Hydroxy Nimesulide		6.295964516
Glucosylisomaltol		6.142380549
Hexandraside D		6.11897191
Tetrahydrofurfuryl acetate		6.10726915
N-Acetyl-L-aspartic acid	C01042	-6.048914119
2-(acetylamino)-1,5-anhydro-2-deoxy-4-O-b-D-galactopyranosyl-D-arabino-Hex-1-enitol		5.786261969
6(5H)-Phenanthridinone		-5.743231615
Fagopyritol B3		5.65250124
D-N-(Carboxyacetyl)alanine		-5.551712404
Primisulfuron-methyl		-5.545874204
beta-D-galactosyl		5.372547666
NADH pyruvate		5.372500314
Ilicifolinoside A		5.326618031
(2E,11Z)-5-[5-(Methylthio)-4-penten-2-ynyl]-2-furanacrolein		5.279294024
Methyl salicylate O-[rhamnosyl-(1->6)-glucoside]		5.205911164
(a-D-mannosyl)2-b-D-mannosyl-N-acetylglucosamine		5.106919663
5-Amino-2-p-toluidinobenzenesulfonic acid		5.097847079
Lactodifucotetraose		5.007502563
Heptafluorobutyric anhydride		-4.999259082
16,17-Dihydro-16alpha,17-dihydroxygibberellin A4 17-glucoside		4.901849252
2-Hydroxyadipic acid	C02360	4.719805943
Ilaprazole		4.66795538
Desacetylvinblastine		-4.640125139
6-Benzyl-1-(benzyloxymethyl)-5-iodouracil		4.639538772
Imetit	C17931	4.636474523
Mikanin 3-O-sulfate		-4.499912387
Pyroglutamyl-prolyl-arginine-4-nitroanilide		4.406260351
Sucrose octaacetate		4.324694914
FAPy-adenine	C06502	4.244756739
Cyclo(-D-Tyr-Arg-Gly-Asp-Cys(carboxymethyl)-OH) sulfoxide		4.211308469
N(6),O(2)-Dimethyladenosine		3.973799098
Casopitant		3.948597513
3,5,6-Trihydroxy-5-(hydroxymethyl)-2-methoxy-2-cyclohexen-1-one		3.897956193
1-[(5-Amino-5-carboxypentyl)amino]-1-deoxyfructose		3.883849059
N-methyl Leukotriene C4		3.873336434
Guanine	C00242	3.782590254
Citbismine F		3.770978893

Mumefural		3.686206343
ascr#11		3.637227099
Morin-5'-sulfonic acid		-3.61068098
Deoxyadenosine	C00559	3.54798672
H-9		3.539382506
PS(22:6(4Z,7Z,10Z,13Z,16Z,19Z)/0:0)		3.51441702
24-hydroxy-10Z-tetracosenoic acid		3.483133052
CDP-DG(i-22:0/18:2(9Z,11Z))		-3.348605443
Trigoneoside VIII		-3.348025628
Bilobetin		3.299773091
phosphonoacetaldehyde	C03167	3.27633648
POB-PS		-3.267285656
Osmaronin		3.259344619
Deltorphin B	C18095	-3.253841635
Pirbuterol	C07807	-3.205239727
Theobromine	C07480	3.181737098
Abacavir	C07624	-3.181715305
PG(22:6(4Z,7Z,10Z,13Z,16Z,19Z)/20:5(7Z,9Z,11E,13E,17Z)-3OH(5,6,15))		-3.17033986
Alkergot		3.169867069
Threonylglutamic acid		-3.157223754
1-O-(2R-hydroxy-4Z-heicosenyl)-sn-glycerol		3.124421826
Uridine diphosphate-N-acetylglucosamine	C00043	3.106136852
N-methyl-4,6,7-trihydroxy-1,2,3,4-tetrahydroisoquinoline		-3.101468012
PIP(20:0/18:2(9Z,11E)+=O(13))		-3.099801044
(E)-4-(trimethylammonio)but-2-enoyl-CoA		3.087279583
Serylglutamine		3.049696521
11-keto-Octacosanoic acid		3.03614306
2-[4-(Dimethylamino)phenyl]benzothiazole-6-ol		-3.016411869
1-Butyl-3-(pyridine-4-carbonylamino)thiourea		3.011131964
Verbascoside		2.980712571
CDP-DG(i-19:0/18:1(12Z)-2OH(9,10))		-2.931723359
Deoxyguanosine	C00330	2.917392241
UDP-N-acetylmuraminate	C01050	2.889959696
Serylleucine		-2.845583023
Ornithine	C00077	-2.829050981
5-(3-(4-(2-(4-Fluorophenyl)ethoxy)phenyl)propyl)furan-2-carboxylic acid		2.82815824
20-CoA-20-oxo-18R-hydroxyleucotriene B4		2.80137908
Neocasomorphin (1-5)		-2.780243428
L-Fucose	C01019	2.749106126
Erythromycin C	C06616	-2.747143252
3'-Sialyl-N-acetyllactosamine		2.721014798
Leu-Arg-Asn-Arg		-2.711655957
FAHFA(18:1(9Z)/6-O-18:0)		2.684419064
Methyl 7-epi-12-hydroxyjasmonate glucoside		-2.676122713

1-O-(2R-hydroxy-4Z-nonadecenyl)-sn-glycerol		2.662505206
Adenine	C00147	2.653606544
Tezacaftor		2.648384301
Okanin 3,4,3'-trimethyl ether 4'-glucoside		2.639760195
Quercetin 5,7,3',4'-tetramethyl ether 3-rutinoside		2.594775582
xi-Linalool 3-[rhamnosyl-(1->6)-glucoside]		-2.593877843
3-Hydroxy-2-nitrobenzaldehyde		2.580212261
Ceranapril		-2.577145538
16alpha-Acetoxyasclepin		-2.572287638
3-[2-Methoxy-4-[3-methoxy-4-(2-phenyl-1H-tetrazol-3-yl)phenyl]phenyl]-2-(4-nitrophenyl)-5-phenyl-1H-tetrazole		2.561042334
Licoricesaponin G2		-2.54953673
Berotralstat		2.538609951
10Z-Nonadecenoic acid		2.529073578
2-Hydroxy-3-methylbutyric acid		2.522202251
29-Oxotriacontanoic acid		2.522057526
Dslet		-2.520910016
Phenylalanylproline		-2.519047093
Myricetin 3-glucoside		-2.512482705
N-acetyl-L-2-aminoadipate(2-)		-2.504931278
Flupenthixol		2.495622913
Asparaginylisoleucine		-2.478474453
Ginsenoside La		-2.472395825
2-Ethyldihydro-3(2H)-thiophenone		-2.441751294
Lactacystin		-2.429897038
Quilostigmine		2.421604123
(3b,9R)-5-Megastigmene-3,9-diol 9-[apiosyl-(1->6)-glucoside]		-2.409049857
Fructose lactate		-2.398471741
Zoanthone A	C08811	-2.396463841
Merodesmosine		-2.39620922
Citrusin F		2.383244601
Nicotinic acid	C00253	2.379324134
23R,acetoxy-3beta,15alpha,24R,25-tetrahydroxy-cycloart-7-en-16-one-3-O-beta-D-xylopyranoside		-2.374920855
O-Acetylserine	C00979	-2.36694475
Uracil	C00106	2.350506752
Valylalanine		-2.34760429
7-(3-((3-Acetylphenoxy)methyl)-1,5-dimethyl-1H-pyrazol-4-yl)-3-(3-(naphthalen-1-yloxy)propyl)-1H-indole-2-carboxylic acid		2.343864008
Phenyllactic acid	C01479	-2.341210826
Pyroglutamylvaline		-2.332601822
Threonylleucine		-2.327332946
Pimozide	C07566	2.322254628
CDP-DG(5-iso PGF2VI/22:6(4Z,7Z,10Z,13Z,16Z,19Z))		-2.313463623

3-iodo-octadecanoic acid		-2.307599815
Annomuricatin B		-2.27761953
Galactosyl 4-hydroxyproline		2.273394835
Lisinopril	D00362	-2.273363616
isoleucine glutamate		2.272943218
Glucosyl (2E,6E,10x)-10,11-dihydroxy-2,6-farnesadienoate		-2.271139437
Patuletin 3-(6''-p-coumaroylglucoside)		2.266533754
Astragaloside III		-2.255669774
TOG10		2.251918737
PA(22:5(7Z,10Z,13Z,16Z,19Z)/18:1(12Z)-2OH(9,10))		-2.244944047
norzolmitripan		2.244312891
2,4-Diaminoanisole	C19218	-2.243694159
D-Malic acid	C00497	-2.234473604
SM(d20:1/18:1(9Z)-O(12,13))		2.233526883
(+/-)-(E)-Ethyl-2-[(E)-hydroxyimino]-5-nitro-3-hexeneamide		2.209170004
ADP-ribose 2'-phosphate	C03246	2.208601421
Prolylproline		-2.208066962
diginatin	C08861	-2.19881847
Sitagliptin		2.194696867
Valylmethionine		-2.188854034
Diphenhydramine N-glucuronide		2.17835388
Galactosylpyridinoline		2.175313696
Hydroxyphenyllactic acid	C03672	-2.167528595
Isoleucylproline		-2.166679045
PI(22:5(4Z,7Z,10Z,13Z,19Z)-O(16,17)/18:0)		-2.155257051
Sulfolithocholylglycine	C11301	-2.123153615
Glucuronidated Sn-38		2.106646875
7-Methylguanosine		2.096454974
Galactotriose		2.087961405
7-Hydroxymethyl-12-methylbenz[a]anthracene sulfate	C19562	-2.08596086
O-phosphonato-L-homoserine(2-)	C05702	-2.076562605
25-Hydroxy-atrotosterone A		-2.055299255
2-Amino-4-ethoxy-3-hydroxybutanoic acid		2.054421194
PG(12:0/18:3(6Z,9Z,12Z))		-2.05310012
Maleic acid	C01384	-2.034053044
Vecuronium	C07553	2.025556433
Dendrodoine		2.003674698
(3-{(1r)-3-(3,4-Dimethoxyphenyl)-1-[({(2s)-1-[(2s)-2-(3,4,5-Trimethoxyphenyl)pent-4-Enoyl]piperidin-2-Yl}carbonyl)oxy]propyl}phenoxy)acetic Acid		2.000539386
Methyl reserpate		1.989336551
D-1-Deoxy-erythro-hexo-2,3-diulose		1.982255281
Cytidylyl-(3',5')-guanosine		1.979826075
Difructose anhydride III		1.977662282
Creatinine	C00791	-1.966684154

N-Carbamoyl-2-amino-2-(4-hydroxyphenyl)acetic acid		1.947607508
Pro-Ile		-1.94438253
Histidine glutamate		-1.938597213
Bz-Pro-Phe-Arg-pNA		1.930260294
N-(1-Deoxy-1-fructosyl)leucine		-1.927268598
H-Asp-ala-his-lys-OH		-1.918499224
Gly-arg-gly-asp-ser		1.911994557
3-Hydroxyhexanedioylcarnitine		-1.911923398
Cer(m18:0/16:0)		-1.908306995
6'-Apiosyllotaustralin		-1.903796972
Histidinohydroxylysinonorleucine		-1.891566747
Levan	C01355	1.887025561
Imidazolepropionic acid	C20522	1.854844034
Candoxatrilat		-1.845914132
5'-Phosphoribosyl-N-formylglycinamide	C04376	-1.844879805
PE(19:0/0:0)		1.843543131
Histidinyl-Alanine		1.842169994
Bumetanide	C06859	-1.835526168
OA-PA		1.827200031
Suprofen S-oxide		-1.822280272
O-b-D-Gal-(1->3)-O-[O-b-D-Gal-(1->4)-2-(acetylamino)-2-deoxy-b-D-Glc-(1->6)]-2-(acetylamino)-2-deoxy- D-Galactose		1.815905392
Aspidospermatine	C09041	-1.813593934
Goshonoside F3		1.809993584
L-NNA		-1.803974477
N,N-Diallyl-tyrosyl-aminoisobutyryl-aminoisobutyryl-phenylalanyl-leucine		-1.80344264
Psychosine sulfate		-1.801518679
PGP(a-13:0/PGJ2)		-1.792591765
Tyrosyl-Proline		-1.788507195
2-O-alpha-D-Galactopyranosyl-1-deoxynojirimycin		1.786515002
Glimepiride	C07669	1.765204212
1(3h)-Isobenzofuranone, 3-[4-hydroxy-2-methyl-5-(1-methylethyl)phenyl]-3-[2-methyl-5-(1-methylethyl)-4-(phosphonooxy)phenyl]-		1.739317648
(2S)-1-[(2S)-3-(4H-Imidazol-4-yl)-2-[[(2S)-5-oxopyrrolidine-2-carbonyl]amino]propanoyl]pyrrolidine-2-carboxylic acid		-1.738434845
N-Acetylhistidine	C02997	1.727142657
Tumonoic Acid H		-1.726121355
Glutamylisoleucine		-1.716714993
Etidronic acid	C07736	-1.715892502
Majoroside F1		-1.708782825
Gracilosulfate B		1.704327266
Polyribosylribitolphosphate		-1.689519415
25-Cinnamoyl-vulgaroside		1.683356746
NAD	C00003	1.681959667

N-(3-hydroxy-pentanoyl)-homoserine lactone		-1.680021515
Inosinic acid	C00130	-1.670940596
Ginsenoside Rb1		1.665133985
Melezitose	C08243	1.661484202
9Z-Pentatriacontene		-1.656879188
lithocholic acid sulfate		1.655105637
Choline sulfate	C00919	1.652433555
Thromboxane B2	C05963	-1.646184699
Nalfurafine		1.645083534
(2R,5R)-2-[6-(Cyclopentylamino)-8-(methylamino)purin-9-yl]-5-(hydroxymethyl)oxolane-3,4-diol		-1.641343216
2',3'-Didehydro-2',3'-dideoxycytidine		1.636502702
Olmesartan medoxomil		1.635690749
N1,N8-Diacetylspermidine		-1.6353551
5'-Methylthioadenosine	C00170	1.615855845
L-Agaridoxin		1.61350198
2-Hydroxy-4-trifluoromethyl benzoic acid		-1.612392781
N-(3-oxododecanoyl) homoserine lactone		-1.60898897
12-D2t-IsoP		-1.591637278
O-propanoyl-carnitine	C03017	1.591179524
3,5-Di-O-galloyl-1,4-galactarolactone		-1.580505235
3-Nitro-2-nitrooxybenzoic acid		-1.571091017
8-Epiiridodial glucoside tetraacetate	C11658	1.55909464
viloxazine	D08673	-1.557084574
4,5-dihydroxyhexanoic acid		1.555997625
Omethoate	C18662	-1.549704364
N-Oxalyl-L-alanine		1.548214856
N-Formyl-L-methionine	C03145	1.539369879
Kumatakenin		1.539248984
Berkeleylactone G		-1.538867495
9-(2,6-Dioxo-3H-purin-9-yl)-3H-purine-2,6-dione		-1.537619341
Cyclic ADP-ribose		1.536974292
Serylphenylalanine		-1.528256375
9H-Purine-9-butanoic acid, 6-amino-alpha-hydroxy-, methyl ester		-1.520035044
3-Oxotetradecanoic acid		-1.515096417
L-4-Hydroxyglutamate semialdehyde	C05938	-1.513154077
Z-PP-CHO		-1.511718367
Aspartyl-Glycine		1.50955683
Asparaginyl-Proline		-1.50558023
N-(1-Deoxy-1-fructosyl)tyrosine		-1.496890094
PKODA-PS		1.493721821
Guanosine diphosphate	C00035	-1.493187001
Neosaxitoxin	C17208	-1.482940551
icas#18		1.481667867

Hexahydropyrrolo[1,2-a]pyrazine-1,4-dione		-1.478597898
3'-Sialyllactose		1.476486253
(+/-)-3-Mercapto-1-butyl acetate		-1.475470058
PI(22:1(11Z)/0:0)		1.474155258
OKODA-PE		1.472745008
Succinic acid	C00042	-1.470899617
Benzyloxycarbonylphenylalanylphenylalanine diazomethyl ketone		1.466729858
Thymidine glycol		-1.460033199
Citric acid	C00158	-1.455616786
9Z-Tritriacontene		-1.450219427
Cellulose, microcrystalline		1.437622326
Adomeglivant		1.435683737
Hydroxyprolyl-Proline		-1.422969361
oscr#12		1.418638512
Alpha-Trisaccharide		1.417280624
Blumenol C glucoside		-1.395374182
beta-nicotinamide adenine dinucleotide		1.392413057
Sulfadoxine	C07630	1.385912076
Cyclo(L-prolyl-L-valyl)		-1.383658388
Panax ginseng Tetrapeptide		-1.38271932
3-Hydroxy-3-phenylpentanamide		-1.380562088
Lamellosterol A		1.37968418
1-Octadecene		-1.379622423
Azetirelin		-1.376090217
OKODA-PS		1.372745656
Thiopental	C07521	-1.371490692
6-Butyryl-5-hydroxy-4-phenylseselin		-1.370017551
GRK2 Inhibitor		1.368431764
Glycerophosphocholine	C00670	1.361923951
N-(1-Deoxy-1-fructosyl)phenylalanine		-1.361245455
Glutamylmethionine		-1.358490582
DG(22:6(5Z,8E,10Z,13Z,15E,19Z)-2OH(7S, 17S)/22:0/0:0)		1.356550612
Metharbital		-1.356428818
Apigenin 7-[feruloyl-(->2)-glucuronyl-(1->2)-glucuronide] 4'-glucuronide		1.345918377
Cinobufagin		1.338663912
ribostamycin	C17584	1.334990331
N-Acetyl desmethyl frovatriptan		-1.332305951
PI(12:0/12:0)		1.330850787
dolichyl beta-D-glucosyl phosphate	C01246	1.31531773
2-(Bis(2-(2,6-dioxomorpholino)ethyl)amino)acetic acid		1.308701135
Canarigenin 3-[glucosyl-(1->4)-6-deoxy-alloside]		1.308065747
Calcium citrate		1.304947626
4-(4-Fluorophenyl)-2-(4-hydroxyphenyl)-5-(4-pyridyl)imidazole		-1.303590279

Pyrazolo(1,5-a)pyrimidine-3-carbonitrile, 4,5-dihydro-4-((4-(phenylmethyl)-1-piperazinyl)acetyl)-7-(3-(trifluoromethyl)phenyl)-		1.301930263
N-Acetylthreonine		1.296637481
Cyclo(L-Phe-L-Pro)		-1.296599371
[(2R,5R)-5-(6-Aminopurin-9-yl)-4-hydroxy-2-(hydroxymethyl)oxolan-3-yl] dihydrogen phosphate		-1.293424217
Dihydrozeatin-9-N-glucoside-O-glucoside		1.285951645
gamma-Glutamylleucine		-1.277402824
EPITUMOMAB CITUXETAN		1.275447347
o-Cresolphthalein complexone		1.272828213
3,5-Dinitrosalicylic acid	C11319	-1.271084718
Bufotenine O-glucoside		1.267056845
3'-Amino-3'-deoxythimidine glucuronide		1.265128731
Cerebrocrast		1.257891511
BioMed 101		1.250103398
2-Azetidinecarboxylic acid, 3-(3-((aminoiminomethyl)amino)propyl)-1-((4-(((1,1-dimethylethyl)amino)carbonyl)-1-piperazinyl)carbonyl)-4-oxo-, (2S,3R)-		-1.2487282
Serotinose		1.248326951
Glycerol 3-phosphate	C00093	-1.244886265
1-Hydroxytrimazosin		1.243451703
3-dehydroecdysone	C02513	1.240440261
5,6-Dihydroxy-3-phenyl-1-aminomethylisochroman		-1.233389722
D-Gulose	C00738	-1.228311094
Ketanserin	C07464	1.224015514
L-Leucine	C00123	-1.220568242
Valyl-Arginine		-1.217131675
Apigenin 4'-[feruloyl-(->2)-glucuronyl-(1->2)-glucuronide] 7-glucuronide		1.21582624
1-Nitro-5-glutathionyl-6-hydroxy-5,6-dihydronaphthalene	C14806	1.209025072
Biriperone		1.207771296
Glycylhydroxyproline		1.204773562
PG(16:1(9Z)/0:0)		1.198973421
Benfuracarb	C11073	1.198246256
methionine glutamate		-1.193843897
1-(2-methoxy-5Z-hexadecenyl)-sn-glycero-3-phosphoserine		1.193055806
beta-D-Glucosyloxydestruxin B		1.190152914
1-Heptanesulfonic acid		1.189183644
Monoxerutin		1.188476922
Isocitric acid	C00311	-1.186495266
Cimoxatone		1.186376908
Noradrenochrome o-semiquinone		-1.183410476
1,4'-Bipiperidine-1'-carboxylic acid	C16836	-1.170237165
Perlolyrine	C09231	1.169021141
N-(1-Deoxy-1-fructosyl)methionine		-1.167429193
2-Hydroxy-3-methylpentanoic acid		-1.16683759

Maculosin	C10605	-1.165704475
(+)-Isopilocarpine		-1.16254015
8-Pcpt-2'-O-Me-cAMP		-1.155978558
Pentenedioic acid		-1.140489224
Prolyl-Asparagine		1.129106267
Cer(d16:0/16:0)		1.127069476
L-Homoserine	C00263	-1.120275131
Acetylhomoserine	C01077	1.112103981
DG(18:2(9Z,12Z)/16:0/0:0)		1.107909529
5,7,2',3',4'-Pentahydroxy-3,6-dimethoxyflavone 7-glucoside		1.105505133
Arginylproline		-1.097243787
cis-4-Hydroxy-D-proline	C03440	1.096486484
N-(1-Deoxy-1-fructosyl)valine		-1.095734701
Glucosaminylmuramyl-2-alanine-D-isoglutamine		1.09120339
gamma-Glutamylproline		1.088712594
Resveratrol 4'-(6-galloylglucoside)		1.086554161
Amrinone	C13594	1.079941624
3-beta-Gentiobiosylglucose		1.073781622
alpha-[3-[(Hydroxymethyl)nitrosoamino]propyl]-3-pyridinemethanol	C19580	-1.073589476
5beta-Cyprinolsulfate	C05468	1.073230119
N-Acetylglutamine		1.068710639
D-Ornithine	C00515	-1.067139774
O-Pimeloylcarnitine		1.065481833
a-L-Fucopyranosyl-(1->2)-b-D-galactopyranosyl-(1->2)-D-xylose		1.058941499
Microdiscusol G		1.053003011
Luteorin 7-(2''-feruloylglucuronosyl)-(1->2)-glucuronide-4'-glucuronide		1.05070116
Triethylamine	C14691	1.046865888
Chembl4211493		-1.040040689
2-[(3-Iodophenyl)methyl]-1-[[N'-[(3-iodophenyl)methyl]carbamimidoyl]amino]guanidine		1.039962573
Val-pro-pro		-1.03480162
(S)-N-Benzyl-2-((S)-2-(2-hydroxyacetyl)pyrrolidine-1-carbonyl)pyrrolidine-1-carboxamide		-1.024223338
2-Tert-Butyl-5-phenyl 4-[(4-piperidin-1-ylphenyl)amino]isothiazol-3(2H)-one 1,1-dioxide		1.011919488
5,9:7,10a-Dimethano-10aH-[1,3]dioxocino[6,5-d]pyrimidine-4,7,10,11,12-pentol, octahydro-12-(hydroxymethyl)-2-imino-,(4R,4aR,5R,7S,9S,10S,10aR,11S,12S)-		1.009457426
Clofazimine glucuronide		-1.007839742
2'-Deoxymugineic acid		-1.003618613
1,4,10,13-Tetraoxa-7,16-diazacyclooctadecane		-1.00235284
Acetamide, 2-((2-hydroxyethyl)thio)-N-(3-(3-(1-piperidinylmethyl)phenoxy)propyl)-		0.996326208
3,6,9,12-Tetraazatetradecanedioic acid, 3,6,9,12-tetrakis(carboxymethyl)-		0.990961938
D-Aspartic acid	C00402	0.988995268

LysoPA(8:0/0:0)		0.986528628
2-deoxy-20-hydroxy-5alpha-ecdysone 3-acetate		0.984189632
Cyclopentenyl cytosine		0.981454615
AB-MECA		0.977100455
cis-Palmitvaccenic acid		0.97406682
Pyridinoline		0.974037684
trans-4-Aminocyclohexanecarboxylic acid		0.958747655
1,5-Bis(4'-glucopyranosyloxy-3'-hydroxyphenyl)pent-4-en-1-yne		0.948201697
alpha-Acetyllysine		0.946027229
N-Lactoylleucine		-0.94148012
1-[[(2S,3S,4S,5R)-3,4-Dihydroxy-5-(hydroxymethyl)-2-[(2R,3R,4S,5S,6R)-3,4,5-trihydroxy-6-(hydroxymethyl)oxan-2-yl]oxyoxolan-2-yl]methoxy]propan-2-one		0.9407204
3,4-Dihydroxyphenylvaleric acid 4 sulfate		-0.935523978
((2R,3S)-3-Amino-2-hydroxy-2-(1H-imidazol-4-ylmethyl)-5-methyl)-5-methylhexanoic Acid		-0.934816794
Benziodarone		0.930968899
Prolyl-Histidine		-0.924256071
[(2S,4R,5R,6R,14S,16R)-14-Hydroxy-7,11-dimethyl-6-(2-oxopyran-4-yl)-3-oxapentacyclo[8.8.0.02,4.02,7.011,16]octadecan-5-yl] acetate		0.912229303
Withaminimin		0.907930194
4-(1-Hydroxy-2-methoxyethyl)-5-(hydroxymethyl)-2-methylpyridin-3-ol		-0.907775984
3-Fluoro-5-[(pyridin-3-yl)ethynyl]benzonitrile		-0.885434863
Disodium phosphate	C13558	-0.88288404
Glutaminylthreonine		-0.878318891
N-[3-[(1S)-1-[(6R)-2,4-Dioxo-6-(2-phenylethyl)-6-propyloxan-3-yl]propyl]phenyl]-5-(trifluoromethyl)pyridine-2-sulfonamide		0.873536996
CerP(d18:1/2:0)		-0.870516129
Glycylprolylarginine		0.868246766
N6,N6,N6-Trimethyl-L-lysine	C03793	0.864806867
epsilon-(Carboxymethyl)lysine		-0.864303713
beta-tyvelose		0.862001249
S-Isopropyl 3-methylbut-2-enethioate		0.85985011
(-)-2,7-Dolabelladiene-6beta,10alpha,18-triol		-0.855904772
Poly-g-D-glutamate	C05723	0.855329935
Met-enkephalinamide		0.855154877
Beta-Tyrosine	C04368	-0.853158874
Mopidamol		-0.851925803
Imidazoleacetic acid riboside	C05131	0.848311008
N2-Acetylornithine	C00437	0.842539264
2,4,6-Trimethyl-1,3,5-dioxathiane		0.841962884
(3S,4R,5R)-1,3,4,5,6-Pentahydroxy-1-morpholin-4-ylhexan-2-one		-0.840272013
Tetraphyllin B		-0.827247753
gamma-L-Glutamyl-S-(2-carboxy-1-propyl)cysteinylglycine		0.826075656
2-Hydroxymyristic acid		-0.811876234

Tetrahydrofolyl-[Glu](n)	C03541	0.811732051
2-hydroxy palmitic acid		0.810277277
Hygromycin B	C01925	0.808659205
Eicosen-1-ol		0.804190979
1-Deoxy-1-(N6-lysino)-D-fructose		-0.803142161
D-Tagatose	C00795	-0.801754975
(S1)-Methoxy-3-heptanethiol		0.789275295
L-Carnitine	C00318	0.785630835
(9Z,11E,13S,15Z)-13-Hydroxyoctadeca-9,11,15-trienoic acid		0.771920336
Aspartylphenylalanine		-0.751880922
N-acetyl-2-carboxy Benzenesulfonamide		0.750961872
Glycochenodeoxycholic acid 3-glucuronide		0.749388247
CDP-glycerol	C00513	0.743480532
Ribalinium	C10735	0.736640584
2-Hydroxycinnamic acid	C01772	-0.733909283
Digalactosyldiacylglycerol		0.732598404
Desflurane	C07519	0.727026519
Vidarabine	C07195	0.721763378
11-beta-Hydroxyandrosterone-3-glucuronide	C05643	0.716506579
PGP(20:3(8Z,11Z,14Z)-O(5,6)/18:0)		0.71487928
1H-Indene-2-carboxylic acid, 1-(1,3-benzodioxol-5-yl)-3-(2-(carboxymethoxy)-4-methoxyphenyl)-2,3-dihydro-5-propoxy-, (1S,2R,3S)-		0.713007309
[6]-Gingerdiol 5-O-beta-D-glucopyranoside		-0.705928005
Cholylglutamine		0.702865096
PGP(i-12:0/TXB2)		0.700699517
DG(16:1(9Z)/18:1(9Z)/0:0)	C00165	0.692665527
Tertatolol		0.689891361
2-Aminoacetophenone		-0.689666208
Esmolol	C06980	-0.684621498
Valylaspartic acid		-0.682157
5-Benzylacyclouridine		-0.677238854
L-alpha-Aminobutyric acid	C02356	0.67643668
L-Aspartic acid	C00049	0.664730647
6-(4-(2,2-Di(4-Fluorophenyl)ethylamino)-1-piperidinyl)-N,N'-di-2-propenyl-1,3,5-triazine-2,4-diamine		0.663864479
3-(3-Amino-3-carboxypropyl)uridine		0.662725818
Perfluorooctane sulfonamidoacetic acid		0.661311167
Fallypride		-0.659898534
Hypolaetin 7,3'-dimethyl ether 4'-glucoside		0.655585166
carboxy-ptio		-0.650630067
Z-Lly-fmk		0.644819773
3-(4-Methyl-3-pentenyl)thiophene		-0.639082215
Schiff base		0.636983146
2-Hydroxybutyric acid	C05984	0.636944205

Octaethylene glycol		0.625677225
Aspartylglycosamine	C04540	0.62451503
Gulonic acid	C00800	-0.623203701
Pranidipine		0.622603984
Adenosine monophosphate	C00020	0.614216917
5-Thymidylic acid	C00364	0.612375497
DG(8:0/0:0/20:5(7Z,9Z,11E,13E,17Z)-3OH(5,6,15))		0.610132522
6,9,12,15,18-Tetracosapentaynoic acid		0.605511861
metergoline		0.591010873
CPA(16:0)		0.5874705
phosphoenol pyruvate	C00074	-0.585046318
Methyl l-phenylalaninate		0.580186227
Plumieride		0.576552448
6-Hydroxykaempferol 3,6-diglucoside		0.575149897
Gly-pro-arg-pro		0.5549366
dUMP	C00365	-0.55458557
3-Methyl sulfolene		-0.55172124
Glutamylphenylalanine		-0.544517091
Sucrose phosphate		0.537922452
Pangamic acid		0.537881595
Fluroxene		0.533849262
PA(20:3(6,8,11)-OH(5)/2:0)		0.532610519
2-Heptanethiol		-0.528016495
L-Isoleucine	C00407	-0.526971541
N-(8-Amino-1-carboxyoctyl)-alanyl-proline		-0.519625831
16-dimethylarsinoyl-9Z-hexadecenoic acid		-0.518810273
N-3-oxo-tetradec-7(Z)-enoyl-L-Homoserine lactone		-0.513868441
(2S,3As,6aS)-1-[(2R)-2-carboxy-2-[[(2S)-1-ethoxy-1-oxo-4-phenylbutan-2-yl]amino]acetyl]-3,3a,4,5,6,6a-hexahydro-2H-cyclopenta[b]pyrrole-2-carboxylic acid		0.510109766
5-S-glutathionyl-L-DOPA		0.505381723
Upadacitinib		-0.496632397
4-Dodecylbenzenesulfonic Acid		0.496079915
glas#20		-0.495919031
Coniferaldehyde	C02666	0.49454704
7-hydroxymethotrexate		0.487531061
Eremopetasitenin C3		0.487272587
Berkeleylactone L		-0.486959942
Ethyl cellulose		-0.486358951
L-Threonine	C00188	0.485822864
Carnosine	C00386	0.480280355
2-(2-Thienylmethylene)-1,6-dioxaspiro[4.4]non-3-ene		-0.47507735
3-O-Methyl 5-O-(3-phenylprop-2-enyl) 2,6-dimethyl-4-(3-nitrophenyl)-3,4-dihydropyridine-3,5-dicarboxylate		0.472322626

L-Proline, 1-(1-L-leucyl-L-prolyl)-		-0.466157499
Ulipristal		0.462942481
Naringin dihydrochalcone		0.462608164
Heptadecanoic acid		-0.460985801
D-Proline	C00763	-0.458986969
Aspartyl-Valine		-0.456626872
1-dimethylarsinoyl-decane		0.45114919
Aspartyl-Methionine		-0.445019542
(4-Aminophenyl)phosphonic acid		0.435227261
Asclepin	C08849	-0.421775523
2,9-Dimethyl-4,7-diphenyl-1,10-phenanthroline		-0.419380145
2-p-Tolyl-5,6,7,8-tetrahydrobenzo[d]imidazo[2,1-b]thiazole	C11571	-0.410338126
Naratriptan	C07792	-0.397985993
Ricinoleic acid	C08365	-0.394178852
Hexoprenaline		0.393713435
Botrydial	C09622	0.390118561
Thiabendazole	C07131	0.384054404
3-Methyl-5-pentyl-2-furanpentanoic acid		0.38261758
4-Chloro-L-phenylalanine		0.382441042
1-naphthoate		0.38014432
Perflutren		0.378351876
JP83		0.377600577
Stearaldehyde	C01838	0.360370185
Bisazobiphenyl		-0.357774097
Maltotriose	C01835	0.356755238
Carboxydextran		0.352669993
N-Undecylbenzenesulfonic acid		0.352224384
bhas#32		0.351524028
Cabastine		0.325996469
Armillaripin		0.32295185
Niflumic Acid	C13698	-0.321711865
2-Azaspiro[4.5]decan-3-one		0.316669145
L-Allothreonine	C05519	0.316582033
FAD	C00016	-0.309058637
5-(4-Chloro-3-hydroxy-1-butynyl)-2,2'-bithiophene		0.307563756
gamma-Glutamylaspartic acid		-0.299978001
ADP	C00008	-0.295863543
Berkeleylactone J		0.294803497
Aspartyl-Gamma-glutamate		0.293861769
DEOXYSAPPANONE B 7,3'-DIMETHYL ETHER		0.284051625
7-dehydrocholesterol-d7		0.282156491
Trifluoroacetic acid		0.273868766
Methylenediphosphonic acid		0.264202214
gamma-Glutamylglycine		0.257619342

Carbidopa	C07562	-0.257220842
5-Aminopentanoic acid	C00431	-0.255371436
Cohibin C		-0.237683896
Gossypetin 8-glucuronide 3-sulfate		0.214807811
Vigabatrin	C07500	-0.202050513
Aspartyl-Tyrosine		0.201944633
5-(5-Iodo-4-methoxy-2-propan-2-ylphenoxy)pyrimidine-2,4-diamine		0.193178996
(3R,4S,5S,6S)-6-[4-Chloro-2-(furan-2-ylmethylamino)-5-sulfamoylbenzoyl]oxy-3,4,5-trihydroxyoxane-2-carboxylic acid		0.189505828
Glucoiberverin		0.189175482
9,14-dihydroxy-octadecanoic acid		0.18646116
2-hydroxy-3-butenylglucosinolate		0.182333113
7-keto palmitic acid		0.179612156
Glucoberteroin		0.179035129
Nitrocefin		0.174834026
Dehydrochorismic acid		0.174770414
Calcium ascorbate		0.173479879
1-a,24R,25-Trihydroxyvitamin D2		0.172634037
AM2201 N-(4-hydroxypentyl) metabolite		0.168258038
Pent-1-en-2-ol		0.168166352
4-methylthiobutyl glucosinolate		0.167380068
Terbufos	C18693	0.15295596
Adrenic acid	C16527	0.143164018
Tris(2-butoxyethyl) phosphate	C14446	0.134185154
